# Active RB causes visible changes in nuclear organization

**DOI:** 10.1083/jcb.202102144

**Published:** 2022-01-12

**Authors:** Badri Krishnan, Takaaki Yasuhara, Purva Rumde, Marcello Stanzione, Chenyue Lu, Hanjun Lee, Michael S. Lawrence, Lee Zou, Linda T. Nieman, Ioannis Sanidas, Nicholas J. Dyson

**Affiliations:** 1 Massachusetts General Hospital Cancer Center and Harvard Medical School, Charlestown, MA; 2 Broad Institute of Massachusetts Institute of Technology and Harvard, Cambridge, MA; 3 Department of Biology, Massachusetts Institute of Technology, Cambridge, MA

## Abstract

RB restricts G1/S progression by inhibiting E2F. Here, we show that sustained expression of active RB, and prolonged G1 arrest, causes visible changes in chromosome architecture that are not directly associated with E2F inhibition. Using FISH probes against two euchromatin RB-associated regions, two heterochromatin domains that lack RB-bound loci, and two whole-chromosome probes, we found that constitutively active RB (ΔCDK-RB) promoted a more diffuse, dispersed, and scattered chromatin organization. These changes were RB dependent, were driven by specific isoforms of monophosphorylated RB, and required known RB-associated activities. ΔCDK-RB altered physical interactions between RB-bound genomic loci, but the RB-induced changes in chromosome architecture were unaffected by dominant-negative DP1. The RB-induced changes appeared to be widespread and influenced chromosome localization within nuclei. Gene expression profiles revealed that the dispersion phenotype was associated with an increased autophagy response. We infer that, after cell cycle arrest, RB acts through noncanonical mechanisms to significantly change nuclear organization, and this reorganization correlates with transitions in cellular state.

## Teaser

Krishnan et al. use FISH to show that specific forms of RB induce changes in the organization of euchromatin and heterochromatin domains. These changes are visible under the microscope, occur after cell cycle arrest, are separable from senescence, and represent an E2F-independent activity of RB.

## Introduction

The best-known molecular function of RB (the protein product of the retinoblastoma tumor susceptibility gene) is the regulation of E2F-dependent transcription (Burkhart and Sage, 2008; Dyson, 1998; Dyson, 2016). E2F controls the expression of several hundred genes that are needed for cell proliferation. RB directly binds to the activation domains of E2F proteins and recruits repressor complexes to E2F-regulated promoters. During the G1 to S phase transition, the temporal activation of cyclin-dependent kinases (CDKs) leads to the hyperphosphorylation of RB, the relief of E2F-mediated repression, and the induction of E2F-mediated activation of genes. In this way, CDKs initiate a wave of E2F-dependent transcription of genes required for cell proliferation (Harbour and Dean, 2000a, b; Hinds et al., 1992; Sherr, 1996). In agreement with this model, chromatin immunoprecipitation sequencing (ChIP-seq) experiments confirm that RB binds to the promoters of many E2F-regulated genes.

Perhaps less well known is that ChIP-seq experiments show an extensive distribution of RB-binding sites that extends far behind the conventional set of E2F-regulated genes (Chicas et al., 2010; Ishak et al., 2016; Kareta et al., 2015). These studies identified RB-binding sites in promoters of genes with diverse functions, in repetitive sequence elements, in intergenic sequences, and at locations within genes (Chicas et al., 2010; Ishak et al., 2016; Kareta et al., 2015). While the effects of RB on classic E2F-regulated promoters have been studied in detail, the roles of the many additional RB-binding sites scattered through the genome remain unclear. There are many potential scenarios. At one extreme is the possibility that only RB binding to cell cycle–regulated promoters has biological impact, and that the additional RB-binding sites exist but play no role. An alternative view, discussed recently (Dick et al., 2018), is that RB has multiple roles: a canonical role at cell cycle–regulated promoters and several noncanonical roles that include repression of transcription at repetitive sequence elements. Among the noncanonical roles proposed for RB is the idea that it affects chromosome architecture. Physical interactions between RB and Condensin II proteins (Coschi et al., 2014; Kim et al., 2021; Longworth et al., 2008) and effects of RB on chromosome cohesion (Isaac et al., 2006; Manning et al., 2010; Manning et al., 2014; van Harn et al., 2010) led to speculation that RB may help to organize elements of chromosome structure (Longworth and Dyson, 2010; Marshall et al., 2020). However, currently there is limited evidence that RB controls the organization of chromosomal domains. If such a role does exist, it is unknown whether this is a constitutive property of RB or one that appears only in specific contexts.

To answer these questions, we used FISH probes and took advantage of oligopaint technology (Beliveau et al., 2014; Beliveau et al., 2012) to look for RB-dependent changes in the organization of large chromosomal regions and whole chromosomes. Our results show that active forms of RB alter the organization of chromosomal regions. Expression of constitutively unphosphorylated ΔCDK-RB was sufficient to cause dispersion of euchromatic and heterochromatic regions. Similar effects were visible in cells undergoing cell cycle exit during irradiation (IR)-induced senescence and during long-term exposure to CDK4/6 inhibitor, two types of RB-mediated arrest with persistent unphosphorylated RB, and were visible in both RB-bound and RB-free loci. These changes were not prevalent when cells were simply arrested in G1, but they accumulated in a time-dependent manner and were triggered by expression of specific forms of RB, suggesting that they require specific cellular conditions and specific properties of RB. Chromatin reorganization was also evident using whole-chromosome probes, and ΔCDK-RB expression caused the relocalization of chromosome 19 to nucleolar periphery. Collectively, these results show that RB does indeed cause extensive changes in nuclear organization, and that these occur during stable cell cycle exit. Unexpectedly, gene expression analysis revealed that RB-induced chromatin dispersion was associated with an increase in autophagy flux, suggesting that RB-induced chromatin changes are coordinated with other cellular changes. These results demonstrate that, in addition to its canonical roles in E2F regulation, the sustained expression of active RB causes substantial changes in chromosomal organization and nuclear architecture, with consequences for cellular expression program and state.

## Results

### Quantification of FISH signals using skeleton dot lengths

**Figure 1.**
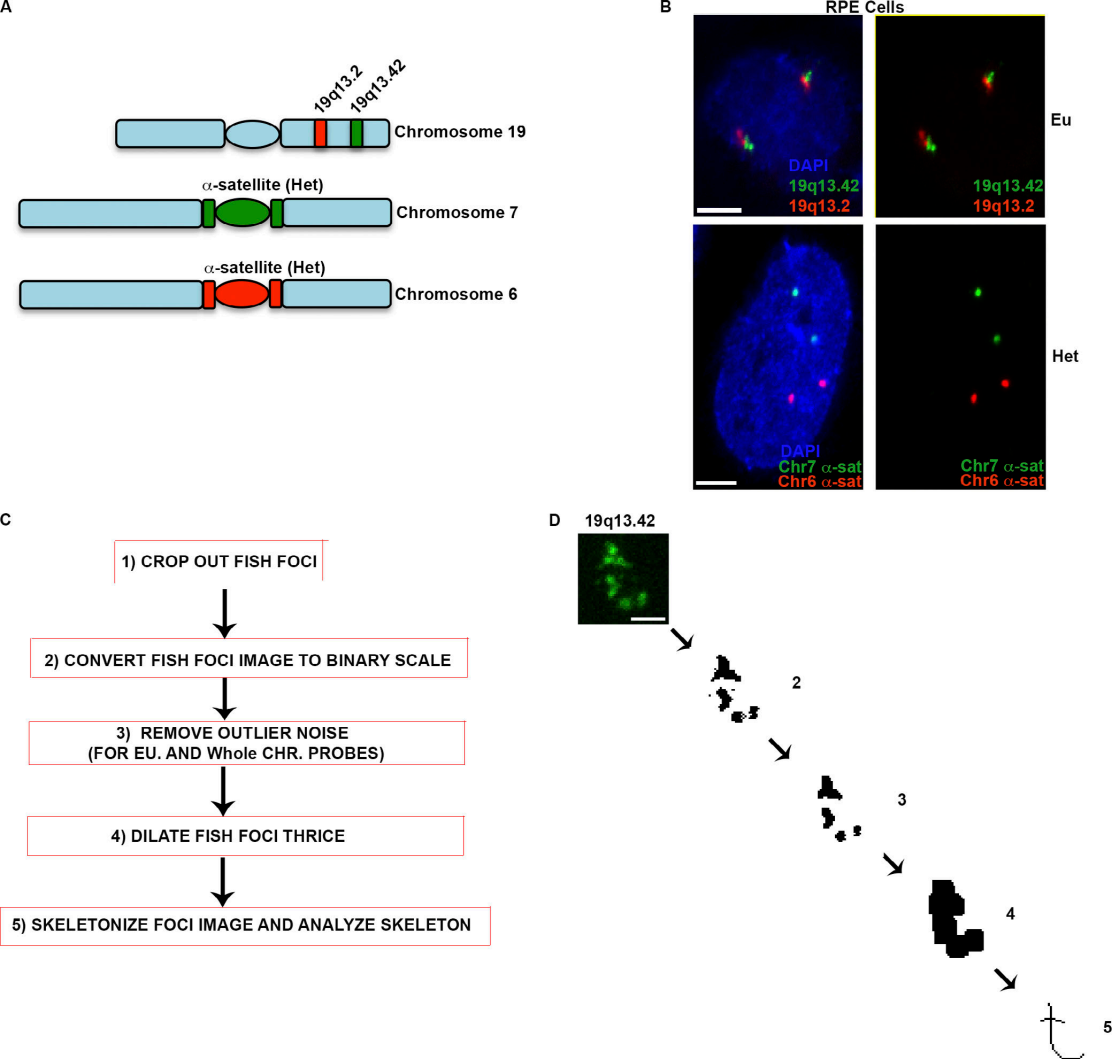
**Visualizing large chromosomal regions using FISH probes and analysis of FISH signals. (A)** Chromosomal (Chr) location of euchromatin (Eu) and heterochromatin (Het) FISH probes used in this study. **(B)** Dual-color FISH showing the nuclear appearance and location of two heterochromatin and euchromatin probes. **(C)** Steps for generating skeleton dot images and calculating skeleton dot lengths. **(D)** An example outcome of executing the steps in C. Macro was created and executed using Fiji. Scale bar for B = 5 µM; for D = 2 µm.

To assess chromatin organization, we took a visual approach and performed FISH experiments to detect large chromosomal regions. We used probes against two euchromatin regions on chromosome 19 (19q13.42 and 19q13.2; 2.7 and 4.7 MB in size, respectively) and two heterochromatin regions (chromosome 6 and 7 α-satellite; 4.6 and 3.7 MB, respectively). These gave two clearly separated signals in nuclei of WT RPE1 cells (Fig. 1, A and B), but each set of FISH probes produced foci that varied in appearance, with clear differences in size and shape. To quantify these features, we created a skeleton dot image of each signal and measured dot lengths (Fig. 1, C and D). This effectively captured multiple aspects of the foci and allowed us to quantify differences in punctation, branching, and overall length of the signals.

**Figure 2.**
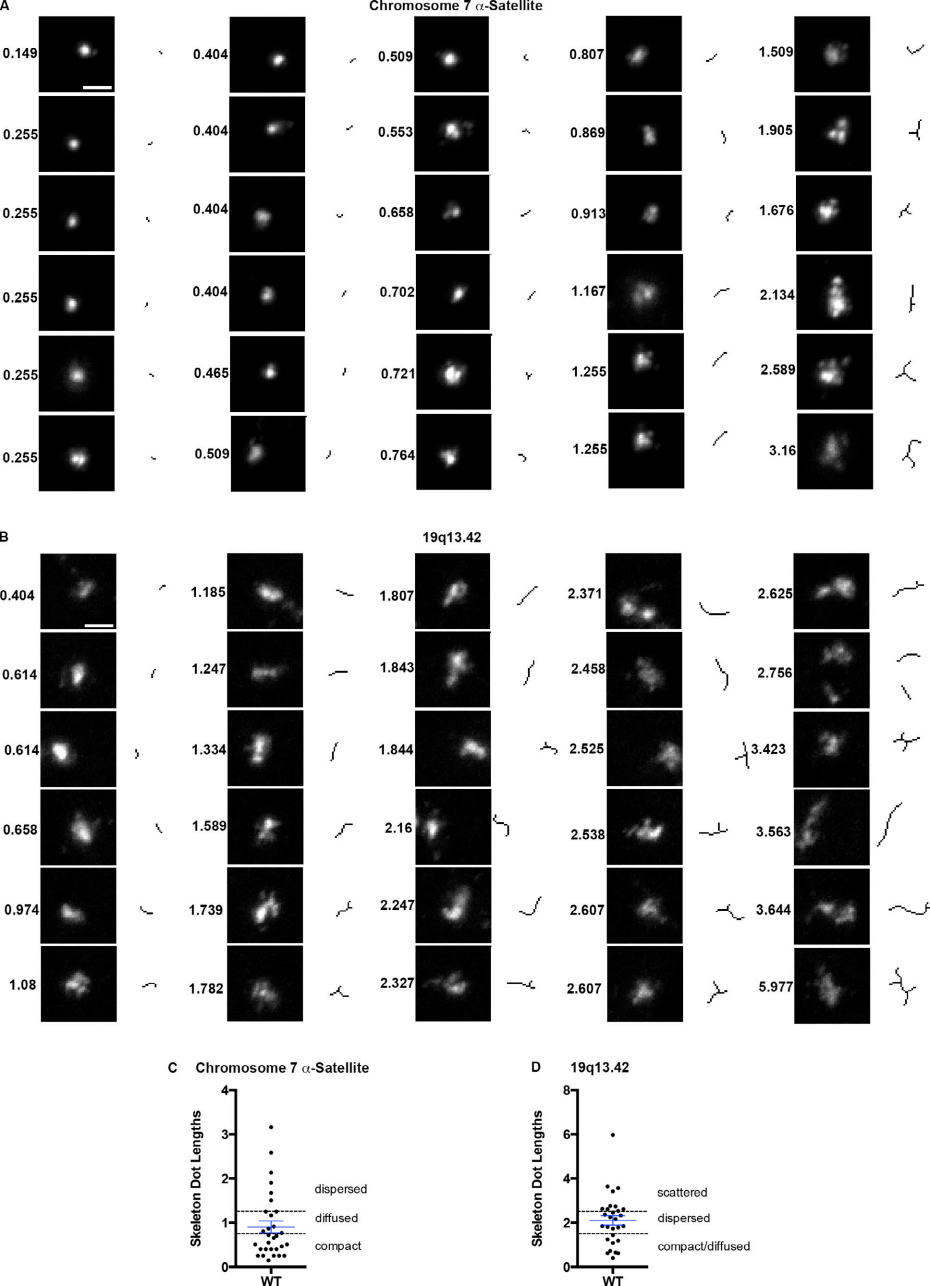
**FISH probes give a range of signal types/appearance in WT RPE cells. (A and B)** Montage of chromosome 7 α-satellite (A) or 19q13.42 (B) FISH signals obtained from a single image field, with corresponding skeleton images and skeleton lengths. The images were arranged in ascending order of skeleton length. Note the change in shape, punctation, and length of FISH signals with the increase in the skeleton dot length scores. **(C and D)** shows the distribution of skeleton dot lengths obtained for chromosome 7 α-satellite and 19q13.42 FISH signals in WT RPE cells. Dashed lines indicate the cutoffs for defining the categories compact, diffused, and dispersed for chromosome 7 α-satellite and compact/diffused, dispersed, and scattered for 19q13.42 probe. Numbers of foci quantified for each sample (*n*) are as follows (in the order they appear on the bar graphs): C, *n* = 30; D, *n* = 30. Scale bar = 2 µm.

Fig. 2, A and B, illustrates the range of signals obtained with probes to chromosome 7 α-satellite and 19q13.42 and in a single field of WT RPE1 cells and shows the corresponding skeleton dot image and lengths of each focus. We arranged the signals detected with the chromosome 7 α-satellite probe in an ascending order based on dot length score and noted that the increase in length matched with the transition from “spherical, bright spot” to “amorphous-low bright spot” to “punctate/compartmentalized-elongated-low-high bright spot.” We termed these categories “compact,” “diffused,” and “dispersed,” respectively (Fig. 2 A). For ease of classification, we set a numerical cutoff at points where we believed that the visual transitions occurred. When we performed the same exercise using the19q13.42 probe (Fig. 2 B), we noted that the signals from this euchromatic region were generally less compact and more diffuse/dispersed than the heterochromatin foci. Therefore, we added another category of extensively punctate or elongated foci, termed “scattered.” Plots of the skeleton dot lengths (Fig. 2, C and D) show the distribution of the signals and the cutoff values that we selected based on the visual changes. We followed this methodology for additional probes (including 19q13.2 and chromosome 6 α-satellite) and used this approach to quantify changes in all of the experimental conditions described below.

### Changes in chromatin organization are visible in cells undergoing RB-mediated arrest

**Figure 3.**
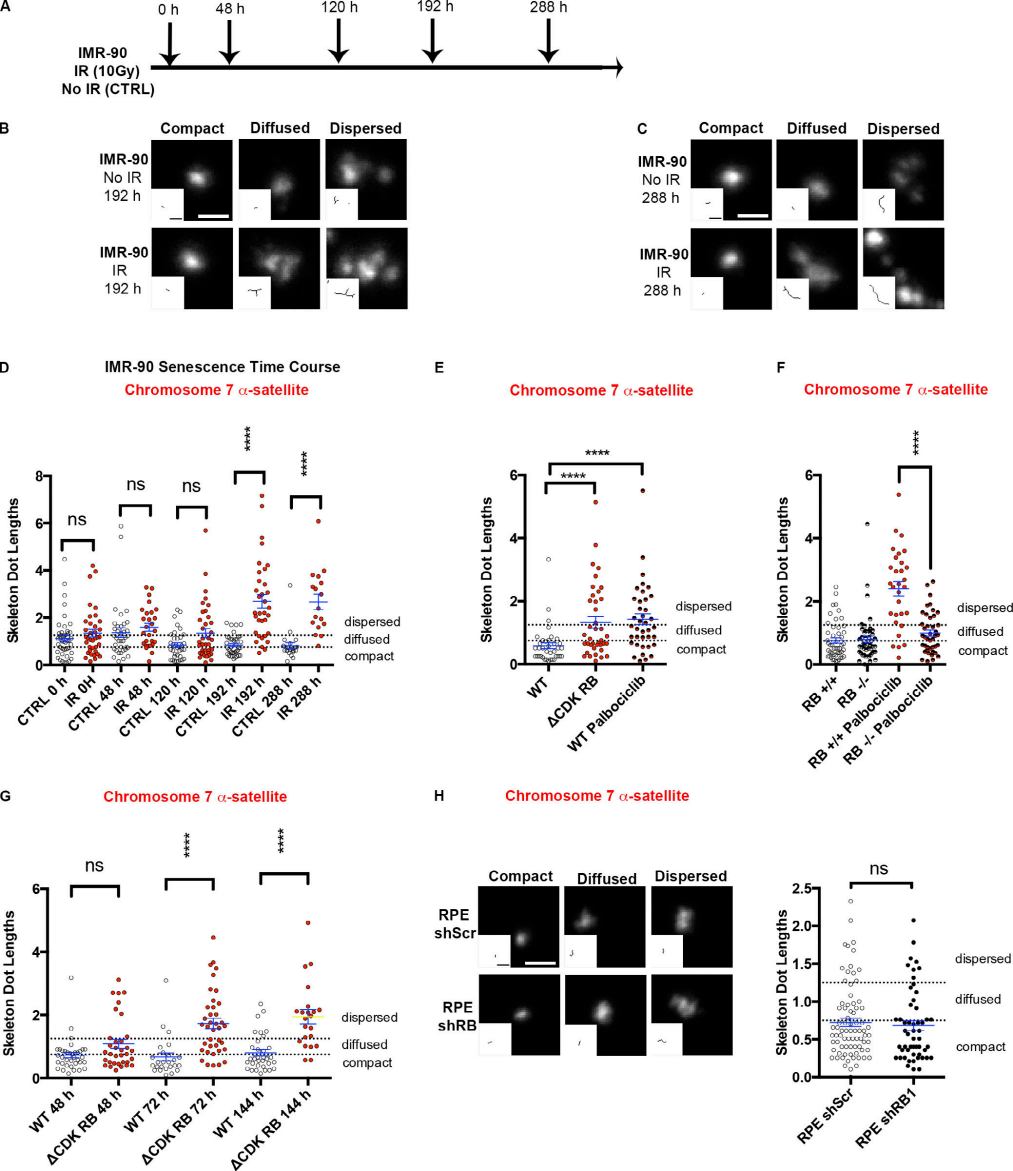
**Dispersion of FISH signals, a time-dependent change that occurs during IR-induced senescence and in palbociclib-treated cells.** **(A)** IR was used to induce senescence in IMR-90 cells. Dispersion was assessed by measuring the skeleton dot lengths of the chromosome 7 α-satellite FISH signal at the indicated time points. CTRL, control. **(B and C)** Dispersion was observed to increase at 192 h (B) and 288 h (C) after IR treatment (insets show skeletons of representative foci). **(D)** Distribution of individual skeleton dot lengths of the FISH signals measured in A. The mean skeleton length and the shift up in distribution of individual measurements demonstrate the increase in dispersion at 192 and 288 h after IR. **(E)** Skeleton dot length measurements of the FISH signal of the chromosome 7 α-satellite probe in WT RPE1 cells treated with palbociclib for 72 h or cells expressing ΔCDK-RB for 72 h. Palbociclib treatment disperses FISH signal in WT RPE to a degree comparable to 72 h of ΔCDK-RB expression. **(F)** Skeleton dot length measurements in chromosome 7 α-satellite region in RB^+/+^ (WT) or RB^−/−^ (CRISPR knockout) cells treated with palbociclib for 5 d. Chromatin dispersion is evident only in RB^+/+^ (WT) cells. **(G)** Dispersion caused by ΔCDK-RB expression became statistically significant after 72 h and continued to increase to 144 h. **(H)** The range of FISH signals obtained for shRNA-mediated knockdown of RB1 and cells expressing scrambled shRNAs in RPE1 cells. Quantitation of mean skeleton dot lengths showed no significant difference in dispersion between RB1 shRNA knockdown and scrambled shRNA samples. Numbers of foci quantified for each sample (*n*) are as follows (in the order they appear on the bar graphs): D, *n* = 46, 37, 36, 27, 35, 37, 38, 34, 24, 17; E, *n* = 37, 38, 39; F, *n* = 48, 47, 30, 42; G, *n* = 36, 34, 25, 38, 33, 22; H, *n* = 78, 53. Error bars are SEM. Nonparametric two-tailed Mann–Whitney *U* tests were performed for pairs of samples indicated on graphs D–H, and asterisks denote P values; ns, P > 0.05; ****, P ≤ 0.0001. Scale bar for B, C, and H (including insets) = 2 µm. Dashed lines indicate the cutoffs for defining the categories compact, diffused, and dispersed for chromosome 7 α-satellite.

**Figure S1.**
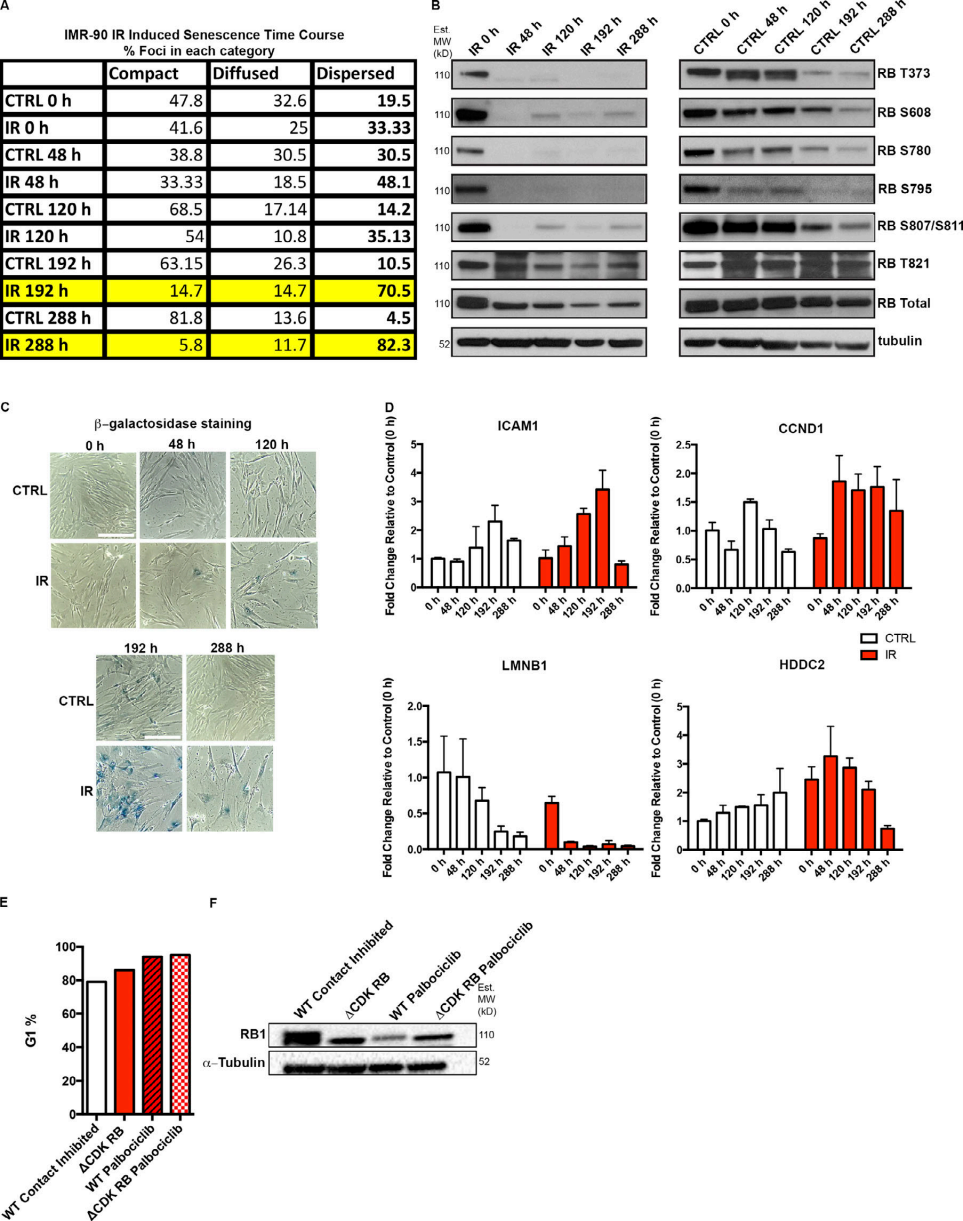
**RB activation in irradiated or palbociclib treated cells. (A)** Percentage of foci classified into different categories of skeleton dot lengths in IMR-90 cells from the experiment described in Fig. 3 D. Highlighted rows show time points at which a major increase in dispersion was observed. CTRL, control. **(B)** The changes in RB phosphorylation during IR-induced senescence in IMR-90 cells. Western blots show a major and prolonged loss of RB phosphorylation at all the tested sites, observed first 48 h after IR treatment and through the time course for IR-treated cells. Nontreated cells show reduction in some RB phosphorylation forms at later time points (192 and 288 h) owing to contact inhibition–induced G1 arrest but retain overall phosphorylation. MW, molecular weight. **(C)** β-Galactosidase staining of the same populations of cells. Scale bar = 190 µm. **(D)** Changes in expression of key IR-induced senescence signature genes over the time course in IMR-90 cells. Four genes, ICAM1, CCND1, LMNB1 and HDDC2, show dynamic changes as the cells progress toward senescence. Graphs show fold-changes (enrichment over 0-h time point) for the four genes in CTRL (untreated) and IR-treated samples. Three technical replicates per sample were used to calculate fold-changes. **(E)** Percentage of G1 cells for WT RPE contact-inhibited cells, ΔCDK-RB cells, and both treated with palbociclib for 72 h. **(F)** Western blot analysis of the cells in E with the indicated antibodies. Note the presence of only the unphosphorylated RB form (lower molecular weight band) in ΔCDK-RB, palbociclib-treated WT, and ΔCDK-RB samples. Source data are available for this figure: SourceData FS1.

To examine the hypothesis that active RB influences the organization of large chromosomal regions, FISH was performed on cells induced to enter senescence. We chose this cellular context because previous studies have shown that oncogene-induced senescence is RB dependent and have also described reorganization of large chromosomal regions to form senescence-associated heterochromatic foci (SAHF) in some cell types (Chicas et al., 2010; Criscione et al., 2016; Narita and Lowe, 2005; Narita et al., 2003; Zirkel et al., 2018). IR was used to induce senescence in IMR-90 human fibroblasts, and the appearance of the FISH signal was recorded at various time points after treatment (Fig. 3 A). Representative images for IMR-90 cells at 192 and 288 h after IR are shown in Fig. 3, B and C. Skeleton dot lengths were measured to quantify the differences between IR-treated and control cells, and we counted the number of foci in each category. An increase in dispersed foci in chromosome 7 α-satellite was observed 192 and 288 h after IR (Fig. S1 A), together with a threefold increase in average skeleton dot length (Fig. 3 D). Several changes in RB phosphorylation occurred soon after IR (Fig. S1 B), and the increase in dispersion coincided roughly with the appearance of β-galactosidase–stained cells (Fig. S1 C). IR-treated IMR-90 cells also had progressively steeper senescence-associated secretory phenotype–associated transcriptional changes when compared with non-IR samples (Fig. S1 D).

To ask whether similar changes in organization occur in other forms of RB-mediated arrest, we examined RPE1 cells treated with the CDK4/6 inhibitor palbociclib (Fry et al., 2004; Sherr et al., 2016; Toogood et al., 2005). Palbociclib causes an RB-mediated arrest in which cells accumulate in G1, expressing unphosphorylated RB (Fig. S1, E and F). Palbociclib treatment of RPE1 cells induced dispersion of chromosome 7 α-satellite (Fig. 3 E), similar to the changes observed in IR-treated IMR-90 cells. However, palbociclib treatment (5 µM) failed to induce any dispersion in CRISPR-generated RB-knockout RPE1 cells (Nicolay et al., 2015), even after 5 d, whereas it readily induced dispersion in matched control cells (Fig. 3 F).

**Figure S2.**
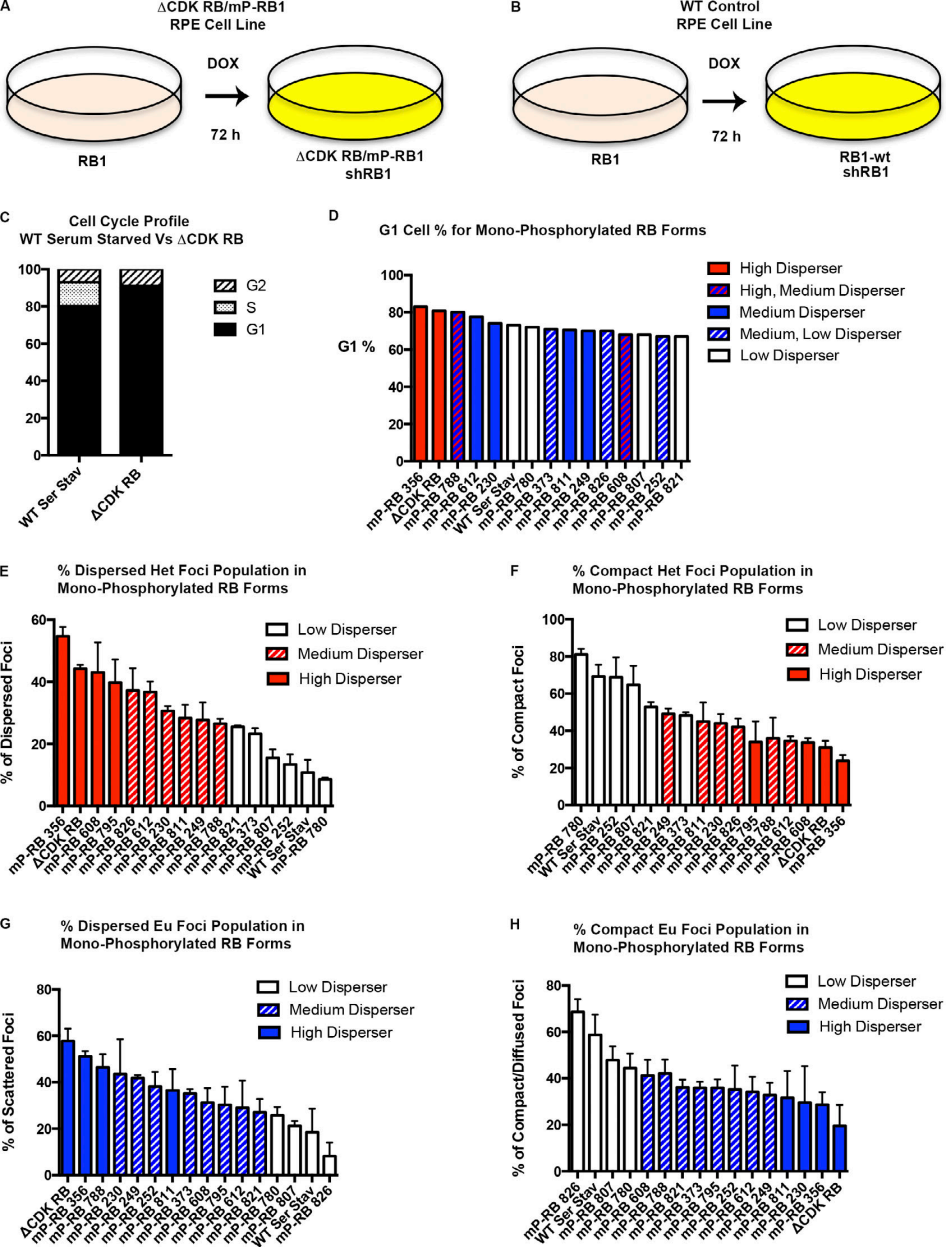
**Relationship between cell cycle stages and dispersion/scattering caused by various forms of active RB. (A and B)** DOX-inducible active RB system in RPE1 cells. Addition of DOX causes the simultaneous expression of sh*RB1*, which targets the 3′ UTR of *RB1* and depletes endogenous RB protein, and exogenous mutant RB alleles (unphosphorylated ΔCDK-RB or any of the 14 mP-RB forms; A) or WT RB (B). **(C)** Cell cycle profile for ΔCDK-RB and WT RPE cells serum starved for 3 d (WT Ser Stav). **(D)** Percentage of G1 cells after 72-h DOX induction of mP-RB forms and ΔCDK-RB. Bar color and pattern depict categorization of dispersion phenotype for mP-RB forms and ΔCDK-RB. Red, blue, and white bars represent high, medium, and low disperser for both euchromatin and heterochromatin regions. Interleaved bars indicate different classifications for heterochromatin and euchromatin dispersion/scatter phenotype. **(E and F)** Percentage of dispersed and compact heterochromatin (Het) foci for the mP-RB forms and ΔCDK-RB. Bar color and pattern show intensity of dispersion phenotype, which was classified based on mean skeleton dot lengths. **(G and H)** Percentage of scattered and compact euchromatin (Eu) foci for the mP-RB forms and ΔCDK-RB. Bar color and pattern show intensity of scatter phenotype, which was classified based on mean skeleton dot lengths. Quantitation of dot lengths was from two independent biological replicates, set up and performed on different days. Error bars are SEM. Numbers of foci quantified for each sample (*n*) are as follows: E and F, *n* = 55 (356), 61 (ΔCDK-RB), 74 (608), 48 (795), 75 (826), 50 (612), 76 (230), 75 (811), 104 (249), 60 (788), 63 (821), 56 (373), 93 (807), 85 (252), 207 (WT Ser Stav), 115 (780); G and H, *n* = 84 (356), 60 (ΔCDK-RB), 52 (788), 113 (811), 124 (230), 144 (249), 66 (373), 120 (795), 80 (252), 80 (608), 106 (821), 56 (612), 145 (780), 69 (807), 99 (826), 111 (WT Ser Stav).

**Figure S3.**
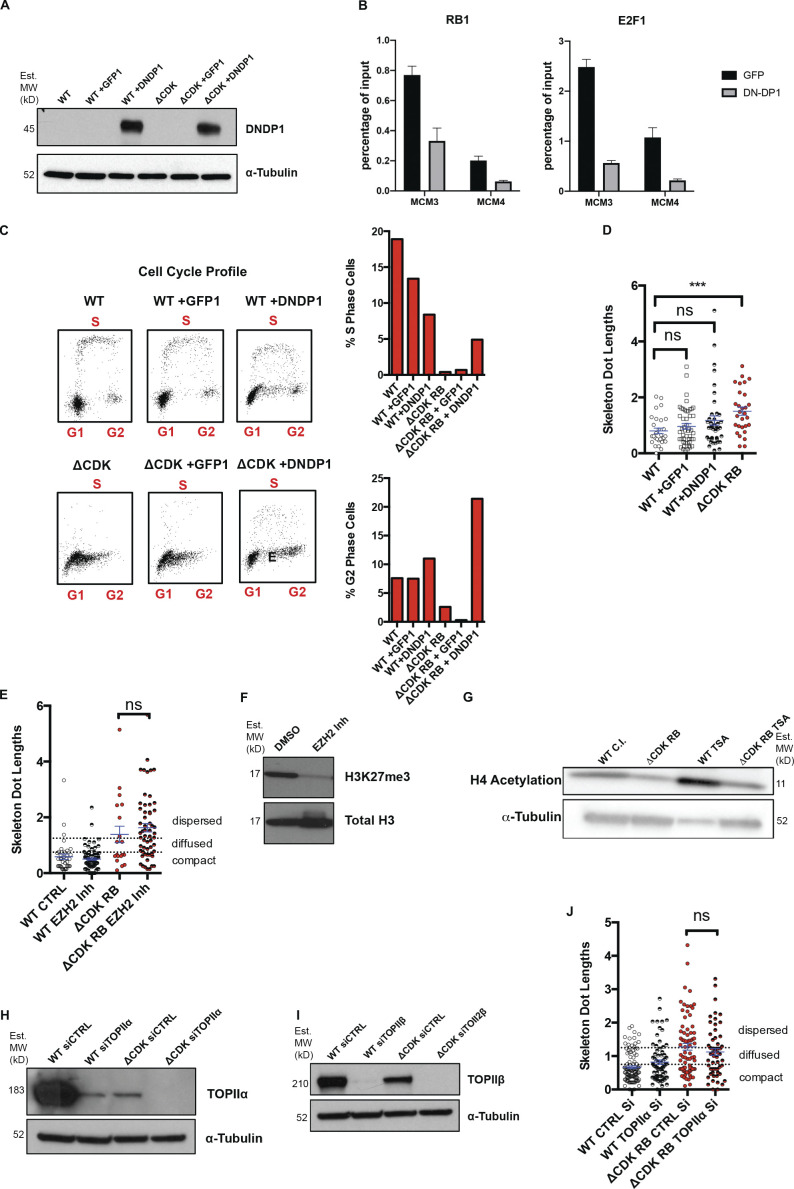
**Investigation of the role of DP1, EZH2, HDAC, TOPIIα, and TOPIIβ in RB-mediated dispersion. (A)** Western blots of WT and ΔCDK-RB RPE cells expressing GFP or DNDP1. MW, molecular weight. **(B)** E2F ChIP-qPCR and RB1 ChIP-qPCR showing that expression of DNDP1 decreased E2F1 and RB1 binding to sites in the MCM3, MCM4 promoters, compared with cells expressing only GFP. Average scores from three technical replicates were calculated per sample and per epitope. Holm–Sidak multiple *t* test was performed, and asterisks denote P values. Scale bar = 25 µm. **(C)** Effect of DNDP1 expression on cell cycle profile of the cells in A. Profile shows G1, S, and G2 cells in the different samples. DNDP1 expression in ΔCDK-RB cells interfered with G1 arrest and increased the percentage of cells in S and G2. **(D)** Effect of DNDP1 expression on dispersion. Quantitation of mean skeleton dot lengths after DNDP1 expression in WT RPE cells. Expression of DNDP1 in WT RPE does not cause a significant increase in mean skeleton dot length when compared with WT RPE cells or GFP-expressing WT RPE cells. **(E)** Mean skeleton dot lengths (chromosome 7 α-satellite probe) after WT and ΔCDK-RB RPE cells were treated with EZH2 inhibitor. **(F)** Western blot for WT RPE cells treated with DMSO or EZH2 inhibitor (Inh.). Note that treatment with EZH2 inhibitor reduces H3K27 trimethylation levels. **(G)** Western blot for WT contact inhibited (C.I.) RPE and ΔCDK-RB treated with TSA for 72 h. H4 acetylation in both WT C.I. and ΔCDK-RB cells increases after TSA treatment. **(H and I)** Western blot for WT RPE and ΔCDK-RB transfected with control and TOPIIα (H) or TOPIIβ (I) siRNAs. siRNA-mediated knockdown of TOPIIα reduced the levels of the appropriate endogenous protein in WT and ΔCDK-RB cells. We note that TOPIIα was expressed at lower levels in cells expressing ΔCDK-RB compared with WT RPE cells. TOPIIβ siRNA-mediated knockdown causes complete loss of endogenous TOPIIβ in both WT and ΔCDK-RB cells. It was also observed that TOPIIβ levels were lower in ΔCDK-RB, compared with WT. **(J)** Mean skeleton dot lengths (chromosome 7 α-satellite probe) after WT and ΔCDK-RB RPE cells were treated with control and TOPIIα siRNAs. Numbers of foci quantified for each sample (*n*) are as follows (in the order they appear on the bar graphs): D, *n* = 29, 48, 40, 29; E, *n* = 37, 82, 19, 58; J, *n* = 103, 75, 75, 56. Error bars are SEM. Nonparametric two-tailed Mann–Whitney *U* test was performed for pairs of samples indicated on graphs, and asterisks denote P values. ns, P > 0.05; ***, P ≤ 0.001. Source data are available for this figure: SourceData FS3. Dashed lines indicate the cutoffs for defining the categories compact, diffused, and dispersed for chromosome 7 α-satellite.

To test whether active RB is not just necessary but sufficient to cause dispersion, we used a recently described set of RPE1-derived cell lines engineered such that addition of doxycycline (DOX) induces the knockdown of endogenous RB and its replacement by FLAG-tagged versions of RB (Sanidas et al., 2019; Fig. S2, A and B). With this system, we examined the effects of expressing ΔCDK-RB, a form of RB that cannot be inactivated by CDK phosphorylation and that has unchecked activity when expressed in rapidly proliferating cells. Induction of ΔCDK-RB led to dispersion of the FISH signal. Although ΔCDK-RB arrests cells in G1 within 24 h, the dispersion of the chromosome 7 α-satellite signal began to appear only after 48 h and did not give a statistically significant increase in mean skeleton dot lengths until 72 h (Fig. 3 G). In contrast, no increase in dispersion or average dot length was observed when cells were induced to replace endogenous RB protein with FLAG-tagged WT RB or were depleted of RB (Figs. S2 B and 3 H). Unlike IR-treated IMR-90 cells, ΔCDK-RB–induced dispersion in RPE1 cells was evident in the absence of senescence markers (such as senescence-associated β-galactosidase, SAHFs, or senescence-associated transcription signatures). We conclude that sustained expression of active RB is sufficient to change the organization of the chromosome 7 α-satellite region.

### ΔCDK-RB alters the chromatin organization of both euchromatin and heterochromatin regions

**Figure 4.**
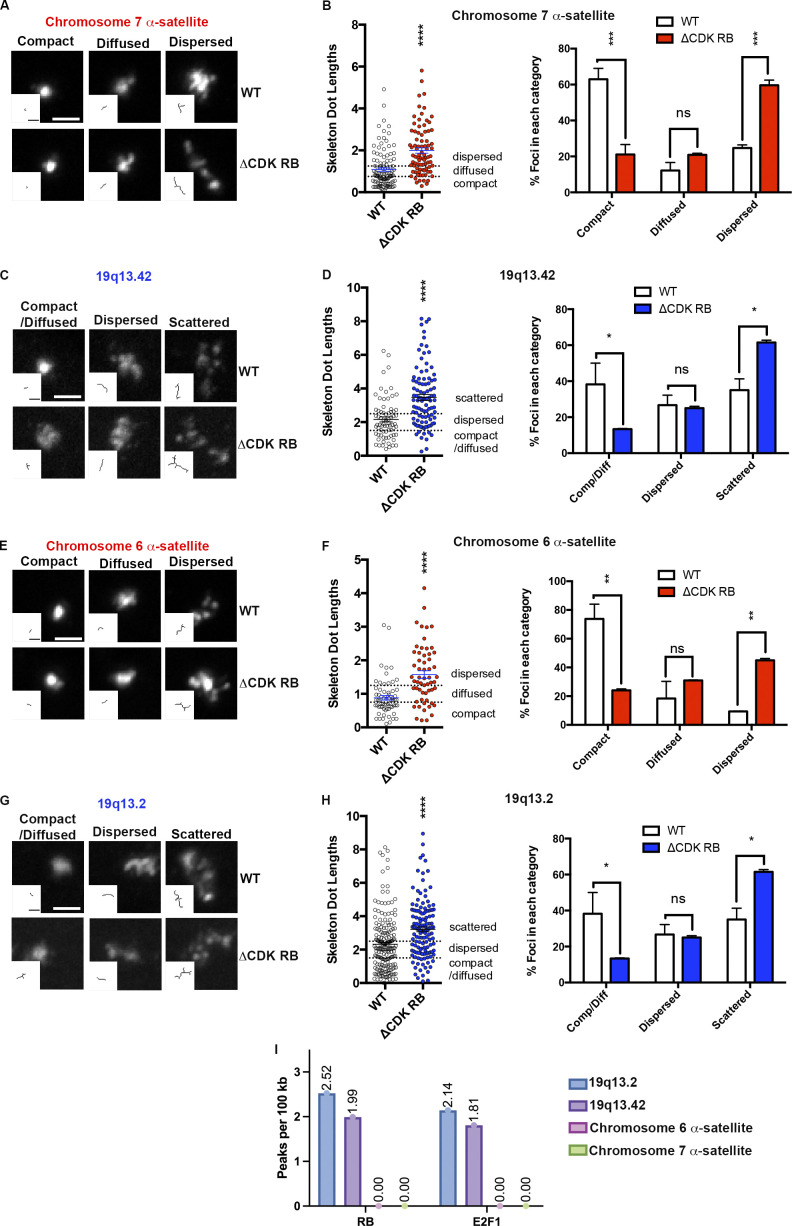
**ΔCDK-RB induces dispersion and scattering in both heterochromatin and euchromatin regions. (A and E)** Images of foci categorized as compact, diffused, and dispersed detected using probes to chromosome 7 and chromosome 6 α-satellite heterochromatin. Skeleton signals are shown in inset. **(B and F)** Percentage of foci in each category and mean skeleton dot length for these heterochromatin regions. **(C and G)** Images of foci categorized as compact/diffused, dispersed, and scattered detected using probes to 19q13.42 and 19q13.2. Skeleton signals are shown in inset. **(D and H)** Scatter plots depicting distribution of individual skeleton dot lengths and percentage of foci in each category for these regions of euchromatin. Note that ΔCDK-RB–expressing cells have significantly higher mean skeleton dot lengths, shifted-up distribution of skeleton dot lengths, and significantly higher percentages of cells with dispersed/scattered foci. Quantitation is from two independent biological replicates, set up and performed on different days. **(I)** Number of RB- and E2F-bound peaks per 100 kb in the four tested regions. RB- or E2F-bound chromatin loci were detected only in the two euchromatin regions. Numbers of foci quantified for each sample (*n*) are as follows (in the order they appear on the bar graphs): B, *n* = 112, 84; D, *n* = 72, 97; F, *n* = 66, 59; H, *n* = 191, 137. Error bars are SEM. For B, D, F, and H (left panels), asterisks denote P values from nonparametric two-tailed Mann–Whitney *U* test. For B, D, F, and H (right panels), asterisks denote P values from nonparametric two-tailed multiple *t* test (without correction for multiple comparisons). ns, P > 0.05; *, P ≤ 0.05; **, P ≤ 0.01; ***, P ≤ 0.001; ****, P ≤ 0.0001. Scale bar (including insets) = 2 µm. Dashed lines indicate the cutoffs for defining the categories compact, diffused, and dispersed for chromosome 7 α-satellite and chromosome 6 α-satellite and compact/diffused, dispersed, and scattered for 19q13.42 and 19q13.2 probe.

To ask whether similar effects of RB activation are visible at other genomic loci, we used two euchromatic probes (19q13.42 probe and 19q13.2) and a second heterochromatin probe that detects the α-satellite regions on chromosome 6. Measurement of skeleton dot lengths revealed that ΔCDK-RB had a consistent effect in all four regions examined (Fig. 4). ΔCDK-RB expression increased the mean skeleton dot length measured with each probe and shifted the distribution of skeleton dot lengths (Fig. 4, B, D, F and H, left panels). ΔCDK-RB expression also reduced the percentage of compact foci detected by each probe and increased the percentage of foci that were dispersed or scattered (Fig. 4, B, D, F and H, right panels). Because heterochromatin probes (Fig. 4, A and E) gave signals that were more compact than euchromatin probes (Fig. 4, C and G), the changes upon RB activation were easiest to discern in heterochromatic regions. Note, for example, that heterochromatic probes had the highest percentage of compact foci (>60%) in control cells, while euchromatic probes showed the highest percentage of scattered foci in cells expressing ΔCDK-RB.

Examination of RB ChIP-seq data revealed an important difference between the euchromatic and heterochromatic regions probed here. While the euchromatic probes 19q13.2 and 19q13.42 contain 119 and 54 RB peaks, respectively (2.52 and 1.99 RB bound loci per 100 kb), the regions targeted by the chromosome 6 and 7 α-satellite probes contain 0 RB peaks and 0 E2F1 peaks (Fig. 4 I). This suggests that the chromatin dispersion induced by active RB is not restricted to regions that are directly bound by RB. Given a report that mouse RB has an affinity for repetitive sequences that is not readily detected by ChIP-seq (Ishak et al., 2016), it is formally possible that there is some form of RB association with the heterochromatin domains. Nevertheless, it is clear that the chromatin dispersion phenotype can be uncoupled from the direct effects of RB, mediated via canonical E2F/RB binding sites.

### The dispersed/scattered phenotype is not a universal feature of G1 cells but is promoted by specific forms of active RB

**Figure 5.**
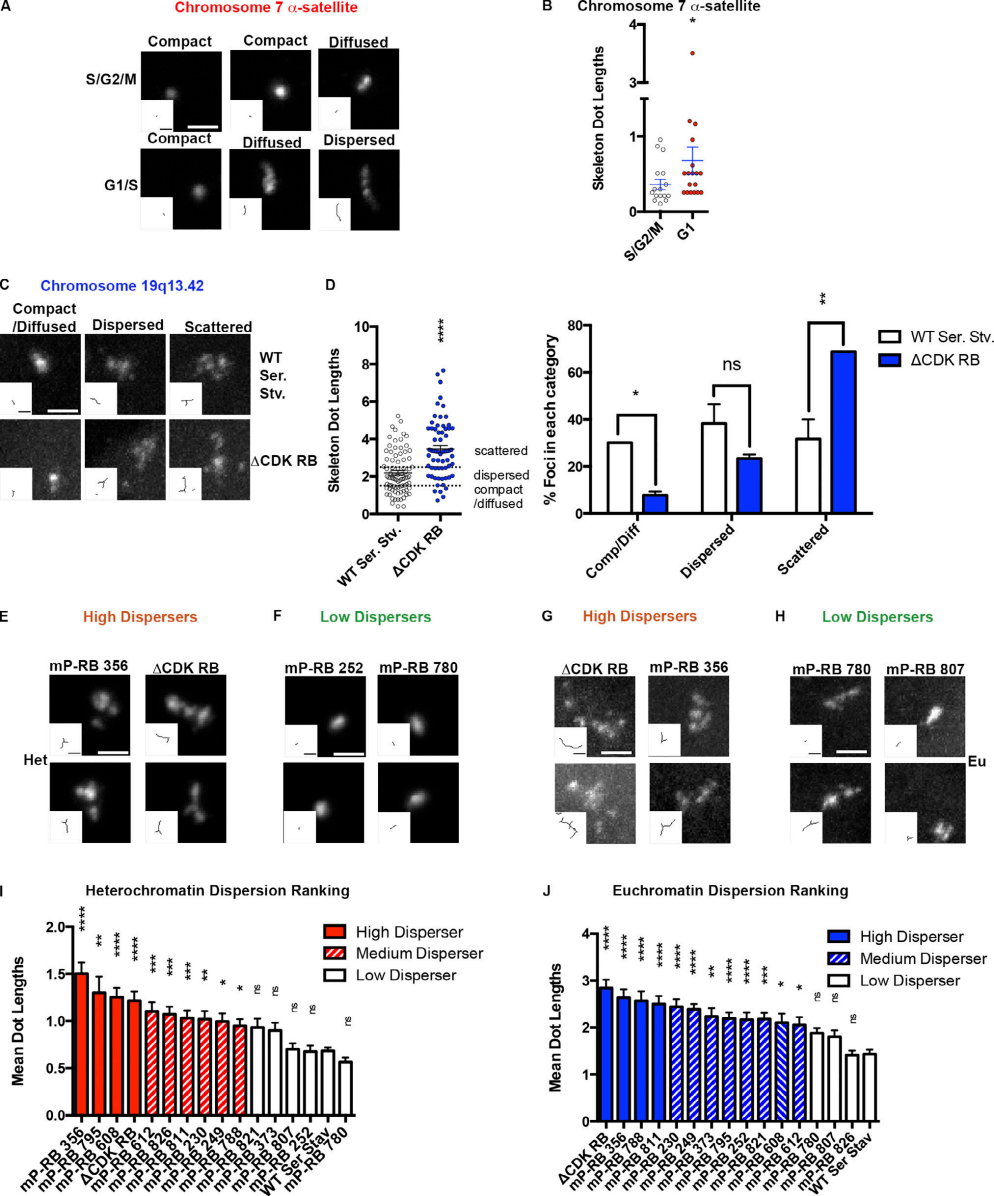
**Relationship between cell cycle stage, forms of active RB, and dispersion. (A)** The range of signals detected by the chromosome 7 α-satellite probe in Fucci RPE-1 cells. Low-level dispersion was observed in G1 cells but not in S/G2/M cells. **(B)** Scatter plots depicting distribution of skeleton dot length of signals and mean in S/G2/M and G1 cells. **(C and D)** The level of dispersion induced by ΔCDK-RB was greater than that seen when RPE cells were arrested by serum starvation (Ser. Stv.) for 72 h. The euchromatin probe 19q13.42 was used for FISH. **(C)** Representative images of FISH foci in each category. **(D)** Distribution of skeleton dot lengths, means, and percentages of foci in each category. Quantitation includes biological replicates, set up and performed on different days. **(E–J)** RB monophosphorylation forms differ in the ability to disperse euchromatin and heterochromatin regions. Mean dot lengths were used to classify RB monophosphorylation forms as high dispersers, medium dispersers, or low dispersers. **(E–H)** Examples of high dispersers and low dispersers detected with the heterochromatin probe (chromosome 7 α-satellite; E and F) or the 19q13.42 euchromatin probe (G and H). **(I and J)** Mean dot lengths obtained using heterochromatin and euchromatin regions, respectively. The rankings of the various forms of active RB are shown. Quantitation includes biological replicates, set up and performed on different days. Numbers of foci quantified for each sample (*n*) are as follows (in the order they appear on the bar graphs): B, *n* = 17, 18; D, *n* = 83, 64; I, *n* = 55, 48, 74, 61, 50, 75, 75, 76, 104, 60, 63, 56, 93, 85, 207, 115; J, *n* = 60, 84, 52, 113, 124, 144, 66, 120, 80, 106, 80, 56, 145, 69, 99, 111. Error bars are SEM. For B and D (left panel), asterisks denote significance from nonparametric two-tailed Mann–Whitney *U* test. For B (right panel), asterisks denote P values from multiple *t* tests (without correction for multiple comparisons). For I and J, asterisks denote P values from nonparametric Kruskal–Wallis test and Dunn’s multiple comparison test. ns, P > 0.05; *, P ≤ 0.05; **, P ≤ 0.01; ***, P ≤ 0.001; ****, P ≤ 0.0001. Scale bar (including insets) = 2 µm. Dashed lines indicate the cutoffs for defining the categories compact, diffused, and dispersed for chromosome 7 α-satellite and compact/diffused, dispersed, and scattered for 19q13.42 probe.

Since IR, palbociclib, and ΔCDK-RB expression all cause G1 arrest, it was plausible that chromatin organization might fluctuate during the cell cycle and that the measured effects might reflect a state that is prevalent during G1. To explore this, FUCCI-RPE1 cells (Sakaue-Sawano et al., 2008; Shenk and Ganem, 2016) were used to distinguish G1 cells from S/G2/M phase cells in an asynchronous cycling population. The mean skeleton dot length was higher in G1 cells than in S/G2/M cells (Fig. 5, A and B), but the effect was smaller than that seen following ΔCDK-RB expression or palbociclib treatment (Figs. 4 B and 3 E, right panel). When we compared cells arrested by serum starvation with ΔCDK-RB–arrested cells, we found that serum starvation gave less dispersion than ΔCDK-RB–arrested cells (Fig. 5, C and D), even though it caused a similar accumulation of G1 phase cells. (Fig. S2 A). The percentage of scattered foci, in particular, was much higher in ΔCDK-RB–expressing cells than in serum-starved cells (Fig. 5 D). These results are consistent with data showing that ΔCDK-RB–induced dispersion is time dependent (Fig. 3 G) and support the idea that dispersion occurs in G1 cells. However, these observations suggest that dispersion is an induced state rather than a feature of all G1 cells.

During the time course of IR-induced senescence in IMR-90 fibroblasts, we noted that dispersion of the FISH signal was not an immediate event. Western blots demonstrate that the kinetics and extent of RB dephosphorylation varies between sites (Fig. S1 B). Since RB can be monophosphorylated on any one of 14 CDK sites during G1 (Narasimha et al., 2014), and the monophosphorylated forms of RB (mP-RBs) have distinct properties (Sanidas et al., 2019), we examined the effects of each mP-RB on chromatin organization. We used a panel of isogenic RPE1 cell lines that were generated by reintroducing single sites of CDK phosphorylation into ΔCDK-RB, and by putting these constructs into the DOX-inducible RB replacement system (Sanidas et al., 2019; Fig. S2 A). The mP-RBs interact with different sets of proteins and have varied transcriptional outputs, but they all share the ability to inhibit E2F transcription activity and cause G1 arrest.

Although, expression of all mP-RBs increased G1 (Fig. S2, C and D), the mP-RBs dispersed FISH signals to very different degrees (Fig. 5, E–J; and Fig. S2, E–H). We grouped the range of mP-RB phenotypes into high, medium, or low dispersers (Fig. 5, E–J). The euchromatic and heterochromatic FISH probes gave similar but nonidentical rankings (Fig. 5, I and J). At one end of the spectrum, the degree of dispersion caused by mP-RB 356 was comparable to that of ΔCDK-RB with both euchromatic and heterochromatic probes. In contrast, mP-RB 780 and mP-RB 807 were almost completely unable to induce dispersion (Fig. 5, I and J; and Fig. S2, E–H). These mP-RBs gave a high percentage of compact foci and showed no significant increase in mean skeleton dot length compared with serum-starved WT cells. These data are concordant with evidence that RB phosphorylation at S780 persists in G1-arrested RPE1 cells upon serum starvation and contact inhibition (Sanidas et al., 2019), two conditions that induce G1 without causing extensive chromosome dispersion. The fact that mP-RB 780 promotes the organization of these regions into compact foci (Fig. S2, E–H) may explain why dispersion/scattering is not more extensive in cells arrested by serum starvation or contact inhibition. Since all mP-RBs suppress E2F-dependent transcription, it is clear that dispersion is not a simple consequence of E2F-mediated repression.

We infer that all of the regions examined can be organized into states that have different degrees of compaction/dispersion/scatter. In asynchronously dividing cells, these regions typically form compact foci during S/G2. A minor fraction of cells have a more diffuse organization during G1 or in G1-arrested cells. However, the expression of unphosphorylated RB (ΔCDK-RB), or specific mP-RBs, greatly increased the dispersion/scattering of these regions, effects that were more extensive and more prevalent than the changes seen in other G1 cells. Hence, the diffuse, dispersed, and scattered organization of these chromosomal regions induced by ΔCDK-RB is not a universal feature of G1 cells but increases under specific conditions and is driven by specific forms of RB.

ΔCDK-RB increases the skeleton dot lengths of both heterochromatin and euchromatin regions, and visually it enhances the punctation, branching, and length of both kinds of chromatin. Previous studies showed that euchromatin regions tend to contract during replicative senescence, whereas heterochromatin regions expand (Criscione et al., 2016). Thus, the effects of RB on chromatin are distinct from the full catalog of changes that occur during senescence, and RB-mediated changes are presumably just one component of the overall change in organization. We note that some mP-RB mutants differ in their ability to disperse euchromatin and heterochromatin regions (Fig. 5, I and J; and Fig. S2, E–H). For example, mP-RB 821, mP-RB 373, and mP-RB 252 were medium dispersers for euchromatin regions but low dispersers for heterochromatin regions. Such differences suggest that individual forms of RB may use different mechanisms to promote dispersion.

### ΔCDK-RB–induced reorganization requires histone deacetylase (HDAC) and topoisomerase activities and alters interactions between RB-bound loci

**Figure 6.**
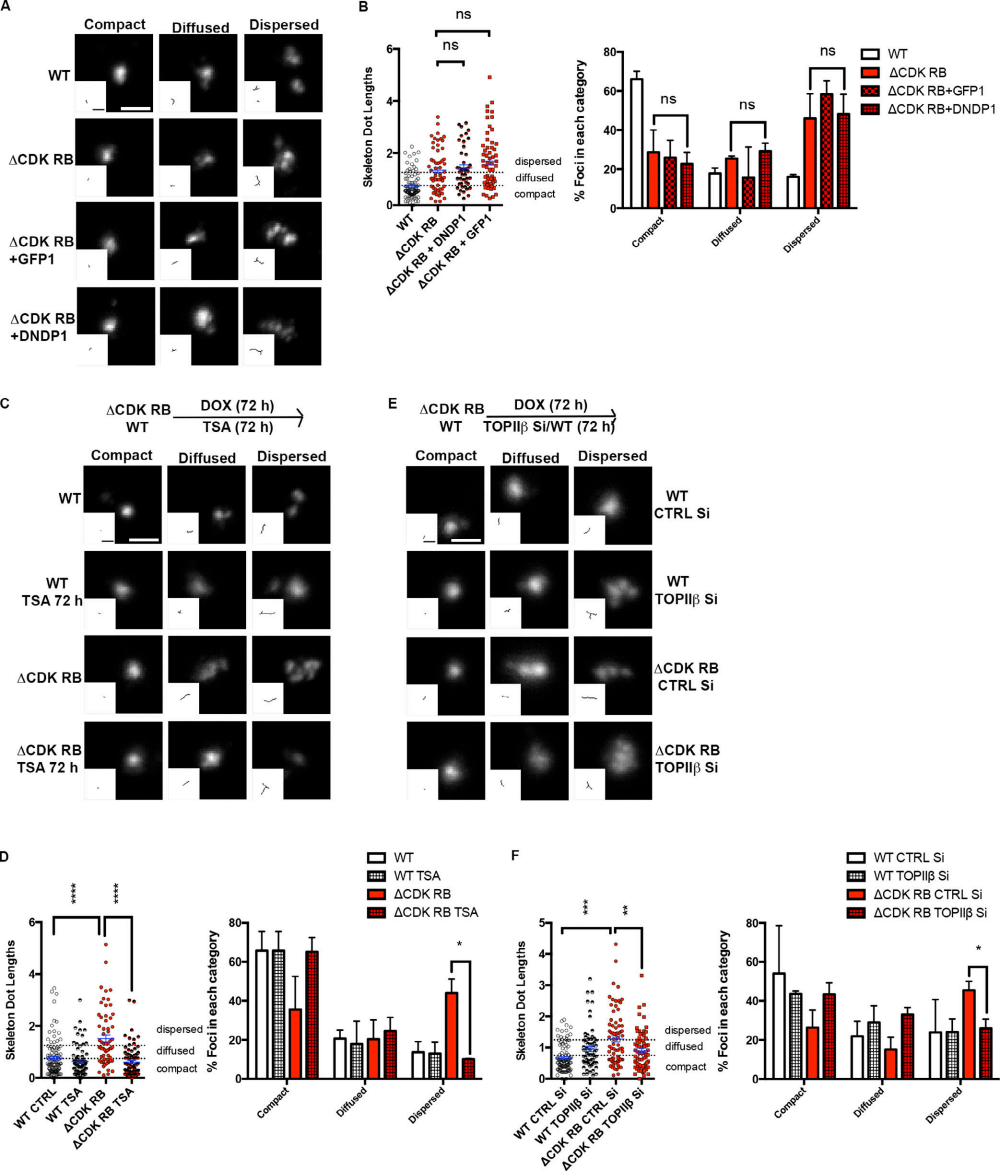
**ΔCDK-RB-induced dispersion is not dependent on the expression of DNDP1, but it requires HDAC activity and topoisomerase activity.** WT and ΔCDK-RB RPE cells were treated as indicated, and the FISH signal in chromosome 7 α-satellite region was analyzed. Representative FISH signals together with skeleton dot images are shown. The percentage of foci in each category is indicated. **(A and B)** Cells expressing DNDP1. DNDP1 did not alter dispersion induced by ΔCDK-RB. All quantitation is from two independent biological replicates, set up and performed on different days. **(C and D)** Cells treated with TSA for 72 h. Note that TSA treatment prevented the significant increase in dispersed foci and mean dot length seen in ΔCDK-RB cells. **(E and F)** Cells treated with TOPIIβ or control siRNAs (CTRL Si) for 72 h. TOPIIβ knockdown reduced the dispersion induced by ΔCDK-RB. Numbers of foci quantified for each sample (*n*) are as follows (in the order they appear on the bar graphs): B, *n* = 89, 59, 58, 45; D, *n* = 105, 75, 62, 89; F, *n* = 103, 66, 76, 68. Error bars are SEM. Nonparametric two-tailed Mann–Whitney *U* test was performed for pairs of samples indicated in B, D, and F (left panels), and asterisks denote P values. For B, D, and F (right panels), asterisks denote P values from nonparametric two-tailed multiple *t* tests (without correction for multiple comparisons). ns, P > 0.05; *, P ≤ 0.05; **, P ≤ 0.01; ***, P ≤ 0.001; ****, P ≤ 0.0001. Scale bar (including insets) = 2 µm. Dashed lines indicate the cutoffs for defining the categories compact, diffused, and dispersed for chromosome 7 α-satellite.

The four regions examined by FISH lack any well-studied E2F-regulated cell cycle genes or components of the RB loss gene signature (Markey et al., 2007; Müller et al., 2001), but the two euchromatin regions contain plenty of RB- and E2F1-bound loci (Fig. 4 I). As an additional test of a potential role for E2F in ΔCDK-RB–induced dispersion, we examined the effects of expressing a dominant-negative DP1 (DNDP1; Wu et al., 1996; Fig. S3 A), which efficiently reduced E2F1 and RB binding to a set of E2F-regulated promoters (Fig. S3 B). The consequences of eliminating the DNA binding activity of E2F are known to depend on cellular context: when a cell population is actively repressing E2F-dependent transcription, then general inhibition of E2F relieves this arrest (He et al., 2000; Zhang et al., 1999). Consistent with this, DNDP1 was able to suppress the strong effects of ΔCDK-RB on cell cycle distribution (Fig. S3 C), yet DNDP1 failed to prevent ΔCDK-RB–induced dispersion (Fig. 6, A and B; and Fig. S3 D). This provides further support for the notion that the mechanisms allowing ΔCDK-RB to promote dispersion extend beyond the canonical roles of RB or E2F-1 at cell cycle–regulated promoters.

In addition to E2F, RB interacts with many transcription factors and recruits various activities to chromatin (Dick and Rubin, 2013; Morris and Dyson, 2001). We used chemical inhibitors to ask whether some of the known RB-associated activities were important for ΔCDK-RB–induced dispersion. EZH2 and HDACs have both been reported to promote transcriptional repression and to facilitate heterochromatin formation by RB (Ishak et al., 2016; Montoya-Durango et al., 2016). While EZH2 inhibition failed to suppress chromosome dispersion (Fig. S3, E and F) in ΔCDK-RB–expressing cells, dispersion was strongly suppressed by trichostatin A (TSA), an inhibitor of HDAC1 and HDAC2 (Fig. 6, C and D; and Fig. S3 G).

**Figure S4.**
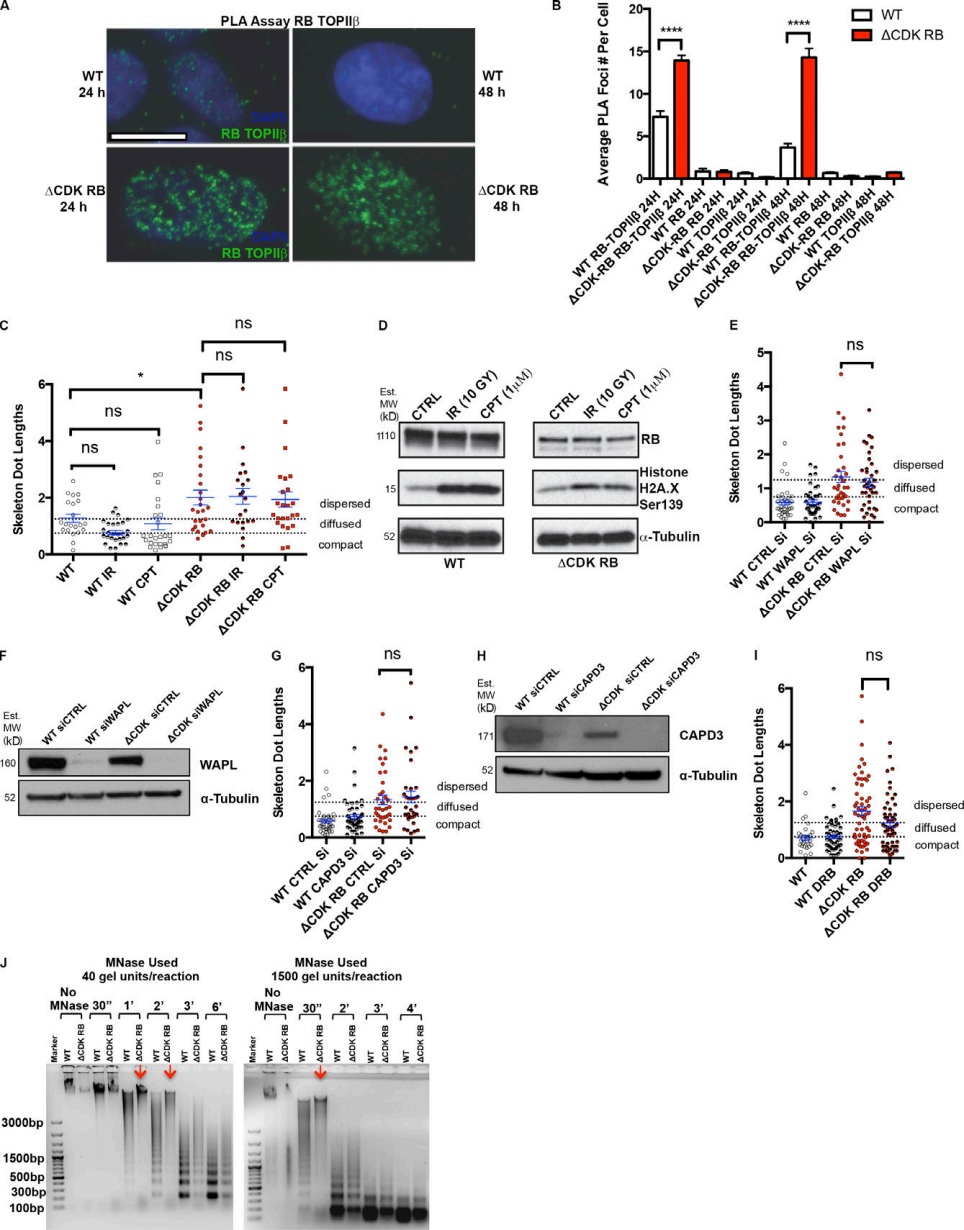
**Investigation of the role of DNA damage, WAPL, and CAPD3 in RB-mediated dispersion. RB activation increases chromatin resistance to MNase digestion. (A)** PLA images for WT and ΔCDK-RB after 24 and 48 h of DOX induction. Just 24 h of ΔCDK-RB expression leads to higher RB-TOPIIβ PLA interaction foci, when compared with WT cells. A similar increase was observed after 48 h of ΔCDK-RB induction. Images show G1 cells. **(B)** Average numbers of RB-TOPIIβ PLA foci per G1 cell were higher for ΔCDK-RB after 24 and 48 h of DOX induction, when compared with WT. Negative controls (using only RB or TOPIIβ antibody for PLA assay) showed low average numbers of PLA foci in both WT and ΔCDK-RB cells, implying that there was very little background signal. Numbers of foci quantified for each sample (*n*) are as follows: *n* = 95 (WT, RB-TOPIIβ 24 h), 214 (ΔCDK-RB, RB-TOPIIβ 24 h), 29 (WT, RB 24 h), 83 (ΔCDK-RB, RB 24 h), 70 (WT, TOPIIβ 24 h), 74 (ΔCDK-RB, TOPIIβ 24 h), 80 (WT, RB-TOPIIβ 48 h), 162 (ΔCDK-RB, RB-TOPIIβ 48 h), 69 (WT, RB 48 h), 110 (ΔCDK-RB, RB 48 h), 70 (WT, TOPIIβ 48 h), 130 (ΔCDK-RB, TOPIIβ 48 h). Error bars show SEM. Nonparametric two-tailed Mann–Whitney *U* test was performed for pairs of samples indicated on the graph, and asterisks denote P values. **(C)** Mean skeleton dot lengths (chromosome 7 α-satellite probe) after WT and ΔCDK-RB RPE cells were treated with IR or camptothecin (CPT) to induce DNA damage. Numbers of foci quantified for each sample (*n*) are as follows (in the order they appear on the bar graphs): *n* = 21, 28, 25, 26, 21, 24. Error bars are SEM. One-way ANOVA and Holm–Sidak multiple comparison tests were performed, and asterisks denote P values. **(D)** Western blot analysis of cells in C show that IR and CPT induced H2A.X Ser139 phosphorylation in both WT and ΔCDK-RB–expressing cells. Note that IR- or CPT-induced DNA damage does not affect the ΔCDK-RB–induced chromatin dispersion. MW, molecular weight. **(E, G, and I)** Mean skeleton dot lengths (chromosome 7 α-satellite probe) after WT and ΔCDK-RB RPE cells were treated with control and CAPD3 siRNAs (E), control and WAPL siRNAs (G), or DMSO and DRB (I). Note that none of these treatments significantly modified dispersion levels in either WT or ΔCDK-RB–expressing RPE cells. Numbers of foci quantified for each sample (*n*) are as follows (in the order they appear on the bar graphs): E, *n* = 40, 41, 36, 39; G, *n* = 40, 47, 36, 34; I, *n* = 29, 51, 58, 53. Error bars are SEM. For E, G, and I, nonparametric two-tailed Mann–Whitney *U* test was performed for pairs of samples indicated on the graphs, and asterisks denote P values. ns, P > 0.05; *, P ≤ 0.05; ****, P ≤ 0.0001. **(F and H)** Western blots for WT and ΔCDK-RB cells treated with control and WAPL siRNAs (F) and control and CAPD3 siRNAs (H). **(J)** MNase digestion profile of WT and ΔCDK-RB samples. Left: MNase digestion profile for WT and ΔCDK-RB nuclei treated with 40 gel units of MNase per reaction. Right: MNase digestion profile for WT and ΔCDK-RB cells treated with 1,500 units of MNase per reaction. All other conditions were the same. Note: WT samples resolve into a ladder-like typical MNase digestion pattern as early as 30 s at higher MNase concentrations and 1 min at lower concentrations. The ΔCDK-RB sample does not digest into a ladder-like pattern at 30 s at higher MNase concentrations and at even 1 min at lower concentrations (red arrows). A clear MNase digestion ladder is seen at 3 min (left gel) and 2 min (right gel) in the ΔCDK-RB samples, much later than the laddering in WT cells. 1 µg DNA was loaded per well. DNA samples were run on 0.8% agarose gel. Dashed lines indicate the cutoffs for defining the categories compact, diffused, and dispersed for chromosome 7 α-satellite. Source data are available for this figure: SourceData FS4.

RB has also been found to associate with type II topoisomerases (TOPIIs) and to facilitate the processing and repair of TOPII-induced double-strand breaks during alterations of DNA topologic states (Goodrich, 2006; Xiao and Goodrich, 2005). Interestingly, the TOPII inhibitor etoposide (but not the TOPI inhibitor, topotecan; not depicted) inhibited dispersion in ΔCDK-RB cells. When we tested siRNAs specific for TOPIIα (Fig. S3 H) or TOPIIβ (Fig. S3 I), we found that RB-induced dispersion was particularly dependent on TOPIIβ activity (Fig. 6, E and F; and Fig. S3 J). Consistent with this, proximity ligation assays (PLAs) showed enhanced colocalization of RB with TOPIIβ 24 and 48 h after induction of ΔCDK-RB expression, when compared with WT cells (Fig. S4, A and B). We induced DNA damage in WT cells or ΔCDK-RB using γ-IR or camptothecin, to test whether the consequences of HDAC inhibition or TOPIIβ depletion on chromatin dispersion were potentially attributable to DNA damage responses. Although both treatments caused similar DNA damage in both cell lines, they neither induced nor suppressed dispersion (Fig. S4, C and D), indicating that dispersion is uncoupled from DNA damage.

The effects of TSA, etoposide, and TOPIIβ knockdown are notable when contrasted with treatments that did not affect dispersion. RB has been linked to chromosome architecture and genome stability through its interactions with Condensin II complexes or through effects on Cohesin loading (Kim et al., 2021; Longworth et al., 2008; Manning et al., 2014). We used siRNA to deplete the Cohesin remover WAPL or the Condensin II component NCAPD3. In both cases, the depletion did not change the dispersion phenotype of ΔCDK-RB–expressing cells (Fig. S4, E–H), although we acknowledge that a negative result in these types of knockdown experiments must be interpreted cautiously. Furthermore, we observed no specific effects with VE-821 (which inhibits ATR-mediated DNA repair pathways; data not shown) or 5,6-dichloro-1-β-d-ribofuranosylbenzimidazole (DRB), a general inhibitor of transcription (Fig. S4 I).

**Figure 7.**
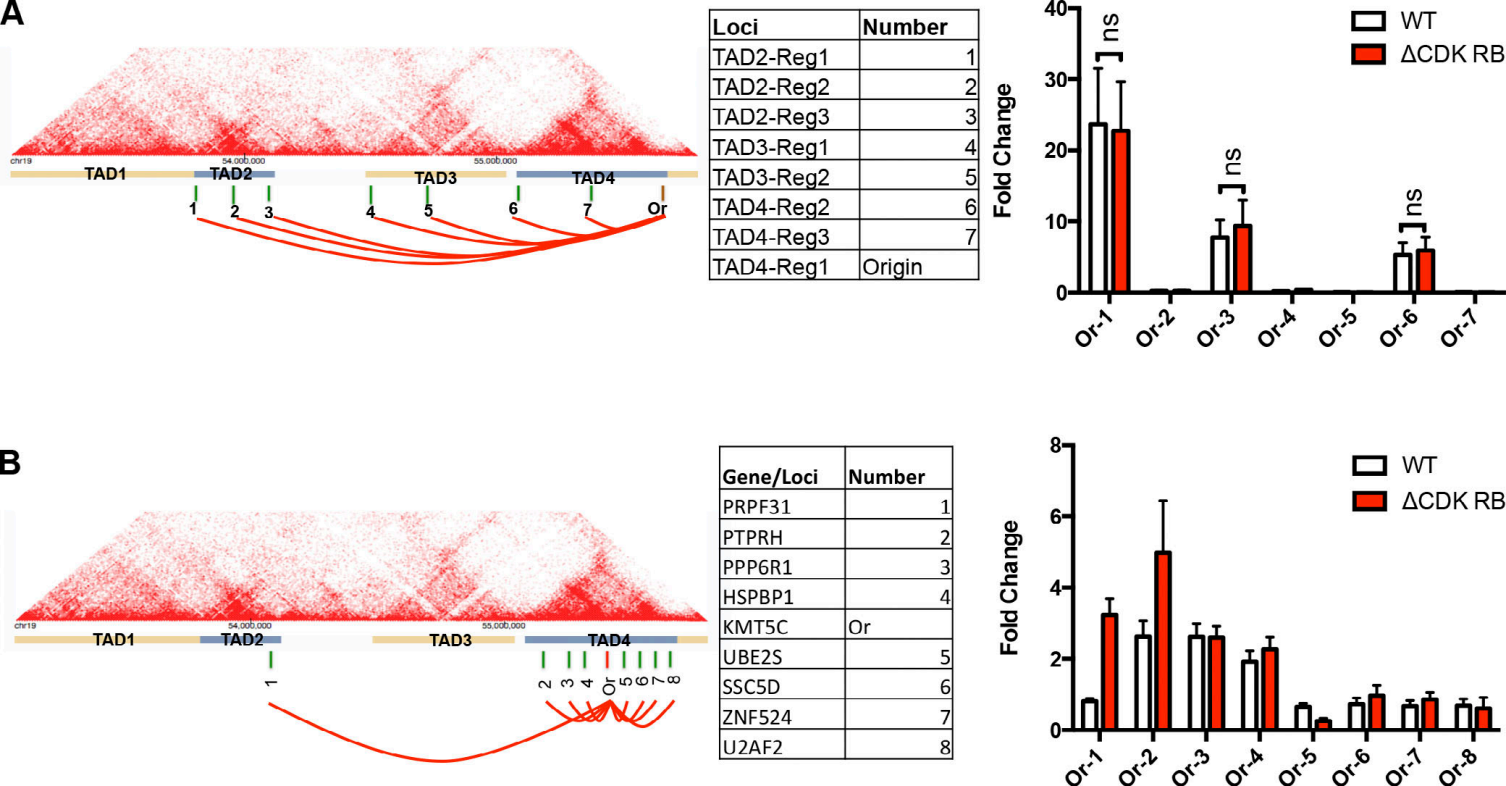
**ΔCDK-RB causes a reorganization of interactions between RB-bound loci within 19q13.42. (A and B)** Interaction map of 19q13.42 from Wang et al. (2018) showing the four TADs and position of primers. **(A)** 3C was used to examine inter-TAD interactions between sites at TAD boundaries. Sites tested are indicated as green bars on the map, the origin is indicated by a brown bar, and these sites are listed in the table. Interactions between sites are shown in the right panel, with graph showing the fold-change (enrichment over TAD4-reg1 [origin] self-ligation product signal; see Materials and methods for details) detected in cells expressing either WT RB or ΔCDK-RB. **(B)** 3C was used to detect interactions between sites near to RB-bound loci. Green bars on map indicate position of the loci tested, and origin for 3C is indicated by red bar. These sites are listed in the table. Interactions (fold-change calculated as enrichment over KMT5C [origin] self-ligation product signal) were detected between the origin and multiple sites; of these, three showed significant differences (PRPF31, PTPRH, and UBE2S) between cells expressing WT RB or ΔCDK-RB. Graphs show mean fold-change of three independent biological replicates, set-up and performed on different days. Error bars are SEM. Nonparametric two-tailed multiple *t* tests (with correction for multiple comparisons) was used to calculate P values for PRPF31 (P = 0.0004), PTPRH (P = 0.15), and UBE2S (P = 0.008).

To examine dispersion in more detail, we selected one of the euchromatin regions (19q13.42) and used chromatin conformation capture (3C), a proximity-based ligation assay, to look for effects of ΔCDK-RB on intrachromosomal interactions. An interaction map of the region (Wang et al., 2018) was used to design probes (Fig. 7, A and B, left panels). We first examined interactions between sites at boundaries of topologically associating domains (TADs). Strong interactions were detected between sequences near to TAD boundaries, the strongest being between TAD4 and TAD2 boundaries, but no significant differences in these interactions were detected between ΔCDK-RB and WT cells (Fig. 7 A), suggesting that the TAD boundaries are largely intact. This observation is in agreement with Hi-C data for oncogene-induced senescence and replicative senescence (Chandra et al., 2015; Criscione et al., 2016; Zirkel et al., 2018). Next, RB ChIP-seq data were used to design 3C probes targeting RB-binding sites in the loops between the TAD boundaries. These probes revealed multiple interactions between RB-bound regions. Interestingly, some of the interactions changed significantly in ΔCDK-RB–expressing cells relative to cells expressing WT RB, while others remained unchanged (Fig. 7 B). In particular, we found interactions between three pairs of RB-bound regions (KMT5C and UBE2S, KMT5C and PTPRH, and KMT5C and PRPF31) that were altered by ΔCDK-RB in three independent experimental replicates.

Collectively, these molecular studies show that the activation of RB changes the patterns of interactions between some RB-bound loci. 3C assays are focused in nature, and a more complete assessment of chromatin interactions will be required to identify the global effects of RB on TAD boundaries and between loci bound (or unbound) by RB. Nevertheless, these results confirm that ΔCDK-RB causes changes in chromatin organization. ΔCDK-RB–induced dispersion does not appear to be driven by classic E2F targets, but it requires HDACs and topoisomerases, two chromatin regulatory activities previously shown to associate with RB.

### The changes in nuclear organization induced by ΔCDK-RB are widespread

**Figure 8.**
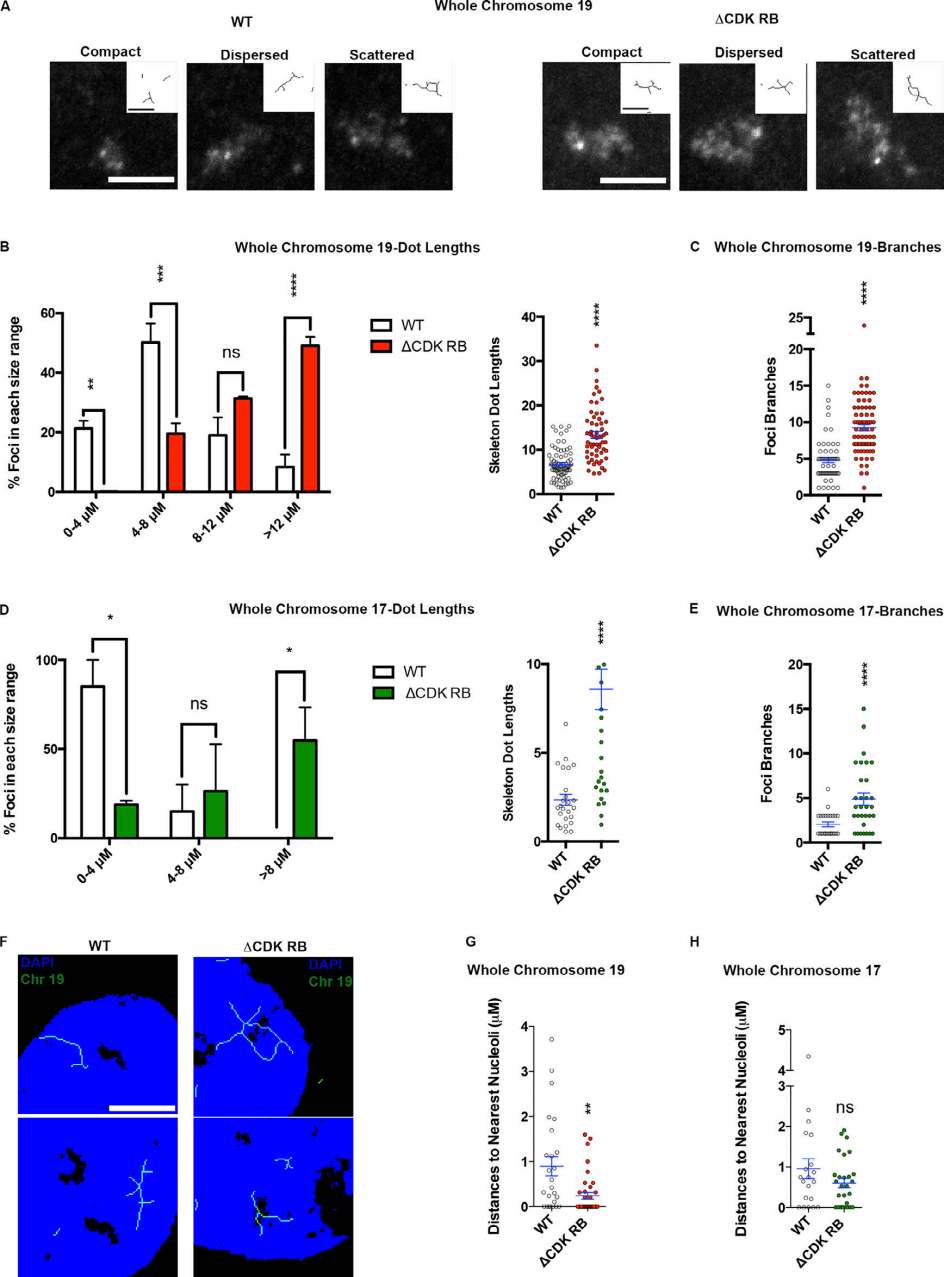
**ΔCDK-RB expression alters the organization and position of whole chromosomes.** Cells were induced to express WT RB or ΔCDK-RB and probed by FISH with probes to chromosome 19 (A–C, F, and G) or chromosome 17 (D, E, and H). **(A)** The range of signals detected by chromosome 19 probe in cells induced to express WT RB (left) or ΔCDK-RB (right). ΔCDK-RB induces the whole chromosome 19 foci scattering. **(B and C)** Skeleton dot length was used to place the FISH signals into bins. Graphs show the percentage of foci within each size range (B, left panel), the distribution of skeleton dot lengths and their mean (B, right panel), and the mean number of branches per foci (C). Each parameter increased significantly after ΔCDK-RB expression. **(D and E)** Similar ΔCDK-RB–induced changes were observed using FISH probe to chromosome 17 foci. **(F)** Chromosome 19 localizes to nucleolar periphery in ΔCDK-RB–expressing cells. Representative images are shown. **(G and H)** Quantitation of the shortest distance from the nearest nucleolar signal to chromosome 19 (G) or chromosome 17 (H). Quantitation is from two independent biological replicates, set up and performed on different days. Numbers of foci quantified for each sample (*n*) are as follows (in the order they appear on the bar graphs): B, *n* = 77, 60; C, *n* = 60, 73; D and E, *n* = 25, 31; G, *n* = 25, 38; H, *n* = 20, 28. Error bars are SEM. For B, D (right panels), C, and E, nonparametric two-tailed Mann–Whitney *U* test was performed, and asterisks denote P values. For B and D (left panels), asterisks denote P values from nonparametric two-tailed multiple *t* tests (without correction for multiple comparisons). ns, P > 0.05; *, P ≤ 0.05; **, P ≤ 0.01; ***, P ≤ 0.001; ****, P ≤ 0.0001. Scale bar (including insets) = 5 µm.

To ask whether the nuclear changes extend beyond the four regions examined by FISH and 3C, the effects of ΔCDK-RB were assessed using whole-chromosome probes. As expected, probes for chromosomes 17 and 19 gave FISH signals that were more extensive than the regional probes, yet they clearly scattered further following ΔCDK-RB expression (Fig. 8, A–E). Quantification showed a two- to threefold increase in mean skeleton dot length for both chromosome 19 (Fig. 8 B, right panel) and chromosome 17 (Fig. 8 D, right panel) and a wider distribution of skeleton dot lengths. When dot lengths were grouped into different bins, it was also evident that ΔCDK-RB expression increased the percentage of chromosomes with the greatest degree of dispersion/scatter (Fig. 8, B and D, left panels).

**Figure S5.**
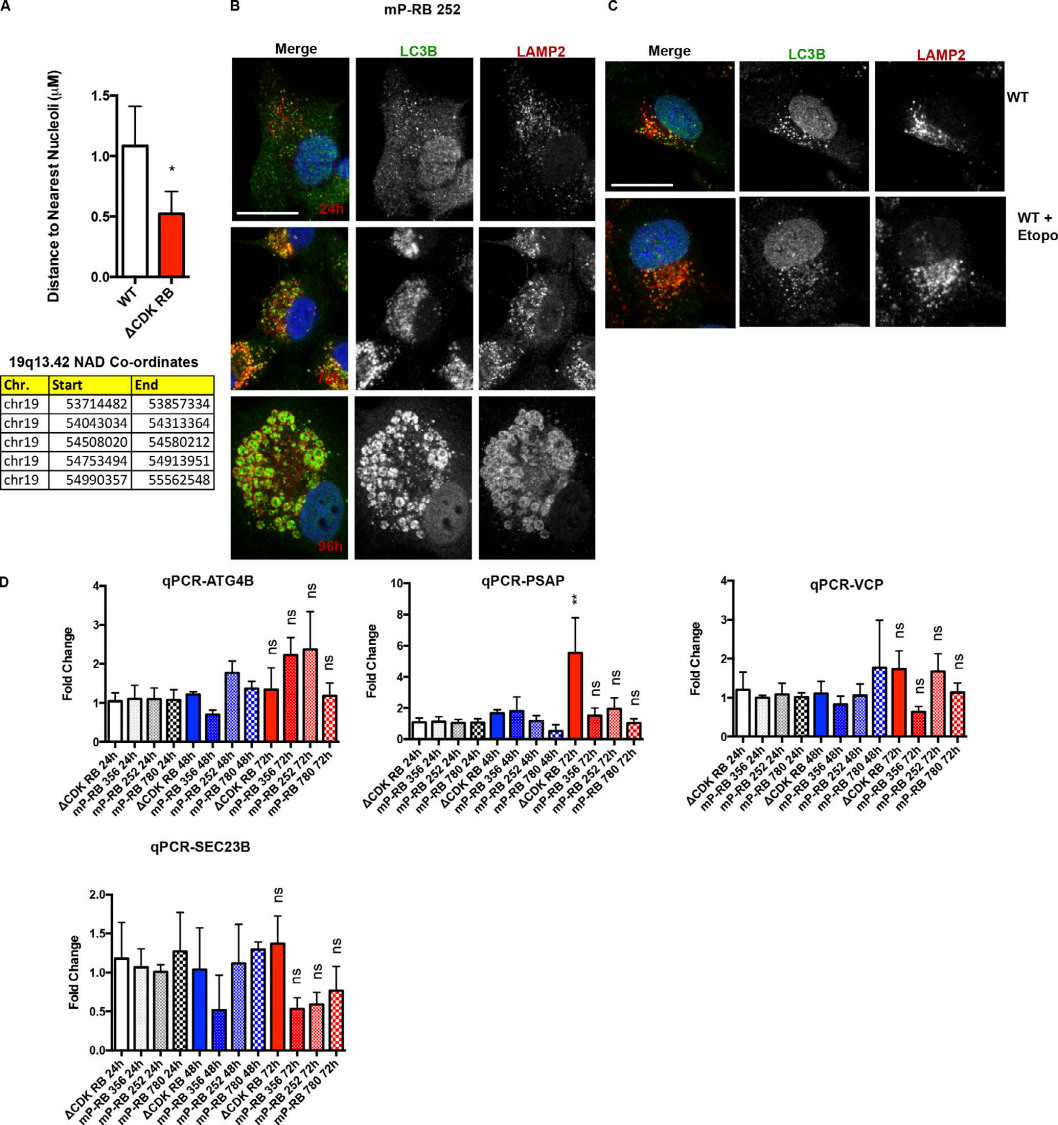
**19q13.42 locus positions closer to nucleoli in ∆CDK-RB cells. Autophagy response in mPRB-252 and WT-RB expressing cells. (A)** Quantitation of the shortest distance between the nearest nucleoli and 19q13.42 FISH signal in WT and ΔCDK-RB cells and coordinates of nucleolar associated domains (NADs) mapped to the region (Dillinger et al., 2017). Numbers of foci quantified for each sample (*n*) are as follows (in the order they appear on the bar graphs): *n* = 11, 23. Nonparametric two-tailed Mann–Whitney *U* test was performed for pairs of sample on the graph. Chr., chromosome. **(B)** Representative LC3B and LAMP2 IF staining images for mP-RB 252 cells 24, 72, and 96 h after DOX induction. 48-, 72-, and 96-h time point cells were treated with 100 nM bafilomycin A1 for 24 h before fixation. 24-h time point cells were treated with 100 nM bafilomycin A1 for 3 h before fixation. **(C)** Representative LC3B and LAMP2 IF images for WT induced with DOX for 72 h and treated with 0.5 µM etoposide for the final 24 h. Cells were treated with bafilomycin A1 for 24 h before IF fixation. **(D)** qPCR of four genes (*ATG4B*, *PSAP*, *VCP*, and *SEC23B*), at 24-, 48-, and 72-h time points of DOX induction for ΔCDK-RB, mP-RB 356, mP-RB 252, and mP-RB 780 cell lines. Graphs show fold-changes (enrichment over 24-h time point samples) for the four genes. For D, asterisks denote P values (comparison with 24-h time point samples) from one-way ANOVA and Newman–Keuls multiple comparison test. ns, P > 0.05; **, P ≤ 0.01.

A second feature of the chromosome 19 (Fig. 8 C) and 17 (Fig. 8 E) FISH signals was that they branched to a higher degree in ΔCDK-RB–expressing cells, relative to WT, an effect that likely reflects the enhanced scattering and punctation of foci. In addition, we noticed that chromosome 19 localized near to nucleolar structures in a large percentage of ΔCDK-RB–expressing cells (Fig. 8 F). Quantification of the nearest nucleolar distance for chromosome 19 confirmed that the average distance was significantly lower in ΔCDK-RB–expressing cells compared with WT (Fig. 8 G). This effect was not seen with the chromosome 17 probe (Fig. 8 H). Chromosome 19 has been reported to contain unique sequence features and epigenetic signatures (Blahnik et al., 2011; Harris et al., 2020), and these may affect some of these microscopic observations. To further explore the changes in nucleolar positioning in ΔCDK-RB–expressing cells, we quantified the nearest nucleolar distance for the euchromatic FISH probe 19q13.42, which contains four nucleolar-associated domains (Fig. S5 A). Similar to the effect seen with the whole chromosome 19 probe, in ΔCDK-RB–expressing cells, 19q13.42 positioned closer to the nucleolus, when compared with WT (Fig. S5 A). Together, these data suggest that ΔCDK-RB alters the relative position of either chromosome 19 or nucleolar structures, or both.

These results show that RB does not simply cause reorganization of a few small regions. Instead RB has extensive effects on nuclear organization that are evident across whole chromosomes and even impact chromosome positioning within the nucleus. Using a complementary biochemical method to look for global changes, we treated chromatin with micrococcal nuclease (MNase) and found that ΔCDK-RB expression increased the resistance of chromatin to digestion (Fig. S4 J). Genomewide chromatin accessibility assays will be required to understand how these changes affect individual loci. Nevertheless, the bulk changes support the conclusion that active RB does not simply affect the organization of a few small locations, but instead causes extensive changes in chromatin and chromosomal organization.

### Dispersion connects with an autophagy program

**Figure 9.**
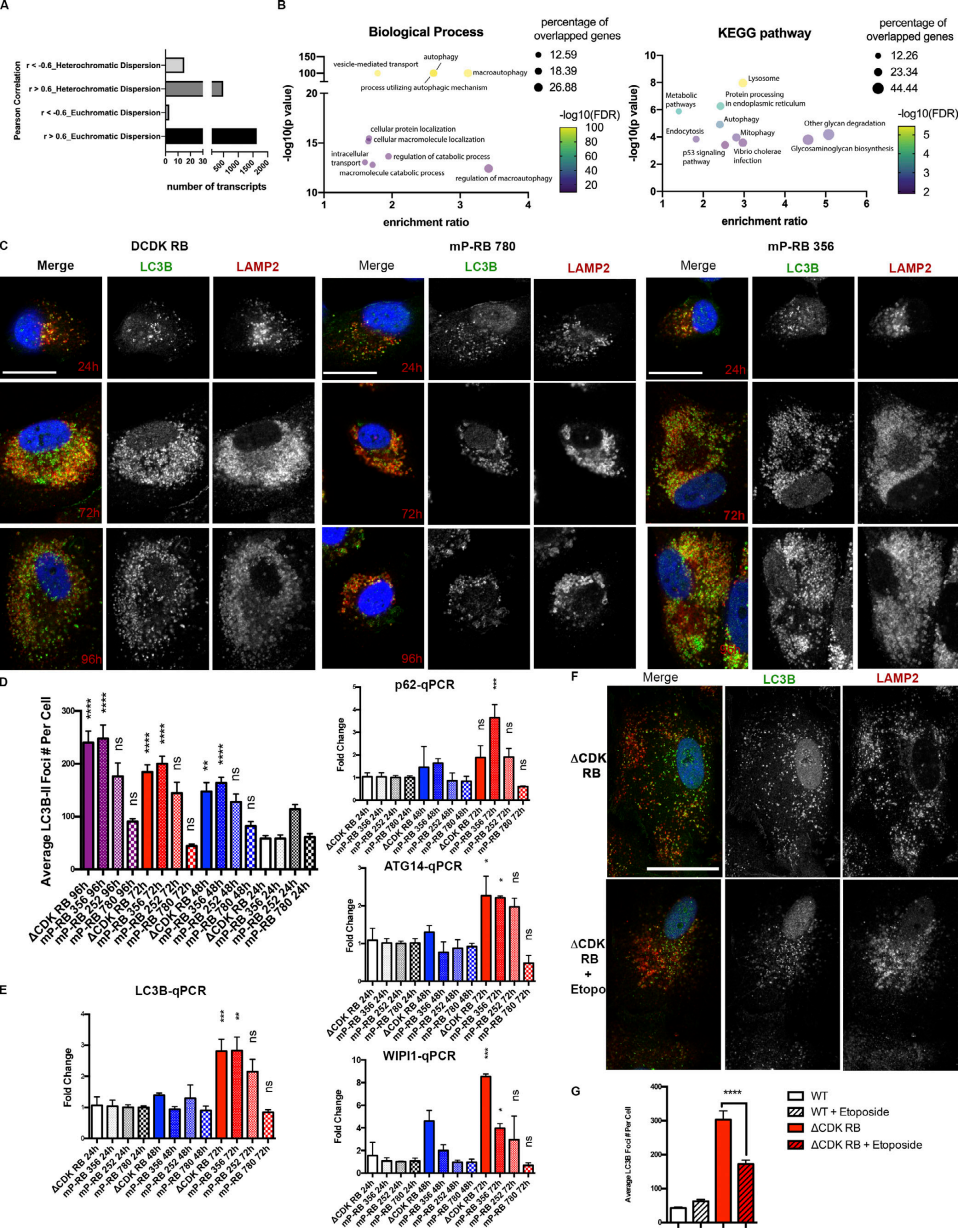
**RB-induced dispersion is associated with increased expression of autophagy genes. (A)** Number of transcripts that positively (*r* > 0.6) or negatively (*r* < −0.6) correlate with heterochromatin and euchromatin dispersion rankings. **(B)** Left: Biological pathway analysis for correlated genes. Right: Kyoto Encyclopedia of Genes and Genomes (KEGG) pathway analysis for correlated genes. FDR, false discovery rate. **(C)** Representative LC3B and LAMP2 IF staining images for ΔCDK-RB, mP-RB 356, and mP-RB 780 cells 24, 72, and 96 h after DOX induction. Scale bar = 25 µm. **(D)** Quantification of LC3B-II average puncta per cell for ΔCDK-RB, mP-RB 356, mP-RB 252, and mP-RB 780 24, 48, 72, and 96 h after DOX induction. 48-, 72-, and 96-h time point cells were treated with 100 nM bafilomycin A1 for 24 h before fixation. 24-h time point cells were treated with 100 nM bafilomycin A1 for 3 h before fixation. **(E)** qPCR of four genes (LC3B, p62, *ATG14*, and *WIPI1*), at 24-, 48-, and 72-h time points of DOX induction for ΔCDK-RB, mP-RB 356, mP-RB 252, and mP-RB 780 cell lines. Graphs show fold-changes (enrichment over 24-h time point samples) for the four genes. Three technical replicates per sample were used to calculate fold-changes. **(F)** Representative LC3B and LAMP2 IF images for WT and ΔCDK-RB induced with DOX for 72 h and treated with 0.5 µM etoposide for the final 24 h. Cells were treated with bafilomycin A1 for 24 h before IF fixation. **(G)** Quantification of LC3B-II average puncta per cell for WT and ΔCDK-RB untreated and treated with 0.5 µM etoposide for 24 h. We observed that etoposide suppresses LC3-II punta in ΔCDK-RB cells. Scale bar = 50 µm. Numbers of foci quantified for each sample (*n*) are as follows (in the order they appear on the bar graphs): D, *n* = 18, 22, 27, 18, 30, 18, 28, 20, 20, 28, 28, 20, 24, 20, 26, 24; G, *n* = 17, 22, 15, 27. For D, asterisks denote P values from nonparametric Kruskal–Wallis test and Dunn’s multiple comparison test (each sample was compared with its 24-h time point). Error bars are SEM. ns, P > 0.05; *, P ≤ 0.05; **, P ≤ 0.01; ***, P ≤ 0.001; ****, P ≤ 0.0001.

The action of RB is often tracked using transcription signatures. To ask whether specific changes in gene expression are associated with chromatin reorganization, we took advantage of the discovery that individual mP-RBs induce dispersion to different degrees. Pearson correlation analysis was performed between the average skeleton dot length rankings of euchromatin region 19q13.42 and reads-per-million expression data from cells expressing ΔCDK-RB or any of the 14 mP-RB mutant alleles, 48 h after DOX treatment (Sanidas et al., 2019). Approximately 1,627 transcripts had a positive correlation with the dispersion phenotype (*r* > 0.6). In contrast, only three transcripts showed a negative correlation (*r* < −0.6; Fig. 9 A). Gene ontology (GO) analysis of transcripts that correlated positively with dispersion revealed a strong and significant enrichment of genes involved in autophagy (GO 0006914 and GO 0016236 for biological processes and hsa04140 and hsa04142 for Kyoto Encyclopedia of Genes and Genomes pathways; Fig. 9 B; see also Table S1). Importantly, classic E2F-target or senescence-associated genes were not enriched by this analysis, indicating again that dispersion is not associated with the canonical function of RB. Changes in expression of 97 autophagy genes correlated positively with chromatin dispersion (*r* > 0.6), including genes that are required for autophagosome formation and maturation (Table S2). Of note, analysis of RB ChIP-seq data indicates that 78 of these genes have RB-bound promoters or enhancers, suggesting that their expression can be directly regulated by RB.

Despite the significance of this correlation, the absolute changes in transcript levels were relatively small. To experimentally test the inference that RB-induced dispersion is associated with autophagy, we followed two approaches. First, we asked whether chromatin dispersion is linked to changes in autophagy flux. LC3B staining was performed to detect autophagosomes at various time points following induction of ΔCDK-RB and mP-RB expression. To ensure that LC3B signals/puncta are autophagosome associated (LC3B-II) and the increase in their levels was not due just to defects in autophagosome clearance, we costained for LAMP2 (a lysosomal marker) as well. We compared ΔCDK-RB and mP-RB 356, two forms of RB that strongly induce dispersion with mP-RB 780 (largely defective for dispersion) and mP-RB 252 (an intermediate euchromatin dispersion phenotype). Cells were stained after treatment with or without bafilomycin, a vacuolar H+/ATPase inhibitor that prevents lysosomal acidification and allows autophagosomes to accumulate, making it possible to quantify autophagy levels. Time course samples (24, 48, 72, and 96 h after DOX induction) showed a progressive increase in LAMP2-colocalized LC3B-II puncta in cells expressing ΔCDK-RB and mP-RB 356 (which caused strong dispersion; Fig. 9, C and D). All the lines had a similar number of average LC3B-II puncta after 24-h DOX induction. An increase in the number of LC3B-II puncta was first apparent for ΔCDK-RB and mP-RB 356 at 48 h and increased progressively to 96 h. Temporally, this LC3B-II response coincided with dispersion onset and progression. mP-RB 780 (low dispersion) did not show any LC3B-II puncta induction over the time course. Expression of mP-RB 252 (medium dispersion) gave an intermediate effect and a modest (but statistically insignificant) increase in LC3B-II puncta with time (Fig. 9, C and D). Thus, across this panel of alleles, both the extent of LC3B-II staining and the timing of its appearance correlated well with dispersion. Basal levels of LAMP2-associated LC3B-II (without bafilomycin treatment) did not show differences for the various dispersers over the time course, suggesting that the clearance of autophagosomes is not defective in these cells.

Second, we measured the transcript levels of specific genes required for autophagosome formation in cells expressing forms of RB. A subset of these (LC3B, p62, *ATG14*, and *WIPI1*) increased expression specifically in cells presenting high to medium chromatin dispersion (ΔCDK-RB, mP-RB 356, and mP-RB 252), in a time-dependent manner, upon RB activation (Fig. 9 E). This pattern of increased expression was not seen with all autophagy-related genes. It was not seen, for example, with *ATG4B*, *PSAP*, *VCP*, or *SEC23B*, genes that had readily detectable levels of RNA in RPE1 cells (Fig. S5 D). Hence the increase in LC3-II staining appears to be driven by increased levels of a subset of autophagy genes.

To test the idea that RB-induced dispersion acts upstream of changes in autophagy gene expression, we examined LC3B-II in cells induced to express ΔCDK-RB and then treated with etoposide (an inhibitor of TOPIIβ) to suppress dispersion. The number of LC3B-II puncta was reduced significantly by etoposide treatment (Fig. 9, F and G). Conversely, inhibition of autophagy using bafilomycin treatment of ΔCDK-RB–expressing cells had no effect on ΔCDK-RB–induced dispersion (data not shown). Although the inhibition of the RB-induced dispersion by a TOPIIβ inhibitor reduced autophagosome formation, the expression signatures linking RB and autophagy are correlative data. The underlying mechanism could be direct or indirect and needs further exploration.

In summary, these results show that chromatin dispersion induced by active RB was associated with increased autophagy flux and increased expression of a panel of autophagy-related genes. RB is typically linked to the regulation of cell cycle genes, and this association is notable because it is an unusual signature for an RB-induced phenotype.

## Discussion

In this study, we have visualized changes in chromosome and nuclear organization driven by the activation of RB. For years, we and others have speculated that RB might control aspects of chromosome structure, but to date, it had not been possible to observe RB performing this role. Here, using FISH probes, we show that expression of unphosphorylated RB (and specific mP-RBs) causes changes in the organization of euchromatin and heterochromatin domains that are visible under the microscope.

Between 48 and 72 h after the induction of active RB, regions of heterochromatin changed in appearance, moving from a compact focus into an undefined shape with multiple domains, fingers, and clusters of foci. In a similar time frame, regions of euchromatin that already had a visibly noncompact organization became more diffuse, with increased numbers of foci that became spaced farther apart. These changes, which we call RB-induced dispersion, were apparent with multiple probes, were seen across whole chromosomes, and when quantified, caused a two- to threefold increase in the mean skeleton dot length of FISH signals. Accompanying these changes, chromosome 19 appeared to alter position so that it localized near to nucleolar structures. This was not a random change, since it was not seen with chromosome 17 probes. Together, these results show that RB-induced arrest involves extensive changes in nuclear organization. Such effects are consistent with the idea that RB is a master regulator of cell proliferation (Hanahan and Weinberg, 2000; Rubin et al., 2020; Sherr, 1996; Weinberg, 1991), and they agree with previous work showing that RB inactivation leads to widespread changes in epigenetic marks (Zhang et al., 2012). The results also fit with recent studies showing that tumorigenesis, malignancy, and senescence are associated with major reorganization of chromatin and chromatin interactions within the nucleus (Chandra et al., 2015; Criscione et al., 2016; Johnstone et al., 2020).

Strikingly, RB-induced dispersion was evident in chromosome domains that are rich in RB-bound loci, as well as those that lack RB-binding sites. RB ChIP-seq data showed that the two euchromatin regions examined here are highly enriched in RB peaks, whereas the two tested heterochromatin regions lacked any mapped RB-binding sites. The fact that all of these regions showed RB-induced dispersion can be interpreted in three different ways. One interpretation is that the reorganization of chromatin domains following the activation of RB is not a direct effect of RB action but an indirect consequence that is triggered by active RB. Thus, dispersion occurs in chromosome domains regardless of the number of RB-binding sites that they contain. An alternative explanation suggested by the work of Dick and colleagues (Dick et al., 2018, and Ishak et al., 2016) is that RB association with heterochromatin is not be easily detected by ChIP-seq, and that the absence of mapped E2F/RB-binding sites in the regions targeted by the α-satellite probes reflects regulation by noncanonical RB activity. In this scenario, the effects of RB on the organization of heterochromatic regions may be directly mediated by RB, but they occur via a chromatin binding activity that has yet to be elucidated. A third interpretation, and one that we favor, is that dispersion is a complex phenotype, that multiple pathways contribute to the reorganization seen under the microscope, and that RB controls euchromatin and heterochromatin regions differently. In this interpretation, the chromatin changes could involve both direct and indirect effects of RB and may well be mediated by various types of RB-binding sites. In this model, the dispersion of euchromatic regions is likely driven by changes mediated via RB-binding sites, while the changes in the organization of heterochromatic regions may be indirect effects of RB or effects mediated via noncanonical binding sites. This notion of dispersion as a complex set of events could explain why 3C experiments on euchromatic regions detected changes in contacts between RB-bound loci while heterochromatin domains lack any mapped RB-binding sites. Such complexity could also explain why some forms of RB disperse euchromatic and heterochromatic regions to different extents.

Regardless of the model, the bottom line is that the RB-induced changes in organization are a noncanonical activity of RB (Dick et al., 2018; Ishak et al., 2016). The changes were visible in regions of the genome that lacked well-studied E2F targets, they occurred after cell cycle arrest, the changes did not correlate with repression of E2F targets, and they were not blocked by a DNDP1 construct that suppressed RB recruitment to cell cycle–regulated promoters, yet they were dependent on HDAC and TOPII activities, proteins known to interact with RB on chromatin. The 3C results suggest that RB activation particularly alters interactions between pairs of RB-bound loci. Together, these observations suggest that RB-induced dispersion involves activities of RB on DNA, but it is clear that they are induced by a mechanism that is different from canonical RB/E2F regulation. The size of the regions that undergo visible changes is impressive, and it demonstrates that the impact of RB-mediated regulation is not restricted to the local environment of RB-binding sites. We conclude that, rather than simply controlling local chromatin marks, RB helps to reconfigure the organization of large chromosomal regions, and we hypothesize that, in doing so, RB is able to coordinate transcriptional changes over large regions. RB has been shown to influence the association of Condensin II complexes with chromatin (Kim et al., 2021). While we did not observe an effect of CapD3 depletion on dispersion, it remains possible that changes in Condensin II distribution contribute to the reorganization.

The finding that some mP-RBs strongly promote dispersion, while others are almost completely deficient for this activity, adds to an emerging view that there is not a single form of functional RB, but many variations of RB that have distinct properties (Dowdy, 2018; Narasimha et al., 2014; Sanidas et al., 2019). These functional differences enabled us to cleanly separate RB-induced dispersion from E2F inhibition and cell cycle arrest. Given that the expression of any of the 14 mP-RBs suppresses E2F and causes cell accumulation in G1 (Sanidas et al., 2019), neither of these RB activities are sufficient for RB-induced dispersion. Importantly, the functional variation between mP-RBs made it possible to identify transcriptional changes that are associated with dispersion. The clear, but unexpected, result is that RB-induced dispersion in RPE1 cells was associated with increased expression of a program of autophagy genes. This finding was confirmed by quantitative RT-PCR and was supported by a time-dependent increase in LC3B-II–stained autophagosomes that paralleled dispersion.

A small but significant literature has linked RB to autophagy. Ectopic expression of RB in cancers defective for RB or the treatment of cancer cells with CDK inhibitors induces autophagy (Jiang et al., 2010; Komata et al., 2003; Liang et al., 2007; Vijayaraghavan et al., 2017). Indeed, RB loss inhibits the transcription of autophagy genes in small-cell lung cancer (Cochrane et al., 2020). The analysis of RB ChIP-seq data shows that 74% of the autophagy genes whose expression correlated with dispersion (*r* > 0.6) have RB-binding sites in their promoter or enhancer sequences (Table S2). Currently it is unknown whether RB-induced dispersion is responsible for the increase in gene expression, but the presence of RB-binding sites at autophagy genes certainly gives RB the opportunity to directly modulate autophagy responses. Further genome-wide interaction studies are needed to catalog the full extent of RB-induced changes in chromatin contacts and to link them to specific transcriptional programs.

One of the most interesting features of the data described here is that the results provide insight into the cellular context in which active RB drives the reorganization of chromosomal domains. Historically, cell cycle–dependent activation and inactivation of RB was first discovered in dividing cells (Buchkovich et al., 1989; DeCaprio et al., 1989). However, RB-induced changes in chromatin organization were difficult to discern in dividing cells with FISH probes. Instead, the changes described here appeared in a time-dependent manner when cells were driven into a stable RB-mediated cell cycle exit such as occurred during IR-induced senescence and multiday palbociclib treatment and following ΔCDK-RB expression. Similarly, CDK4/6 inhibition induces RB-dependent chromatin accessibility changes in breast cancer cell lines that are reliant on time (Watt et al., 2021). The idea that dispersion is a context-specific property of RB that is seen when RB is activated for an extended period of time may help to explain why it was missed in earlier studies.

The RB-dependent exit from the cell cycle is part of the mechanism that leads to cellular senescence. It is important to note that the RB-induced chromatin dispersion is not identical to other changes observed in senescent cells. The RB-mediated changes are distinct from SAHFs, large aggregates of heterochromatin that are apparent in some senescent cells (Chandra et al., 2015; Chandra and Narita, 2013; Chicas et al., 2010; Narita et al., 2003), or senescence-associated heterochromatin domains (Sati et al., 2020). RB-induced dispersion occurs in both regions of euchromatin and heterochromatin, it was not associated with increased H3K9me3 staining, and the genes within the reorganized domains remained actively transcribed. Indeed, SAHFs/senescence-associated heterochromatin domains were not evident in the RPE1 cells that displayed RB-induced dispersion. Although RB-mediated dispersion is similar to senescence-associated distension of α-satellites (Swanson et al., 2013), it is different from the changes in chromosome compaction that are observed in replicative senescent fibroblasts (Criscione et al., 2016). RB-induced dispersion was observed in both heterochromatin and euchromatin, with consequences for cellular gene expression programs. We interpret these distinctions as evidence that the RB-specific effects are just one component of a larger network of changes that occur during senescence and, since they occur within 48–72 h of RB activation, are precursors to more extensive rearrangements.

The fact that visible changes in euchromatin and heterochromatin regions were induced by RB in the context of stable cell cycle exit highlights the intriguing possibility that the RB-induced changes in chromosome and nuclear organization may not be just a feature of stably arrested or presenescent cells, but may also be part of the mechanisms that enforce permanent cell cycle exit. DOX washout experiments, after 3-d induction of ΔCDK-RB, failed to reverse dispersion. Potentially, RB-induced dispersion may involve topoisomerase-dependent changes that tangle chromatin to such an extent that it is challenging for the cell to reverse these effects. Clearly, further work will be needed to understand the regulation, mechanisms, and long-term consequences of RB-mediated changes in the organization of chromosomal domains.

## Materials and methods

### Cell culture

Telomerase-expressing nontransformed human retina epithelial cells (RPE1) were kindly provided by Dr. David Pellman’s laboratory (Dana Farber Cancer Institute, Boston, MA). RPE1 cells were cultured in DMEM (15013CM; Corning) supplemented with 5% tetracycline-free FBS (631367; Takara), 2 mM L-glutamine (25030081; Gibco BRL), and antibiotics (100 units/ml of penicillin and streptomycin; 15070063; Gibco BRL). IMR-90 (CCL-186; American Type Culture Collection), a human diploid lung fibroblast cell line, was cultured in modified Eagle media supplemented with 10% tetracycline-free FBS and antibiotics (100 units/ml penicillin and 100 µg/ml streptomycin). To serum-starve RPE1 cells, we cultured them for 3 d in DMEM supplemented with 0.1% tetracycline-free FBS, 2 mM L-glutamine, and antibiotics (100 units/ml penicillin and streptomycin).

RB1 knockdown or replacement of endogenous by exogenous RB protein in RPE1 cells transduced with pINDUCER11-shRB1 and/or pINDUCER20-FLAG-RB constructs was induced by 2 µg/ml DOX (D9891; Sigma-Aldrich) for 72 h. The above constructs and cell lines were described before by Sanidas et al. (2019). Senescence was induced in IMR-90 cells by 10 Gy γ-IR treatment, followed by splitting cells every 3 d for 12 d. To inhibit CDK4/6 kinase activity and observe dispersion, RPE1 cells were treated with 1 µM palbociclib (CT-PD2991; ChemieTek) for 72 or 120 h. To inhibit HDACs, RPE1 cells were treated with 0.8 µM TSA for 72 h. To inhibit EZH2 activity in RPE1 cells, cells were treated with 5 µM GSK-126 for 72 h (Khan et al., 2015). To inhibit TOPII, we added 0.5 µM etoposide 24 h before harvesting. To inhibit transcriptional activity, we added 80 µM DRB or DMSO to WT and ΔCDK- RB cells after 48 h of DOX induction. Cells were kept on DRB for 24 h and harvested after 72 h of DOX induction.

For autophagy time course experiments, ΔCDK RB, WT, and mP-RB cells were induced with DOX for 24, 48, 72, and 96 h. To accurately determine autophagy flux, 100 nM bafilomycin was added to the medium 3, 24, 24, and 24 h, respectively, before fixing for immunofluorescence (IF) experiments. For autophagy epistasis experiments, 100 nM bafilomycin was added 3 h before fixing for IF experiment.

### siRNA transfection and knockdown

Cells were seeded 16 h before siRNA transfection. Cells were transfected with siRNAs against TOPIIβ, TOPIIα, CAPD3, WAPL (TOPIIβ, L004240-00-0005; TOPIIα, L004239-00-0005; CAPD3, M-026539-01-0005; WAPL, L-026287-01-0005; Dharmacon). The sequences of siRNA pool used for knockdown were TOPIIβ, 5′-GAA​GUU​GUC​UGU​UGA​GAG​A-3′, 5′-CGA​AAG​ACC​UAA​AUA​CAC​A-3′, 5′-GAU​CAU​AUG​GGA​UGU​CUG​A-3′, and 5′-GGU​GUA​UGA​UGA​AGA​UGU​A-3′; TOPIIα, 5′-CGA​AAG​GAA​UGG​UUA​ACU​A-3′, 5′-GAU​GAA​CUC​UGC​AGG​CUA​A-3′, 5′-GGA​GAA​GAU​UAU​ACA​UGU​A-3′, and 5′-GGU​AAC​UCC​UUG​AAA​GUA​A-3′; CAPD3, 5′-GAG​AUA​AGG​UCA​UCA​GUU​G-3′, 5′-GAG​UCA​CCC​UGG​UAC​CUU​A-3′, 5′-UAU​GUU​UGA​UCG​UUG​CUU​A-3′, and 5′-GUA​CAC​UGG​UGG​CAU​UUU-3′; and WAPL, 5′-GGA​GUA​UAG​UGC​UCG​GAA​U-3′, 5′-GAG​AGA​UGU​UUA​CGA​GUU​U-3′, 5′-CAA​ACA​GUG​AAU​CGA​GUA​A-3′, and 5′-CCA​AAG​AUA​CAC​GGG​AUU​A-3′). After 24 h, transfection medium was removed and replaced with DOX-containing medium to induce either WT RB or ΔCDK-RB expression. After another 24 h, cells were split into new wells. Cells were transfected once more with siRNAs after 60 h of DOX addition. Cells were harvested or fixed after 72-h DOX induction was completed. The final concentration of siRNAs used was 20 nM for all the above gene targets.

### FISH

#### Oligopaints FISH (euchromatin FISH)

RPE1 cells were spotted onto slides using cytospin (1,000 RPM for 2 min) and fixed with methanol/acetic acid (3:1) solution for 15 min, followed by two washes with 1× PBS for 5 min each. Slides were dried and then washed in 50% formamide (Sigma-Aldrich)/2× saline-sodium citrate buffer (SSCT) at 78°C for 2 min, followed by 50% formamide/2× SSCT at 60°C for 20 min. Probe mix (19q13.42 or 19q13.2 probes at final concentrations of 1 pM/µl), 2× SSCT, 10% dextran sulfate, and 1 µg RNaseA (EN0531; Thermo Fisher Scientific) was added to the samples, and coverslips were affixed and sealed using rubber cement. Slides were denatured at 78°C for 2 min and incubated at 37°C for 42–46 h. Coverslips were removed, and slides were washed in 2× SSCT at 60°C for 15 min, followed by 2× SSCT and 0.2× SSC washes for 2 min each. Slides were mounted then with Vectashield (with DAPI, H1200; Vector Laboratories) and sealed.

#### Heterochromatin FISH

Coverslips with adhered RPE1 cells were washed with PBS and fixed by sequentially incubating in 70, 85, and 100% ethanol for 2 min each. Coverslips were dried, and probe mix was added for chromosome 7 α-satellite (LPE007G; hybridization buffer B, H1000L; Cytocell) or chromosome 6 α-satellite (LPE006R; hybridization buffer B, H1000L; Cytocell). Coverslips were affixed to slides and sealed using rubber cement. Slides were denatured at 75°C and incubated at 37°C for 16 h. Coverslips were removed and washed in 0.25× SSC at 72°C and 2× SSC (with 0.05% Tween-20) for 2 min each. Coverslips were mounted using Vectashield (with DAPI) and sealed.

#### Whole-chromosome FISH

Coverslips with adhered RPE1 cells were washed with PBS and fixed by sequentially incubating in 70, 85,and 100% ethanol for 2 min each. Coverslips were dried, probe mix for chromosome 19 or 17 was added (LPP19G-A, LPP17G-A; Cytocell), and coverslips were affixed onto slides and sealed using rubber cement. Slides were denatured at 75°C and incubated at 37°C for 16 h. Coverslips were removed and washed in 0.4× SSC at 72°C and 2× SSC (with 0.05% Tween-20) for 2 min each. Coverslips were mounted using Vectashield (with DAPI) and sealed.

### IF

Coverslips with adhered RPE1 cells were washed with PBS and fixed with methanol for 10 min at −20°C. Coverslips were washed with PBS two times for 2 min. Cells were permeabilized using permeabilizing buffer (PBS and 0.5% Triton X-100) for 3 min on ice. Coverslips were washed twice with PBS for 2 min. Blocking buffer (1% BSA and 0.05% Tween-20) was added for 1 h. Primary antibodies (LC3B rabbit polyclonal antibody; 1:200; NB600-1384; Novus Biologicals; LAMP2 mouse monoclonal antibody; 1:100; ab25631; Abcam) were diluted in blocking buffer,added on coverslips, and incubated overnight. Coverslips were washed three times with PBS for 5 min each. Secondary antibodies (Alexa Fluor 488-goat anti-rabbit IgG secondary antibody; A-11008; Invitrogen; Alexa Fluor 568-goat anti-mouse IgG secondary antibody; A-11004; Invitrogen) were diluted 1:500 in blocking buffer and incubated for 1 h at RT. Coverslips were washed three times with PBS for 5 min and stained with DAPI. Coverslips were mounted using Vectashield (H1000; Vector Laboratories) and sealed.

### Microscopy, quantitation, and analysis of FISH and IF images

A Zeiss LSM 710 Laser Scanning Confocal Microscope and Zeiss Zen software were used to acquire FISH images. Images were captured using parameters for each probe that remained consistent across samples and experiments. Images were acquired at RT, and a Plan-Apochromat 63×/1.40 Oil DIC M27 was used to capture Z-stacks (6–12 sections of 0.5-µM thickness). FITC/Alexa Fluor 488 filter was used to acquire images for chromosome 7 α-satellite, 19q13.42, and whole-chromosome 19 and 17 probes. Alexa Fluor 568/Texas Red filter was used to acquire images for chromosome 6 α-satellite and 19q13.2 probes. Maximum projections of Z-stacks were used for image analysis. No other image processing was performed. Images were analyzed using Fiji (ImageJ) software. To effectively quantify the dispersion phenotype, we used a skeleton dot length plugin, and macros were made to execute the steps outlined in Fig. 1, C and D. The following macro steps were written and executed in FIJI: open();setOption(“BlackBackground”, false); run(“Make Binary”); run(“Dilate”); run(“Dilate”); run(“Dilate”); run(“Skeletonize”); run(“Analyze Skeleton (2D/3D)”, “prune=none calculate show display”). Cutoffs of skeleton dot length were used to define the foci categories as compact, diffused, dispersed, and scattered as follows. For heterochromatin regions (chromosome 7 α-satellite, chromosome 6 α-satellite), foci with skeleton dot length <0.75 µM were called compact, 0.75–1.25 µM diffused, and >1.25 µM dispersed. For euchromatin regions (19q13.42 and 19q13.2), foci with skeleton dot length <1.5 µM were called compact/diffused, 1.5–2.5 µM dispersed, and >2.5 µM scattered.

For IF images, a Zeiss LSM 780 Laser Scanning Confocal Microscope and Zeiss Zen software were used to acquire images. A Plan-Apochromat 63×/1.40 Oil DIC M27 was used to capture snaps. Images were quantified using Fiji (ImageJ). Cytoplasmic LC3B-II puncta were counted using Fiji (ImageJ). Four steps were performed to quantify the puncta: (1) individual cells were cropped/segmented out, (2) images were converted into binary scale using the default or moments dark threshold function, (3) background outliers (punta <1 µM) were removed (if any), and (4) puncta were counted by analyze particles function. Macros were made to execute these steps.

### Cell cycle analysis

After 3 d of DOX induction, RPE1 cells were incubated with 20 µM 5′-ethynyl-2’- deoxyuridine (EdU) for 2 h, fixed with 3.7% formaldehyde in PBS for 15 min, blocked with 3% BSA in PBS for 1 min, permeabilized with 0.5% Triton X-100 in PBS for 30 min, and stained with ClickIT reaction (100 mM Tris-HCl, pH 7.5, 3 mM CuSO_4,_ 50 mM ascorbic acid, and 2.5 µM Alexa Fluor 647-azide [A10277; Life Technologies]) for 30 min and 3 µM DAPI (D1306; Life Technologies) in staining buffer (100 mM Tris-HCl, pH 7.5, 150 mM NaCl, 1 mM CaCl_2_, 0.5 mM MgCl_2_, and 0.1% NP-40) for 15 min. FACS analysis was performed using a LSR II flow cytometer (BD Biosciences), and data were analyzed using FlowJo software.

### Western blot method

Cells were washed with PBS and lysed using the following buffer: 187.5 mM Tris-HCl, pH 6.8, 30% glycerol, 0.03% Bromophenol Blue, 10% SDS [wt/vol], and 42 mM dithiothreitol). Lysates were boiled for 10 min and vigorously vortexed in between every 3 min. Lysates were analyzed using Bio-Rad gel and transferred to PVDF membranes (1704273; Bio-Rad). Primary antibodies were diluted in 5% BSA (P-753; Boston BioProducts) in TBS containing 0.1% Tween 20 (Bio-Rad). The following primary antibodies were used: WAPL (D9J1U) rabbit monoclonal antibody (1:1,000, 77428; Cell Signaling Technologies), TOPIIβ rabbit polyclonal antibody (1:2,000, ab72334; Abcam), TOPIIα rabbit monoclonal antibody (1:2,000, ab52934; Abcam), CAPD3 rabbit polyclonal antibody (1:2,000, A300-604A; Bethyl Laboratories), RB1 (4H1) mouse monoclonal antibody (1:1,000, 9309; Cell Signaling Technologies), tri-methyl-histone H3 (Lys27) rabbit monoclonal antibody (C36B11, 9733S; Cell Signaling Technologies), anti-histone H3 rabbit polyclonal antibody (ab1791; Abcam), phospho-histone H2A.X (Ser139; 20E3; 1:1,000, 9718; Cell Signaling Technologies), and anti-FLAG (M5; 1:5,000, F4042; Sigma-Aldrich). Secondary antibodies were obtained from Cell Signaling Technologies (7074S, anti-rabbit IgG, HRP-linked antibody; 7076S, anti-mouse IgG, HRP-linked antibody) diluted in 5% milk at 1:5,000. Molecular weight in the figures was estimated by comparing to molecular weight markers.

### β-Galactosidase assay

The assay was performed using Cellular Senescence Assay Kit (KAA002; EMD Millipore). Culture plate wells with adhered RPE1 cells were washed with 1× PBS and fixed with 1× fixing solution for 15 min. Cells were washed twice with 1× PBS. 1× SA-β-galactosidase detection solution was added to the cells and incubated overnight in dark at 37°C. Cells were washed with 1× PBS, and the stained cells were imaged under bright-field light microscopy using an ECHO Revolve microscope.

### Quantitative PCR (qPCR)

RNA was extracted using RNeasy kit (74104; Qiagen) and DNase treatment of RNA was performed before any downstream applications (79254; Qiagen). cDNA was prepared using Taqman Reverse Transcription Reagents (2066622; Life Technologies). qPCR was performed using Lightcycler 480 SYBR Green I Master (04913914001; Roche) and was run using a Roche LightCycler 480 system. β-Actin qPCR was run as internal control for each sample/time point. qPCR primers used for IMR-90 senescence time course were β-actin_F, 5′-CAC​CAT​GTA​CCC​TGG​CAT​TG-3′; β-actin_R, 5′-GTA​CTT​GCG​CTC​AGG​AGG​AG-3′; LMNB1_F, 5′-AAG​CAT​GAA​ACG​CGC​TTG​G-3′; LMNB1_R, 5′-AGT​TTG​GCA​TGG​TAA​GTC​TGC-3′; ICAM1_F, 5′-ATG​CCC​AGA​CAT​CTG​TGT​CC-3′; ICAM1_R, 5′-GGG​GTC​TCT​ATG​CCC​AAC​AA-3′; HDDC2_F, 5′-GGG​CAG​CTC​AAG​AGA​GTC​C-3′; HDDC2_R, 5′-GGC​GTA​CAC​ATC​GGT​CTT​TGT-3′; CCND1_F, 5′-CAA​TGA​CCC​CGC​ACG​ATT​TC-3′; and CCND_R, 5′-CAT​GGA​GGG​CGG​ATT​GGA​A-3′. qPCR primers used to measure expression of autophagy genes were β-actin_F, 5′-CAC​CAT​GTA​CCC​TGG​CAT​TG-3′; β-actin_R: 5′-GTA​CTT​GCG​CTC​AGG​AGG​AG-3′; LC3B_F, 5′-ACG​CAT​TTG​CCA​TCA​CAG​TTG-3′; LC3B_R, 5′-TCT​CTT​AGG​AGT​CAG​GGA​CCT​TCA​G-3′; p62_F, 5′-GAC​TAC​GAC​TTG​TGT​AGC​GTC-3′; p62_R, 5′-AGT​GTC​CGT​GTT​TCA​CCT​TCC-3′; ATG14_F, 5′-GCG​CCA​AAT​GCG​TTC​AGA​G-3′; ATG14_R: 5′-AGT​CGG​CTT​AAC​CTT​TCC​TTC​T-3′; WIPI1_F, 5′-AAC​AGG​TCT​ATG​TGC​TCT​CTC​T-3′; WIPI_R, 5′-CTC​ATG​GGC​AGC​AAT​AGT​GC-3′; ATG4B_F, 5′-ATG​GAC​GCA​GCT​ACT​CTG​AC-3′; ATG4_R: 5′-TTT​TCT​ACC​CAG​TAT​CCA​AAC​GG-3′; VCP_F: 5′-CAA​ACA​GAA​GAA​CCG​TCC​CAA-3′; VCP_R, 5′-TCA​CCT​CGG​AAC​AAC​TGC​AAT-3′; PSAP_F, 5′-ATG​CAA​AGA​CGT​TGT​CAC​CG-3′; PSAP_R, 5′-GGG​AGG​TAG​GAG​TCC​ACT​ATC​T-3′; SEC23B_F, 5′-GCT​GGA​GGC​TAC​AAG​AAT​GGT-3′; and SEC23B_R, 5′-AAC​CTG​ACA​AAG​TGG​GTT​GAG-3′.

### PLA and analysis

PLA assay was performed using a Duolink In Situ PLA GREEN kit from Sigma-Aldrich and following manufacturer instructions. WT and ΔCDK-RB RPE cells were incubated at 37°C for 24 and 48 h in the presence of 2 µg/ml DOX on glass coverslips (EdU incubation was performed at 37°C for 30 min in the presence of 10 µM). Cells were then washed twice with cold PBS and fixed with 3.7% formaldehyde in PBS for 15 min at RT and permeabilized for 10 min at RT with 0.5% Triton X-100 in PBS. Blocking was performed with 2.5% BSA in PBS. EdU was detected using Click-iT EdU Cell Proliferation Kit for Imaging, Alexa Fluor 647 (C10340; Thermo Fisher Scientific). Primary antibody incubation was carried at RT for 2 h using mouse-α-RB (9309, 1:400; Cell Signaling Technologies) and rabbit-α-TOPIIβ (ab72334, 1:150; Abcam). Cells were then incubated with α-rabbit PLA plus probe and α-mouse PLA minus probe for 1 h at 37°C. Ligation reaction was then performed for 30 min at 37°C followed by amplification reaction for 100 min at 37°C. Finally, coverslips were mounted using SlowFade Diamond Antifade Mountant with DAPI (S36964; Thermo Fisher Scientific). Images were taken with an ECHO Revolve microscope; images were analyzed and fluorescence was quantified with Matlab software. For analysis, in brief, cell nuclei were segmented using a custom-made image-processing pipeline that can distinguish them from the background. The pipeline identifies the nuclei based on DAPI staining, size, and circularity. Nuclei that were close to image borders or too close to each other and could not be individually segmented were automatically removed by the software. PLA foci numbers and PLA foci and EdU fluorescence intensities were quantified by the software only within the segmented nuclei. Edu-positive cells were classified as S phase cells, and Edu-negative cells were classified as either G1 or G2 cells (based on DAPI signal intensity).

### 3C and quantification

5 million to 10 million cells were used per experiment, and 3C was done according to the protocol in Hagège et al. (2007) with modifications. The DNA obtained at the end was further purified using PureLINK Genomic DNA Extraction/Purification kit (K182002; Thermo Fisher Scientific). qPCR was done using Lightcycler 480 SYBR Green I Master (04913914001; Roche). For qPCR, 1 µg per reaction of DNA and 1 µM primer per reaction was used. For analysis, we normalized the crossing point (C_p_) values to the average C_p_ values obtained across all tested loci for a particular sample. Fold-changes were calculated using origin ligation product (for TAD boundaries, TAD4-reg1; for RB binding sites, KMT5C) as the control. Fold-change was calculated using the formula 2^(^*^Cp^*^ − ^*^CpORIGIN^*^)^, where *C_p_* is the normalized qPCR signal obtained for a specific locus, and *C_pORIGIN_* is the qPCR signal obtained for the origin self-ligation product. Three biological replicates were performed for each sample, and mean fold-change was calculated and plotted. P values were calculated using multiple *t* test and Holm–Sidak multiple comparison with GraphPad Prism 6.0 software.

To determine interactions between TAD boundaries, qPCR primers were designed around HindIII sites located at the edge of TADs. All TAD boundary interactions were measured with respect to the origin, which was TAD4-reg1. The following primers were used: TAD4-reg1 (origin primer), 5′-CAT​CAG​ACA​AGC​CCT​CCC​TC-3′; TAD4-reg1, 5′-TTG​GGC​TTT​CCT​GTG​CTT​CT-3′; TAD4-reg2, 5′-TTG​TCT​AAG​TCG​CTG​CTG​GG-3′; TAD4-reg3, 5′-ACA​GGT​GCT​TAT​GTT​TGC​ATT​CT-3′; TAD3-reg1, 5′-GTG​CAG​GGG​TAC​AGA​ACA​GA-3′; TAD3-reg2, 5′-CCA​GCC​ACC​GAC​AAT​TCC​TT-3′; TAD2-reg1, 5′-TAT​TGG​ATG​CCA​GCA​GAG​GC-3′; TAD2-reg2, 5′-ACG​GTA​TCC​CTC​TTC​CCC​TT-3′; and TAD2-reg3, 5′-GGA​GAG​ACA​GGA​GAT​GGG​GA-3′. For interactions between RB bound sites located in TAD loops, qPCR primers were designed around HindIII sites located within RB binding sites or closest to binding site. All loop interactions were measured with respect to the origin, which was KMT5C. The following primers were used: KMT5C (origin primer), 5′-TGG​GAC​AGC​TCC​TCT​TTC​CA-3′; KMT5C, 5′-GGC​TTT​CTC​CCT​CCT​GTG​G-3′; UBE2S, 5′-CCG​TCT​GCT​CAC​AGA​GAT​CC-3′; U2AF2, 5′-CAC​AGG​CCA​CAG​AAT​GGT​CT-3′; ZNF524, 5′-ACG​TAC​TTG​CCC​AGA​CAT​GC-3′; SSC5D, 5′-TGA​AAT​TGC​AAC​TGA​GGG​GG-3′; HSPBP1, 5′-GGA​CCC​TAT​GAC​GAG​CAC​AG-3′; PPP6R1, 5′-GGG​CAG​AGT​TAG​GGT​TAC​AGT​G-3′; PTPRH, 5′-TGG​GGG​AAT​TTC​TAG​GGG​CT-3′; and PRPF31, 5′-TTT​TAA​GCC​CCT​CTC​CCC​AG-3′.

### DNA damage assay

To determine the effect of DNA damage on dispersion, damage was induced in WT RB and DCDK RB–expressing cells using 10Gy ionizing radiation and camptothecin. We chose these damaging agents because they have the ability to generate damage in cells arrested in G1 as well as later stages. Cells were treated with IR after 72-h DOX induction of ΔCDK-RB or WT RB (control). Cells were fixed/harvested 2 h after IR treatment. 1 µM camptothecin was added to ΔCDK-RB or WT RB (control) cells between 48 and 72 h of DOX induction. Cells were fixed/harvested after the completion of 72-h DOX induction. Lysates for Western blot were prepared as noted above, and FISH for the chromosome 7 α-satellite was performed as stated above.

### MNase assay

The MNase assay was performed using 2 million RPE1 cells per sample. Cells were trypsinized and resuspended in 400 µl of ice-cold Buffer A (10 mM Hepes-KOH, pH 7.5, 10 mM KCl, 1.5 mM MgCl_2,_ 0.5 mM dithiothreitol, 1 mM PMSF, and protease inhibitor cocktail [04693159001; Roche]). Cells were allowed to swell by keeping them on ice for 10 min. 400 µl of ice-cold Buffer A + 0.4% NP-40 was added and incubated on ice for 10 min. Cells were centrifuged at 300 *g* for 5 min at 4°C to pellet nuclei and other fragments. The isolated nuclei were washed with 1 ml PBS without disturbing the pellet and centrifuged at 2,800 *g* for 5 min at 4°C. The pellet was resuspended in 300 µl of 1× Micrococcal Nuclease Buffer (supplemented with 100 µg/ml BSA) and either 1,500 or 40 units of Micrococcal Nuclease (2,000 units/µl; M0247S; New England BioLabs) and incubated at 37°C on a shaker (300 rpm) for various time intervals. 45 µl of reaction was taken out at every time interval, and digestion was stopped by adding 5 µl of 0.5 M EGTA and 200 ng RNase A. Reactions were incubated at 37°C for 1 h and supplemented with 1% SDS and 10 µg of proteinase K (EO0491; Thermo Fisher Scientific) at 55°C for ≥2 h. To extract DNA, an equal volume of phenol/chloroform/isoamyl alcohol (25:24:1, P2069; Sigma-Aldrich) was added to the samples and vortexed vigorously. Samples were centrifuged for 5 min at 16,000 *g*, and the upper aqueous layer was transferred to a new tube. To precipitate the DNA, glycogen (20 µg), NH_4_OAc (7.5 M, 0.5 volume), and 100% (2.5 volume) ethanol was added to the aqueous phase. Precipitated DNA was washed twice with 70% ethanol and resuspended in H_2_O. DNA was quantified using Nanodrop, and 1 µg of DNA per lane was loaded onto an 0.8% agarose gel.

### ChIP

ChIP assays were performed using 7.5 million RPE1 cells per sample and per immunoprecipitation. Briefly, cells were fixed with 1.5 mM ethylene glycol bis (succinimidyl succinate) (21565; Thermo Fisher Scientific) for 30 min at RT and 1% formaldehyde (F8775; Sigma-Aldrich) for 10 min at 37°C. Chromatin was fragmented to a size range of 200–700 bases with a Branson 250 sonicator. Solubilized chromatin was immunoprecipitated with FLAG rabbit monoclonal antibody (14793; Cell Signaling Technologies) or E2F1 rabbit polyclonal antibody (3742; Cell Signaling Technologies) overnight at 4°C. Antibody–chromatin complexes were purified with Dynabeads Protein G (10004; Invitrogen). After cross-link reversal and RNase A and proteinase K treatment, immunoprecipitated DNA was extracted with AMP Pure XP beads (A63881; Beckman Coulter) and analyzed by SYBR-Green real-time qPCR, along with the input DNA using the Roche LightCycler 480 system (Boulay et al., 2017). ChIP real-time qPCR primers used were MCM4_F, 5′-CCG​AGC​GAG​GCC​TAC​TTC​T-3′; MCM4_R, 5′-GGA​CAG​TGC​CGC​TTC​TTT​CA-3′; MCM3_F, 5′-TCT​TTG​GCA​GCG​GGC​AT-3′; and MCM3_R, 5′-CGC​AGC​TCC​ACA​TCG​TCC-3′. Average scores from three technical replicates were calculated per sample and per epitope. P values were calculated with the Holm–Sidak multiple *t* test using GraphPad Prism 8.0 software.

### Statistical Analysis

Figures of quantifications were assembled, and statistics were run in GraphPad Prism 6. For comparisons between sample pairs, datasets were analyzed by the nonparametric two-tailed Mann–Whitney *U* test, and exact P values were reported. For multiple comparisons, datasets were analyzed by nonparametric Kruskal–Wallis–Dunn’s multiple comparison test, one-way ANOVA–Dunnett’s multiple comparison test, and multiple *t* tests. P values are displayed as ns, P > 0.05; *, P ≤ 0.05; **, P ≤ 0.01; ***, P ≤ 0.001; and ****, P ≤ 0.0001.

### Online supplemental material

Fig. S1 shows dispersion, RB monophosphorylation status, β-galactosidase staining, senescence/senescence-associated secretory phenotype signature for IR-induced senescence time course in IMR-90 cells, and percentage of G1 phase cells and RB phosphorylation status after palbociclib treatment. Fig. S2 shows the DOX-inducible simultaneous ΔCDK/mP-RB and shRB1 expression system; cell cycle profile for serum-starved WT and ΔCDK RPE cells; and percentage of dispersed and compact heterochromatin and euchromatin foci for the various RB monophosphorylation forms. Fig. S3 shows the effect of DNDP1 expression on RB, E2F chromatin binding, and cell cycle profile and dispersion; the effect of EZH2 inhibitor TOPIIα knockdown on dispersion; and confirmatory Western blots for EZH2 inhibitor treatment, Trichostatin A treatment, and TOPIIα and TOPIIβ knockdown. Fig. S4 shows proximal ligation assay for detecting RB-TOPIIβ interaction; the effect of DNA damaging agents on dispersion and corresponding damage readout (γH2AX Western blot); the effect of WAPL knockdown, CAPD3 knockdown, and DRB treatment on dispersion; and MNase-based chromatin accessibility assay for WT and ΔCDK RPE cells. Fig. S5 shows nucleolar associated domain coordinates for the 19q13.42 region and the proximity of this region to nucleolus; autophagosome levels (LC3B) in mP-RB 252 and WT cells treated with etoposide; and expression levels of some autophagy-related genes in high, medium, and low chromatin dispersers. Table S1 shows results of GO analysis of transcripts positively correlating with dispersion. Table S2 lists genes positively correlating (*r* > 0.6) with chromatin dispersion and RB binding status.

## Supplementary Material

Table S1 shows the results of GO analysis of transcripts positively correlating with dispersion.

Table S2 lists genes positively correlating (*r* > 0.6) with chromatin dispersion and RB binding status.

SourceData FS1 contains original blots for Fig. S1.

SourceData FS3 contains original blots for Fig. S3.

SourceData FS4 contains original blots for Fig. S4.
