# Supplementary material for: Active RB causes visible changes in nuclear organization
Source: J Cell Biol. 2022 Jan 12;221(3):e202102144. doi: 10.1083/jcb.202102144 (PMC8759594; doi:10.1083/jcb.202102144)
Supplement: SourceData FS1 — contains original blots for Fig. S1. [file JCB_202102144_SourceDataFS1.pdf]

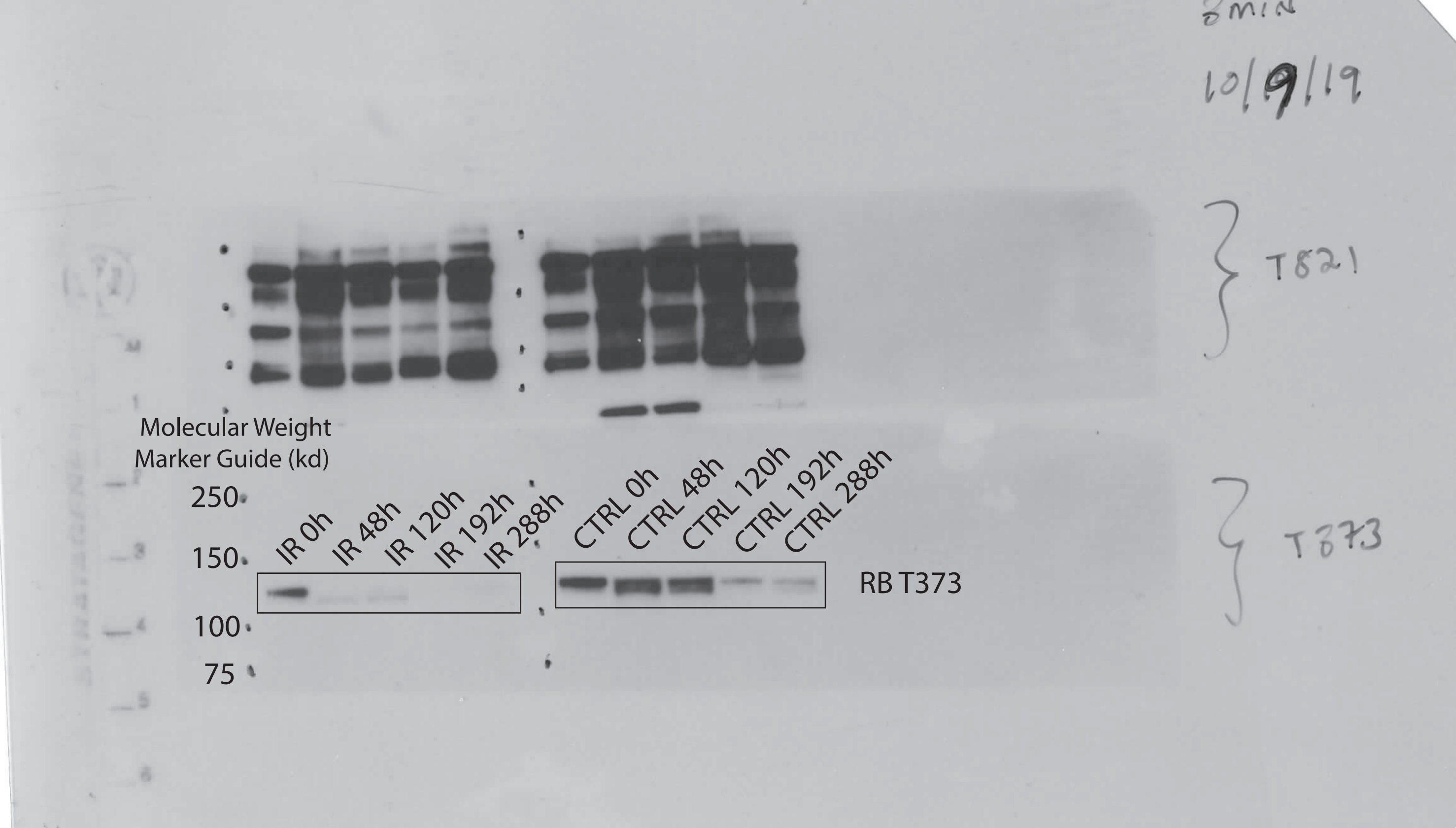

8min
10/9/19
T821
T373
Molecular Weight
Marker Guide (kd)
250
150
100
75
IR 0h
IR 48h
IR 120h
IR 192h
IR 288h
CTRL 0h
CTRL 48h
CTRL 120h
CTRL 192h
CTRL 288h
RB T373

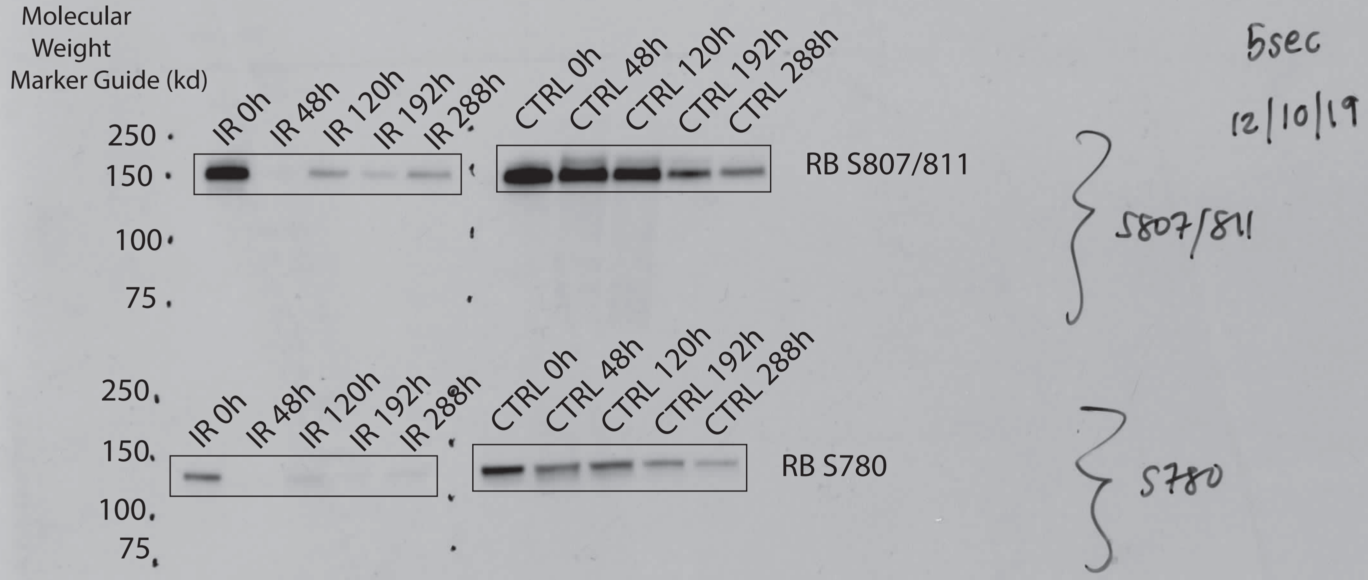
Molecular Weight Marker Guide (kd)
250
150
100
75
IR 0h
IR 48h
IR 120h
IR 192h
IR 288h
CTRL 0h
CTRL 48h
CTRL 120h
CTRL 192h
CTRL 288h
RB S807/811
RB S780
5sec
12/10/19
S807/811
S780

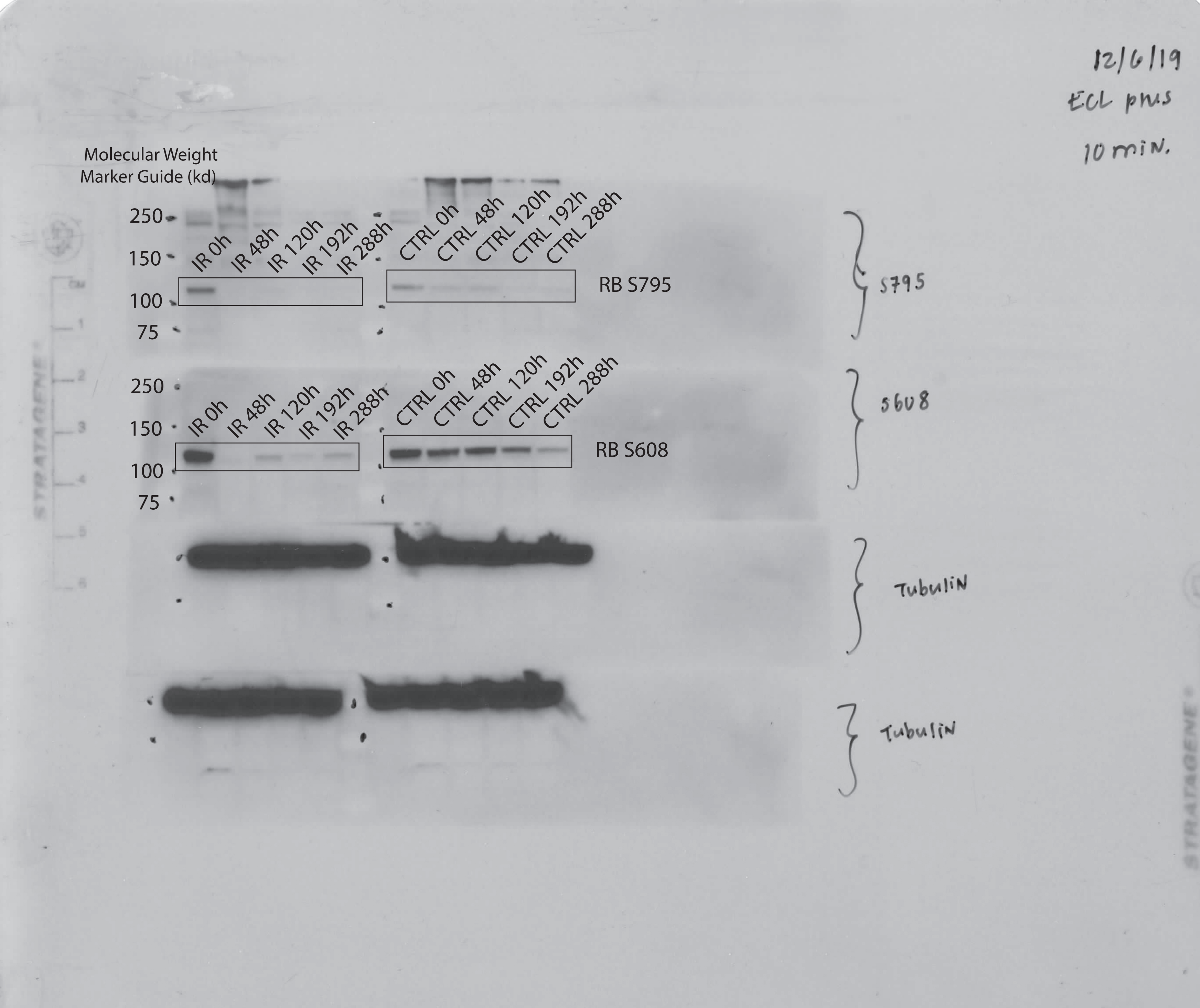
12/6/19
ECL plus
10 min.
Molecular Weight Marker Guide (kd)
250
150
100
75
IR 0h
IR 48h
IR 120h
IR 192h
IR 288h
CTRL 0h
CTRL 48h
CTRL 120h
CTRL 192h
CTRL 288h
RB S795
S795
250
150
100
75
IR 0h
IR 48h
IR 120h
IR 192h
IR 288h
CTRL 0h
CTRL 48h
CTRL 120h
CTRL 192h
CTRL 288h
RB S608
S608
Tubulin
Tubulin

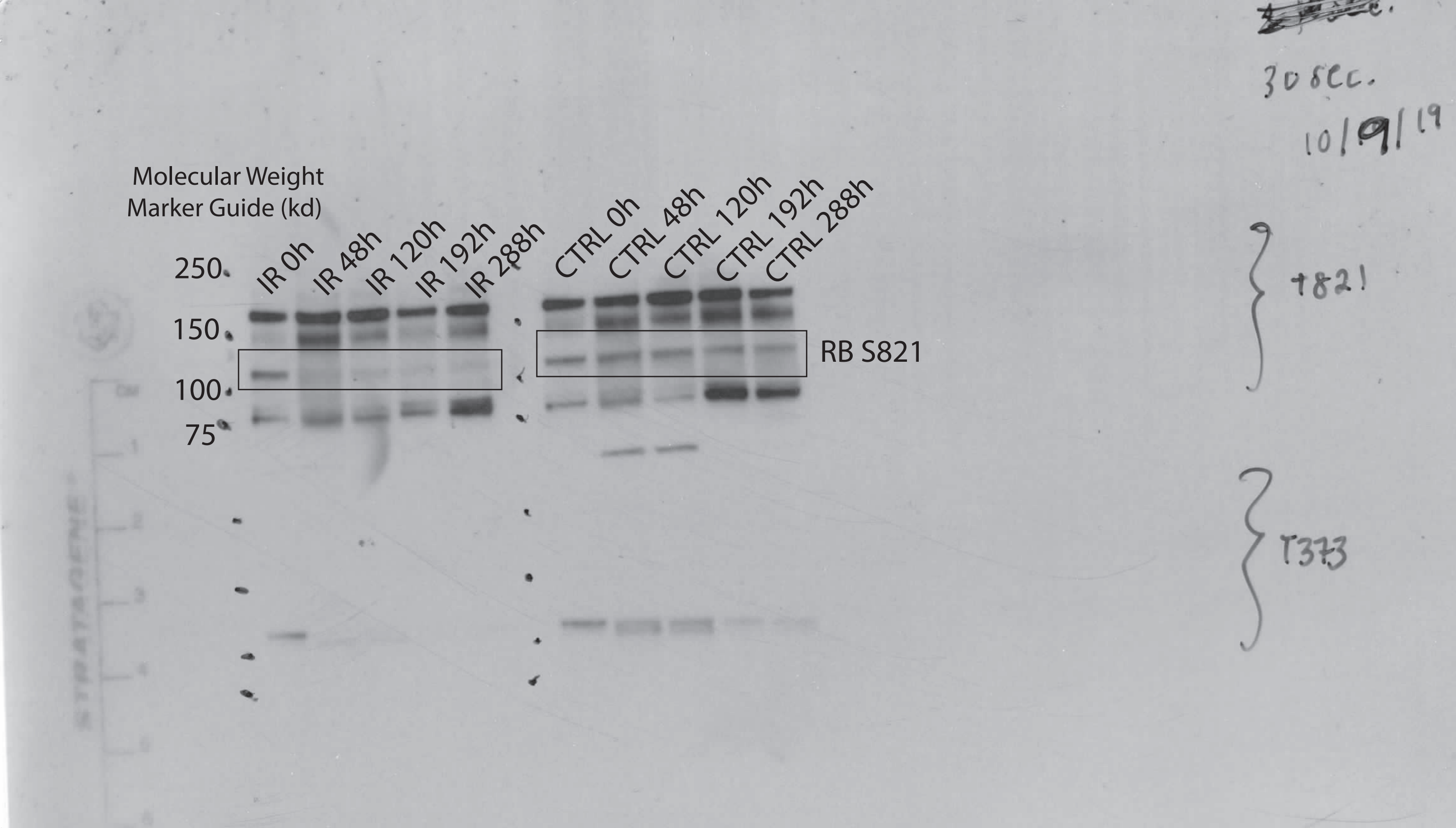
Molecular Weight
Marker Guide (kd)
250
150
100
75
IR 0h
IR 48h
IR 120h
IR 192h
IR 288h
CTRL 0h
CTRL 48h
CTRL 120h
CTRL 192h
CTRL 288h
RB S821
30 sec.
10/9/19
T821
T373

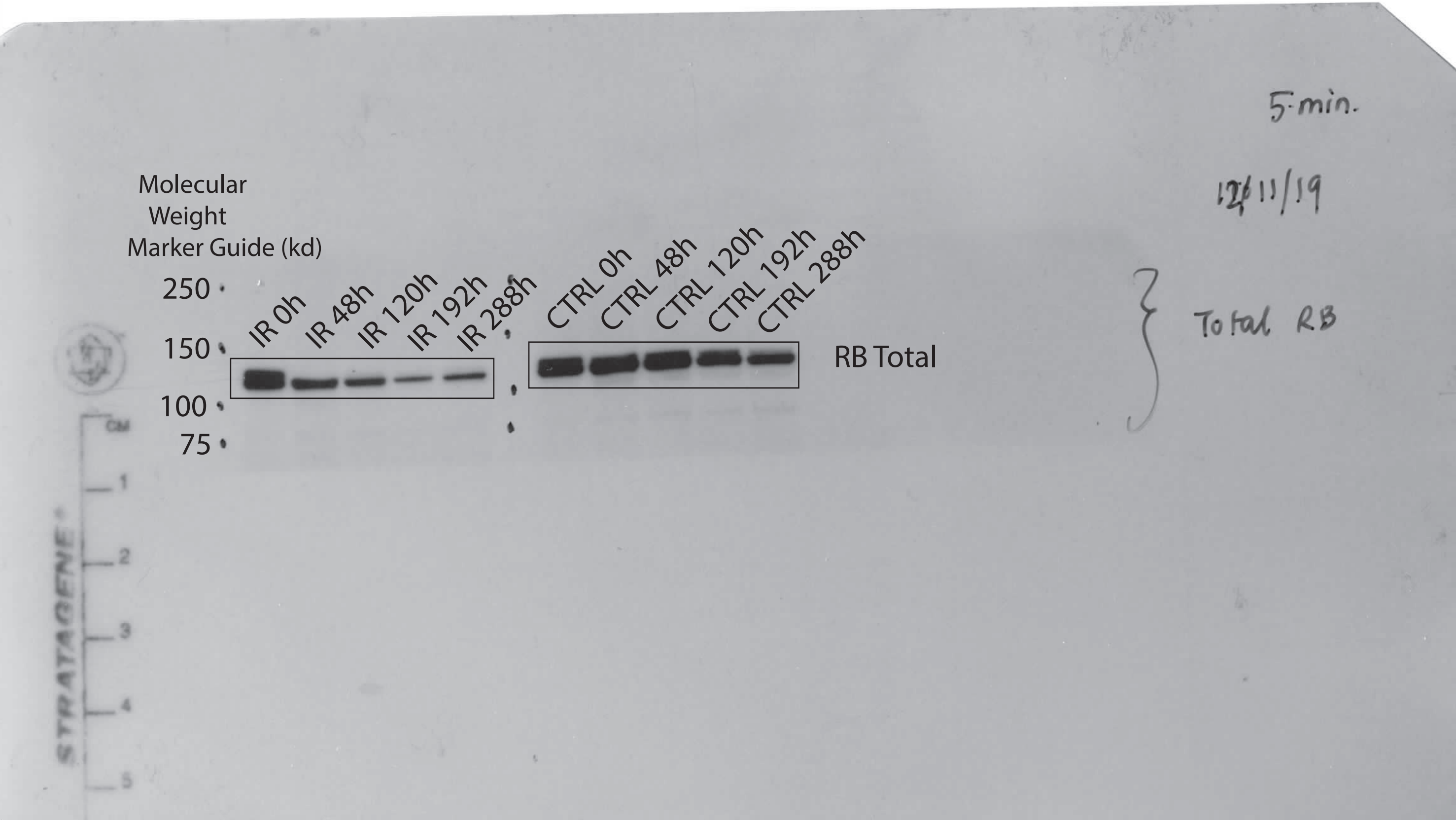
Molecular Weight Marker Guide (kd)
250
150
100
75
IR 0h
IR 48h
IR 120h
IR 192h
IR 288h
CTRL 0h
CTRL 48h
CTRL 120h
CTRL 192h
CTRL 288h
RB Total
5 min.
Total RB
STRATAGENE

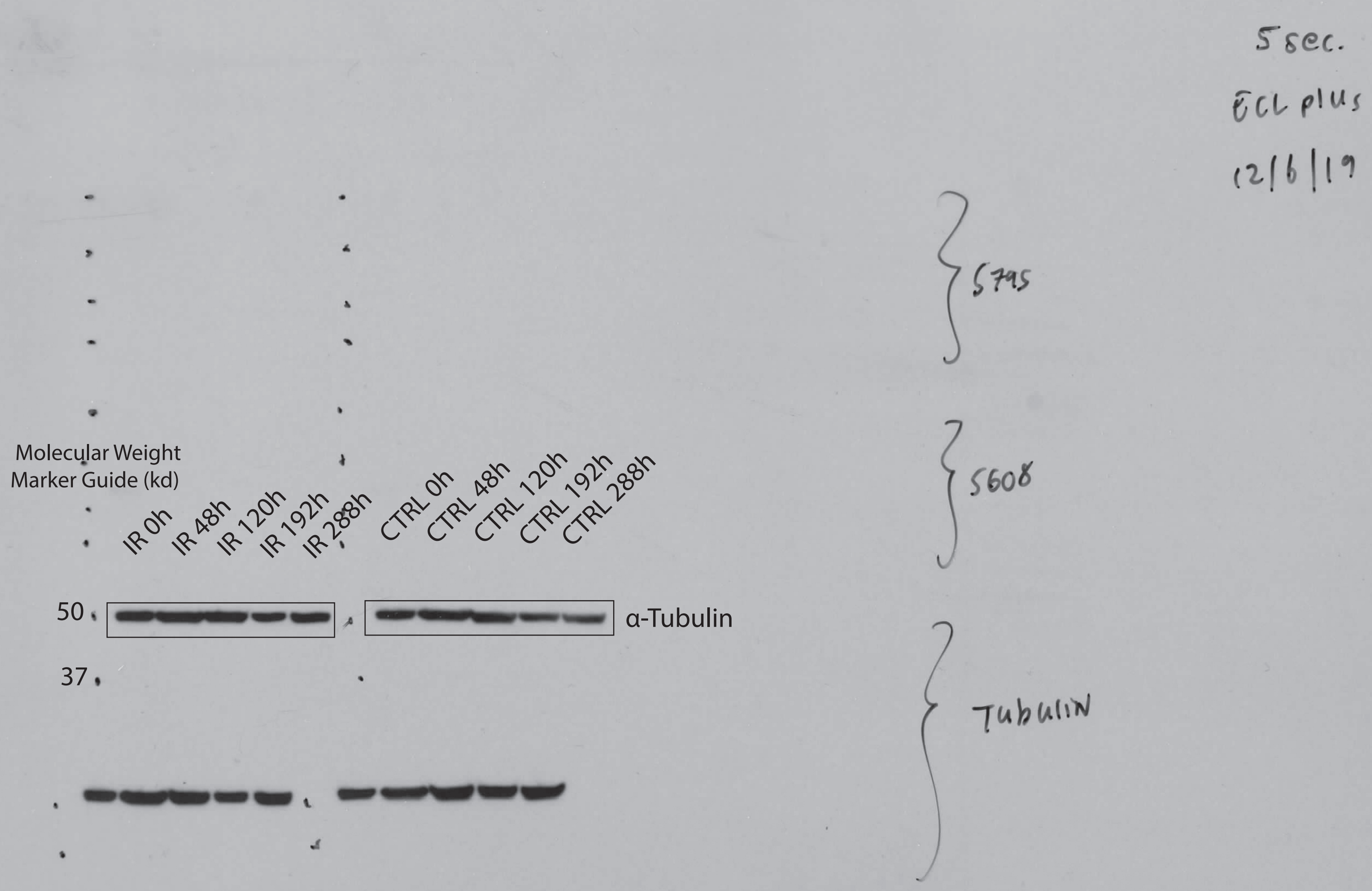
5 sec.
ECL plus
12/6/19
5795
5608
Molecular Weight
Marker Guide (kd)
IR 0h
IR 48h
IR 120h
IR 192h
IR 288h
CTRL 0h
CTRL 48h
CTRL 120h
CTRL 192h
CTRL 288h
50
α-Tubulin
37
Tubulin

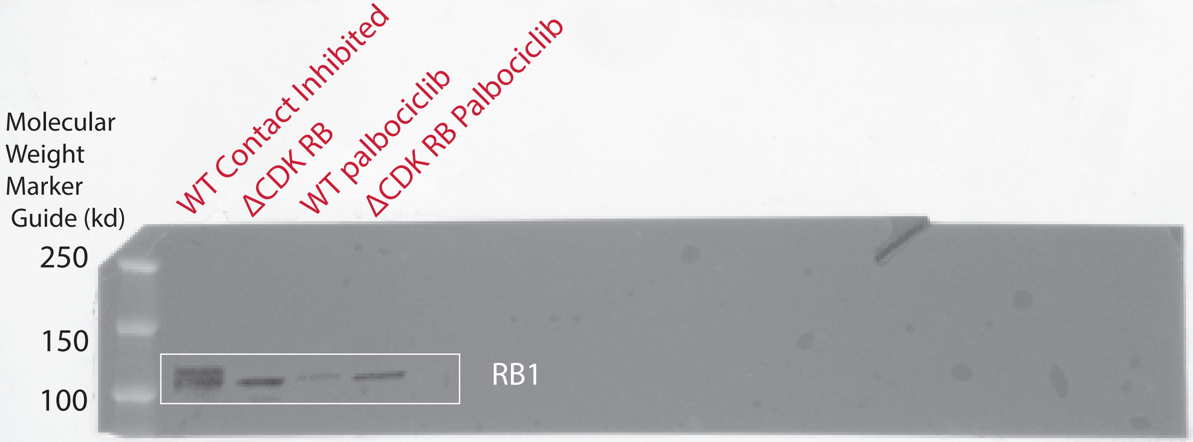
Molecular
Weight
Marker
Guide (kd)
250
150
100
WT Contact Inhibited
ΔCDK RB
WT palbociclib
ΔCDK RB Palbociclib
RB1

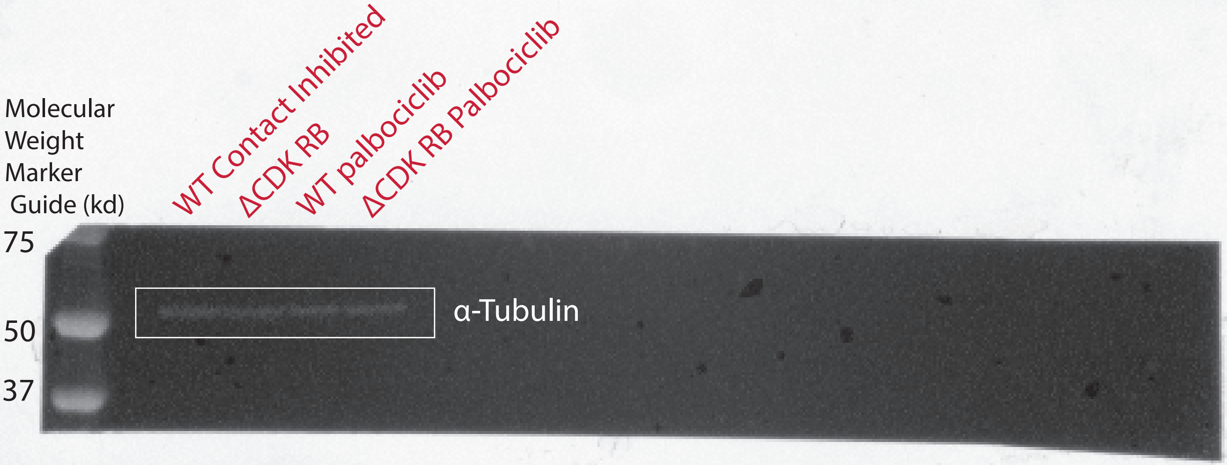
Molecular
Weight
Marker
Guide (kd)
WT Contact Inhibited
ΔCDK RB
WT palbociclib
ΔCDK RB Palbociclib
75
50
37
α-Tubulin
