# Supplementary material for: Active RB causes visible changes in nuclear organization
Source: J Cell Biol. 2022 Jan 12;221(3):e202102144. doi: 10.1083/jcb.202102144 (PMC8759594; doi:10.1083/jcb.202102144)
Supplement: SourceData FS3 — contains original blots for Fig. S3. [file JCB_202102144_SourceDataFS3.pdf]

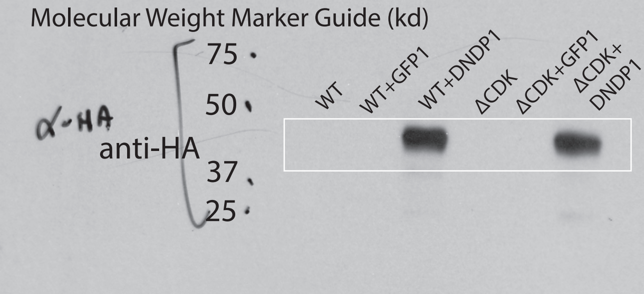
Molecular Weight Marker Guide (kd)
75
50
37
25
α-HA
anti-HA
WT
WT+GFP1
WT+DNDP1
ΔCDK
ΔCDK+GFP1
ΔCDK+
DNDP1

Molecular Weight Marker Guide (kd)

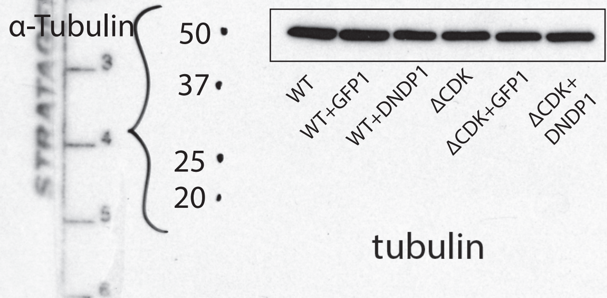

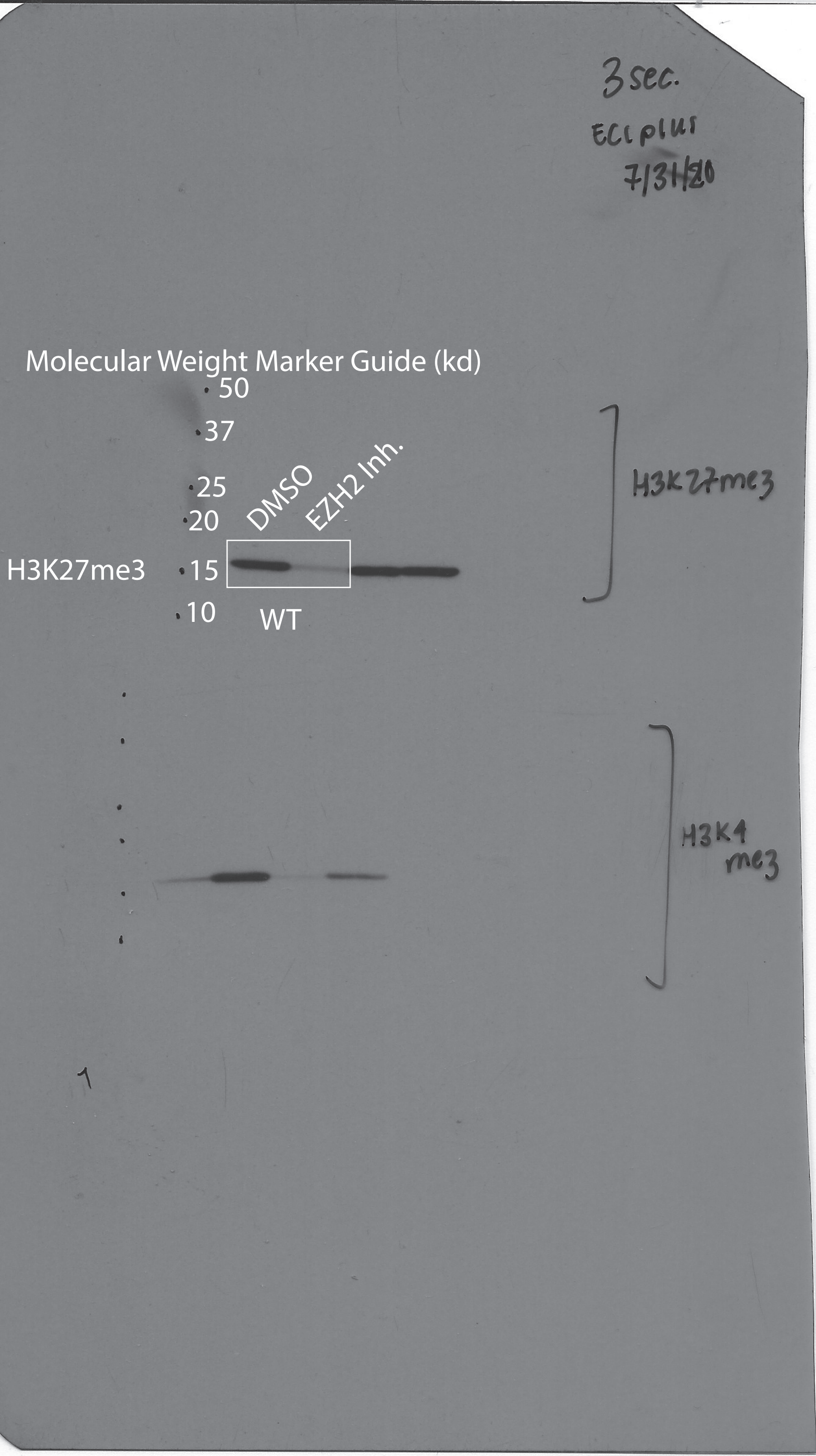
3 sec.
ECL plus
7/31/20
Molecular Weight Marker Guide (kd)
50
37
25
20
15
10
DMSO
EZH2 Inh.
H3K27me3
WT
H3K27me3
H3K4 me3
1

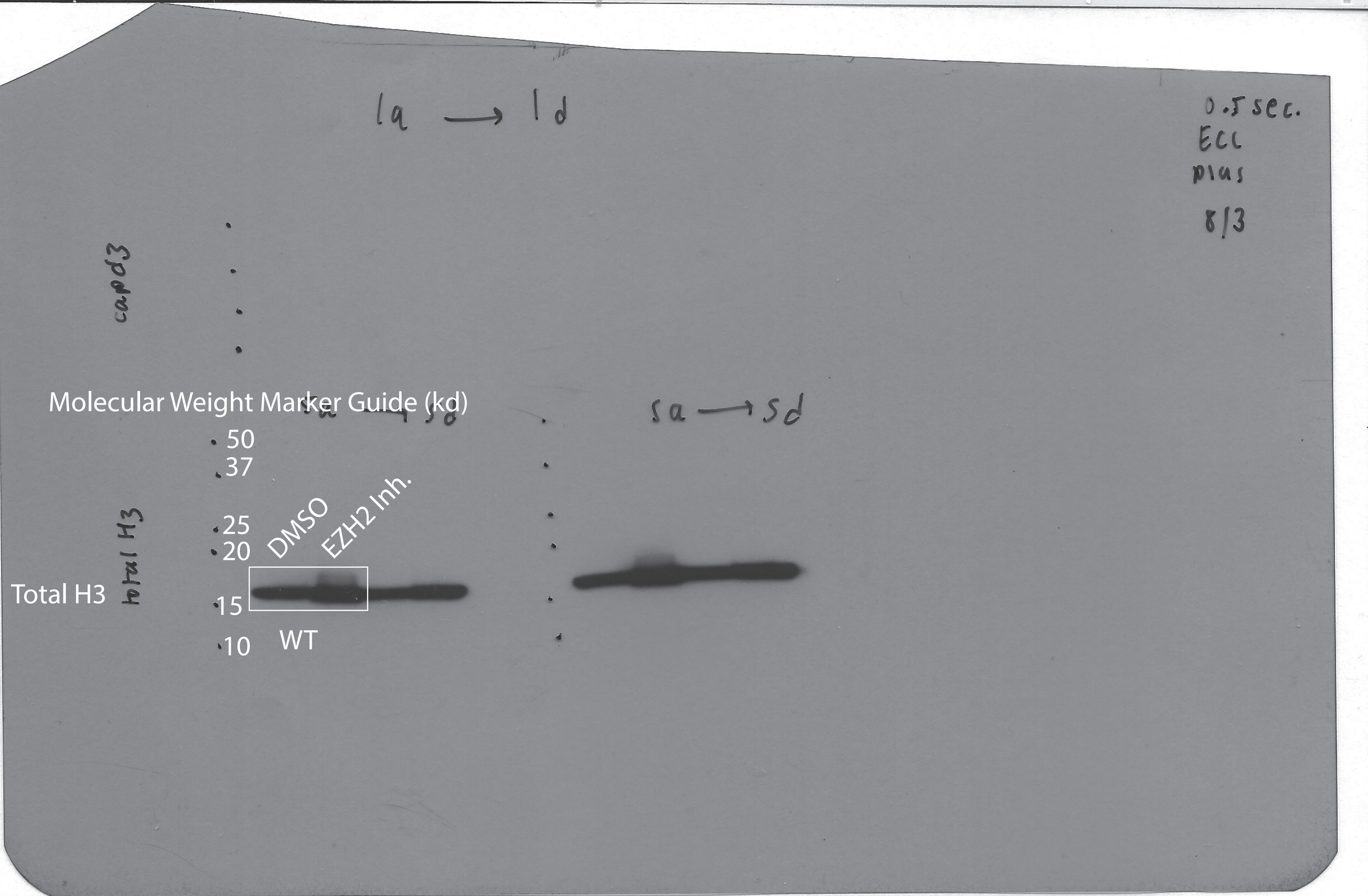
1a → 1d
0.5 sec.
ECL
plus
8/3
capd3
Molecular Weight Marker Guide (kd)
5a → 5d
50
37
25
20
15
10
DMSO
EZH2 Inh.
Total H3
total H3
WT

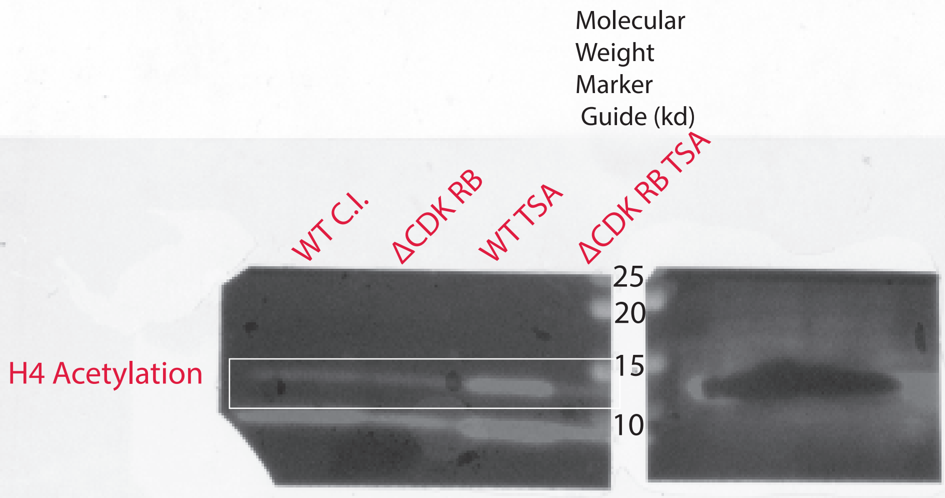
Molecular
Weight
Marker
Guide (kd)
WT C.I.
ΔCDK RB
WT TSA
ΔCDK RB TSA
25
20
15
10
H4 Acetylation

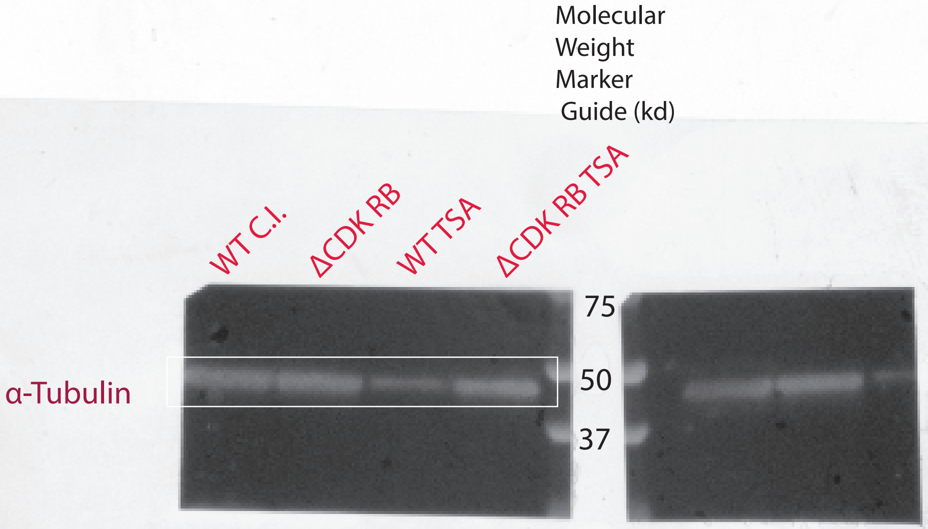
Molecular
Weight
Marker
Guide (kd)
WT C.I.
ΔCDK RB
WT TSA
ΔCDK RB TSA
75
50
37
α-Tubulin

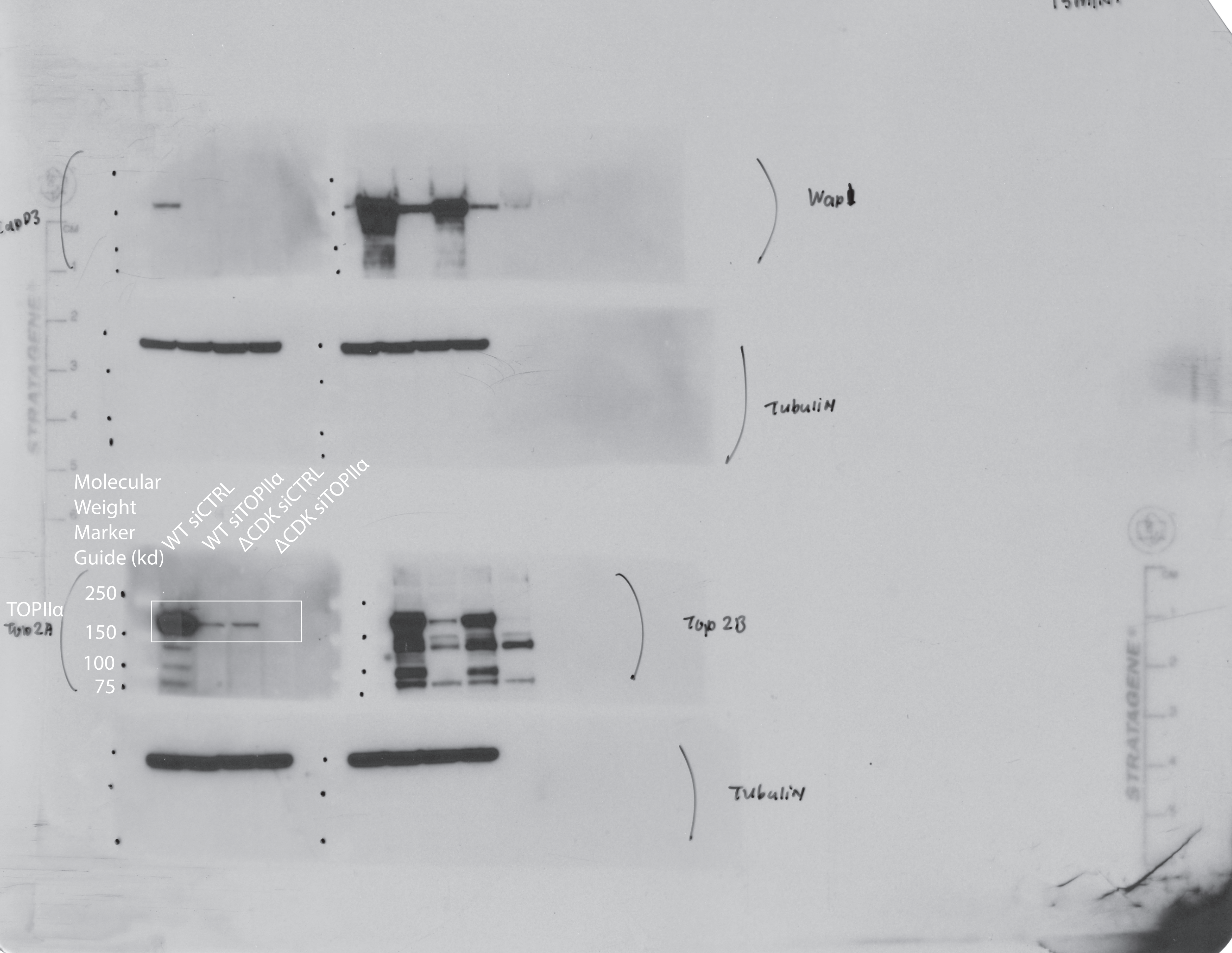
Wapl
Tubulin
Molecular
Weight
Marker
Guide (kd)
WT siCTRL
WT siTOPIIα
ΔCDK siCTRL
ΔCDK siTOPIIα
250
150
100
75
TOPIIα
Top2A
Top 2B
Tubulin
STRATAGENE

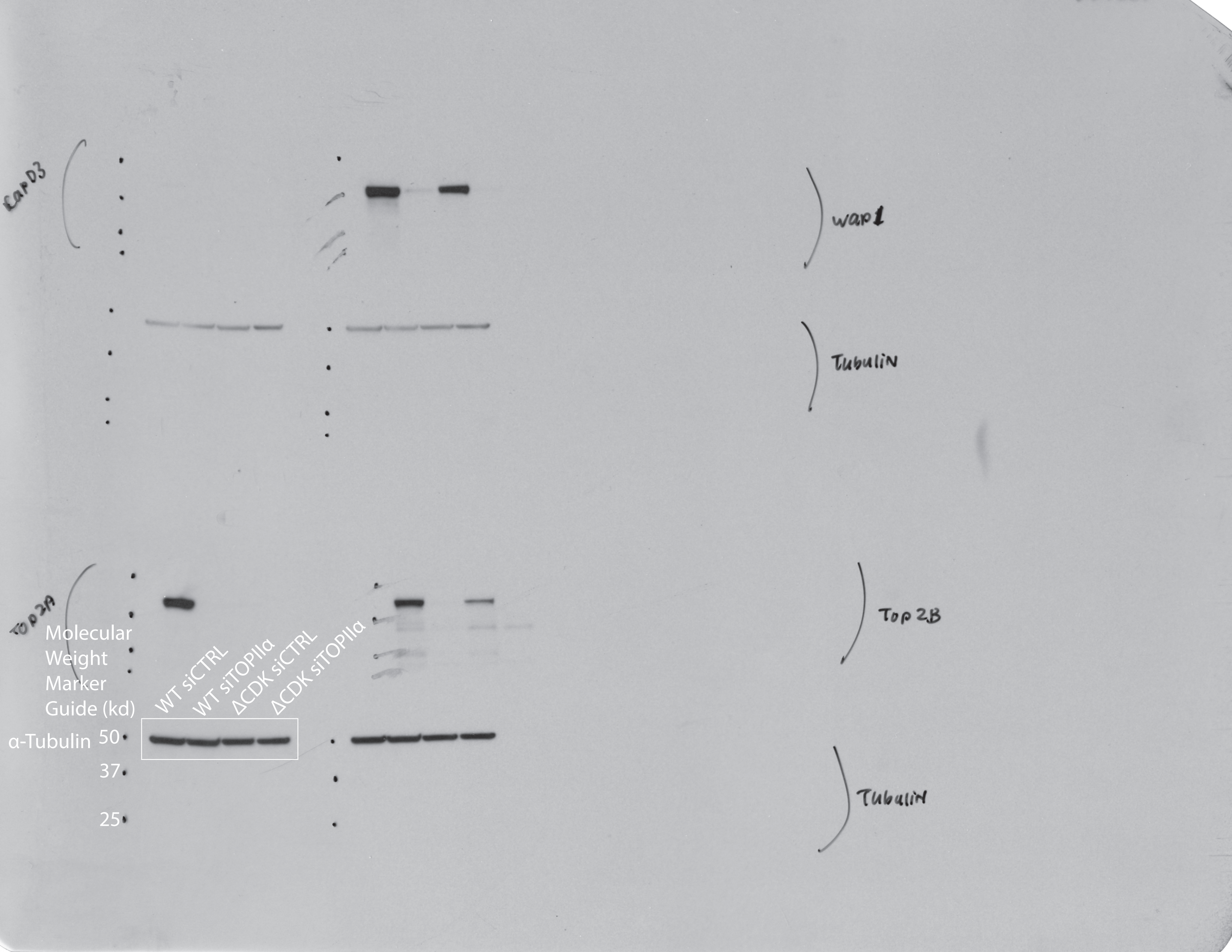
CapD3
wap1
Tubulin
Top2A
Top 2B
Molecular Weight Marker Guide (kd)
WT siCTRL
WT siTOPIIα
ΔCDK siCTRL
ΔCDK siTOPIIα
α-Tubulin
50
37
25
Tubulin

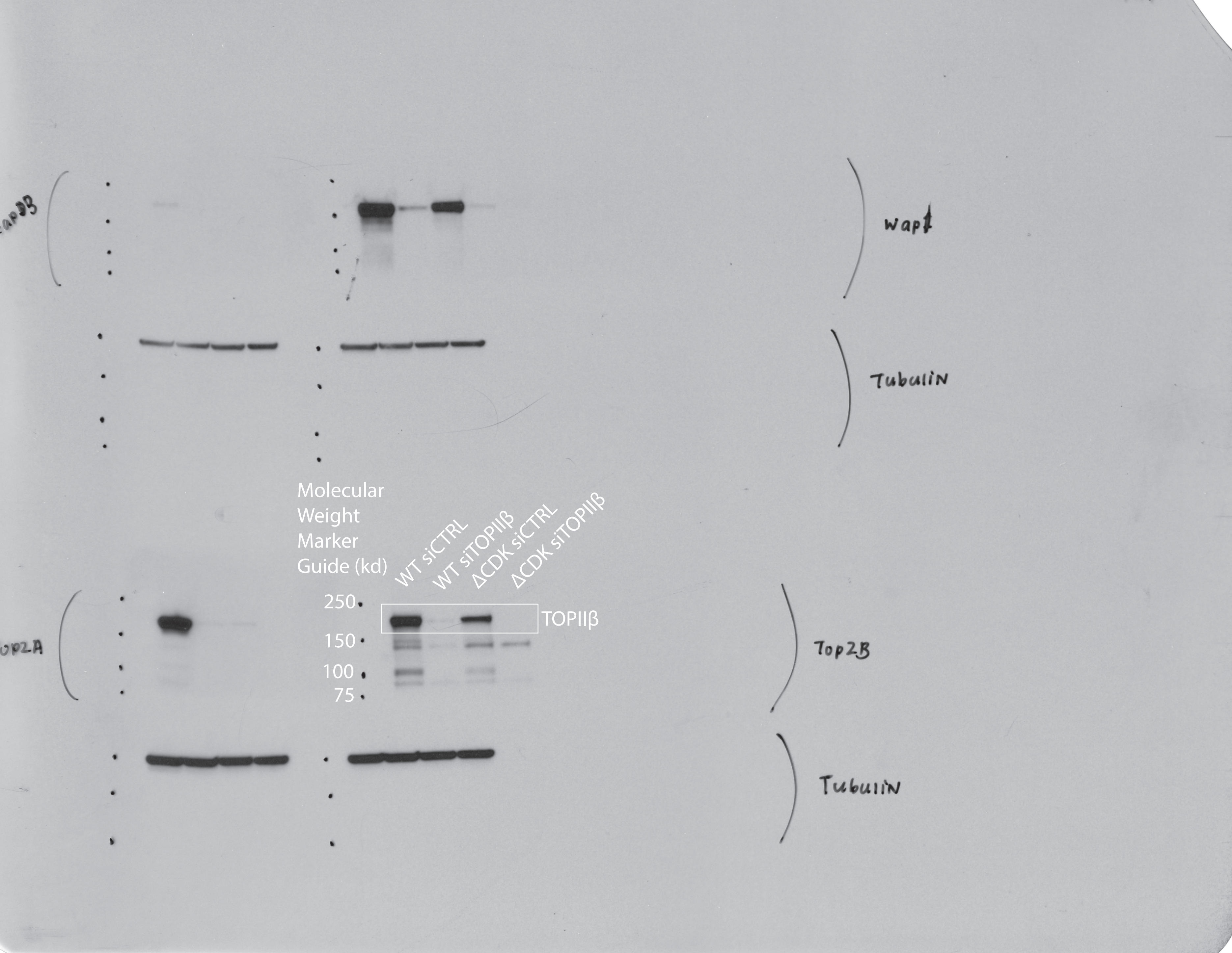
Molecular Weight Marker Guide (kd)
WT siCTRL
WT siTOPIIβ
ΔCDK siCTRL
ΔCDK siTOPIIβ
250
150
100
75
TOPIIβ
Tubulin
Top2B
Tubulin

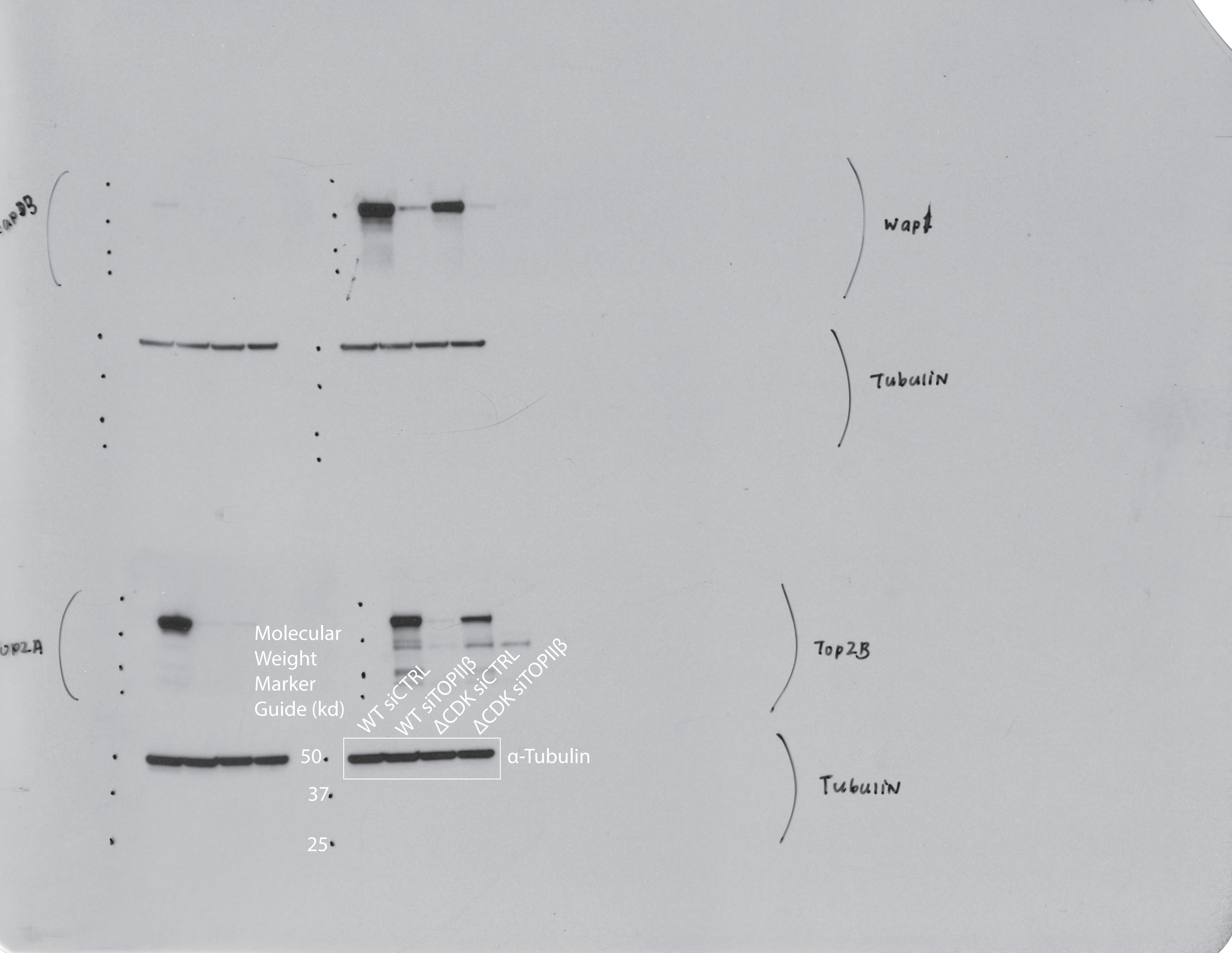
Molecular Weight Marker Guide (kd)
50
37
25
WT siCTRL
WT siTOPIIβ
ΔCDK siCTRL
ΔCDK siTOPIIβ
α-Tubulin
Tubulin
Top2B
Tubulin
