# Supplementary material for: Active RB causes visible changes in nuclear organization
Source: J Cell Biol. 2022 Jan 12;221(3):e202102144. doi: 10.1083/jcb.202102144 (PMC8759594; doi:10.1083/jcb.202102144)
Supplement: SourceData FS4 — contains original blots for Fig. S4. [file JCB_202102144_SourceDataFS4.pdf]

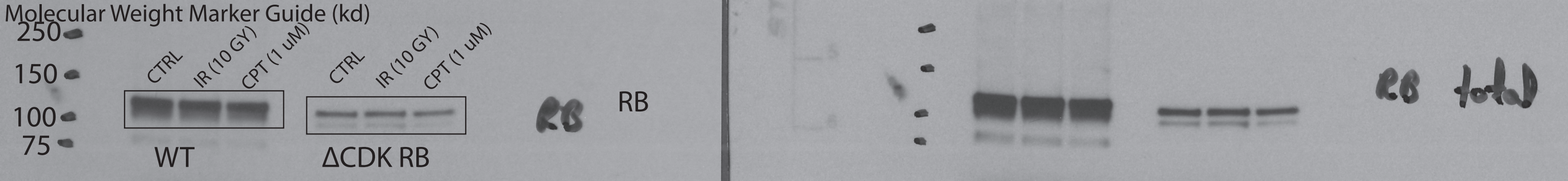
Molecular Weight Marker Guide (kd)
250
150
100
75
CTRL
IR (10 GY)
CPT (1 uM)
CTRL
IR (10 GY)
CPT (1 uM)
RB
RB
WT
ΔCDK RB
RB total

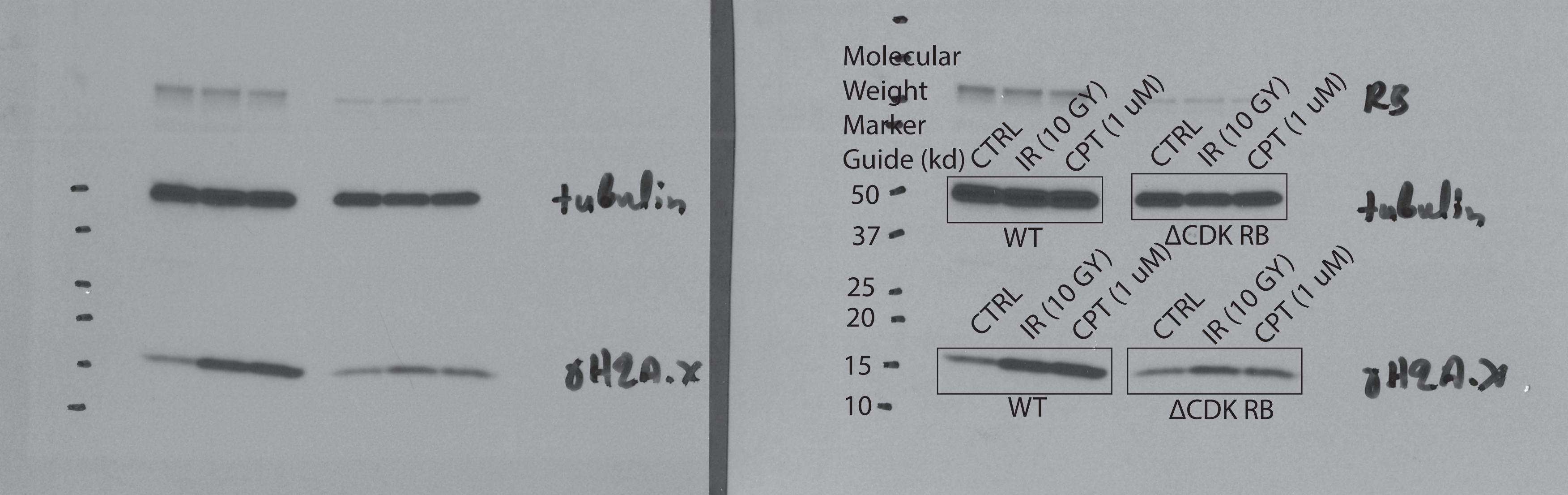
tubulin
γH2A.x
Molecular
Weight
Marker
Guide (kd)
50
37
25
20
15
10
CTRL
IR (10 GY)
CPT (1 uM)
CTRL
IR (10 GY)
CPT (1 uM)
RB
tubulin
WT
ΔCDK RB
CTRL
IR (10 GY)
CPT (1 uM)
CTRL
IR (10 GY)
CPT (1 uM)
WT
ΔCDK RB
γH2A.x

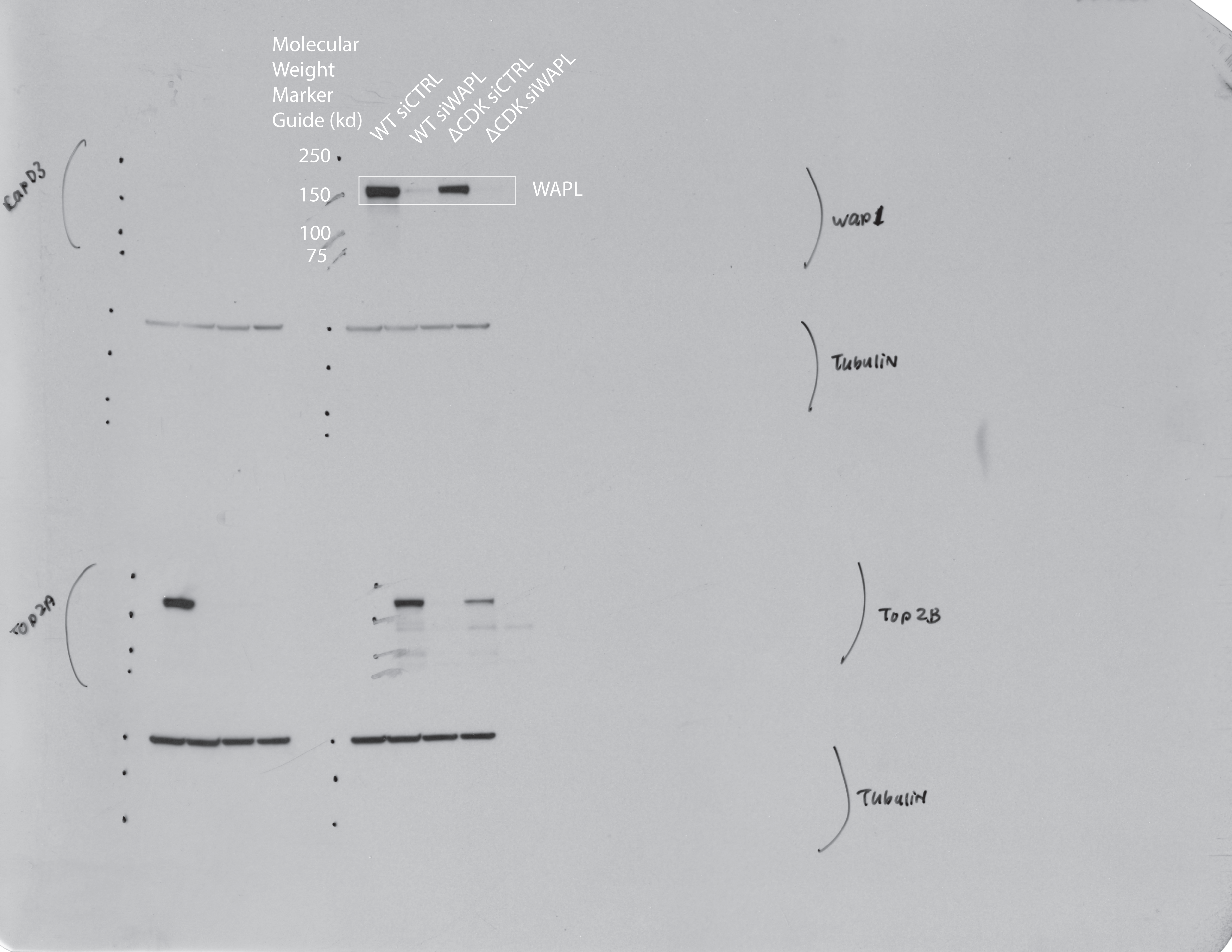
Molecular Weight Marker Guide (kd)
WT siCTRL
WT siWAPL
ΔCDK siCTRL
ΔCDK siWAPL
250
150
100
75
WAPL
Tubulin
Top 2B
Tubulin

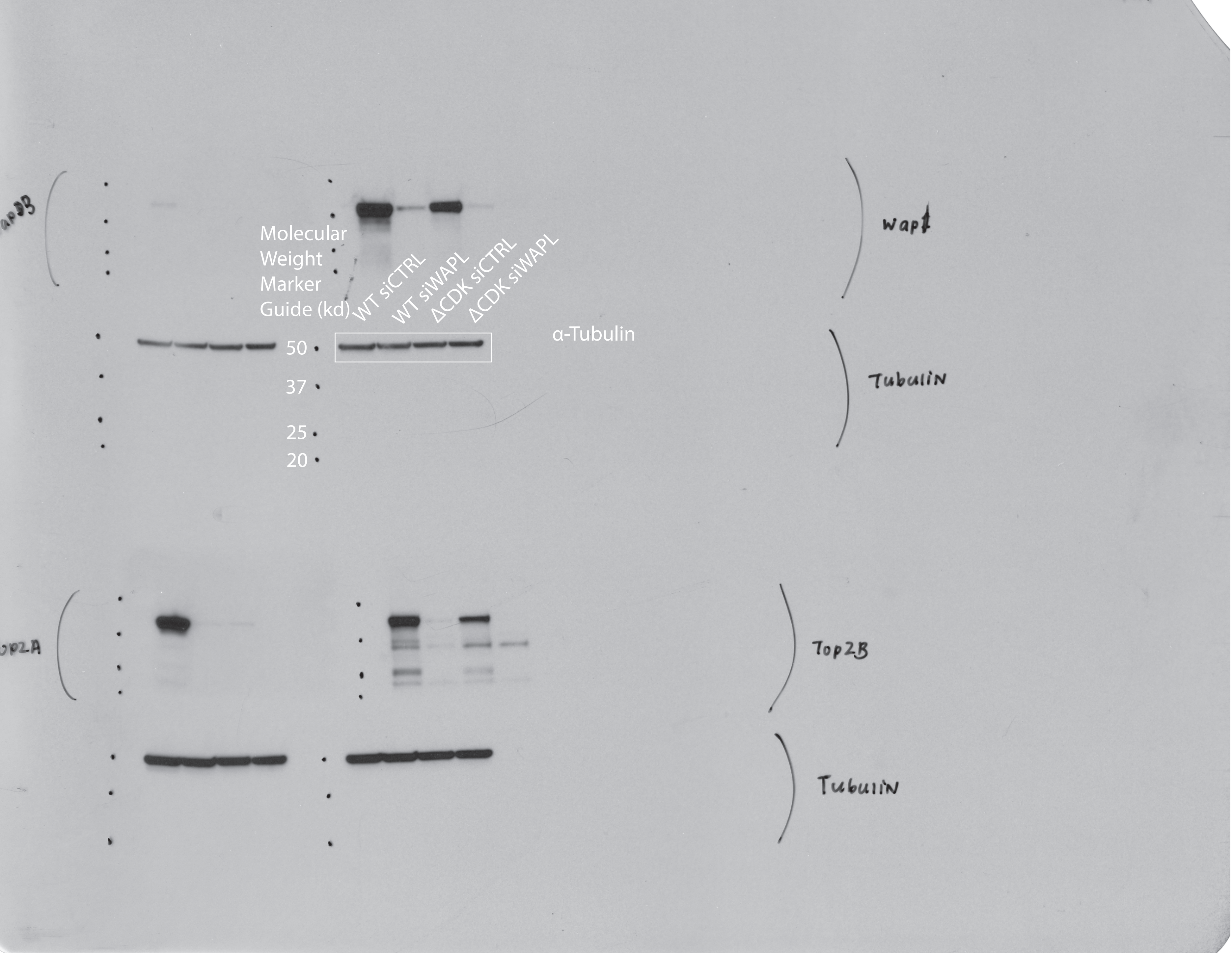

Molecular
Weight
Marker
Guide (kd)
WT siCTRL
WT siWAPL
ΔCDK siCTRL
ΔCDK siWAPL
α-Tubulin
50
37
25
20
Tubulin
Top2B
Tubulin

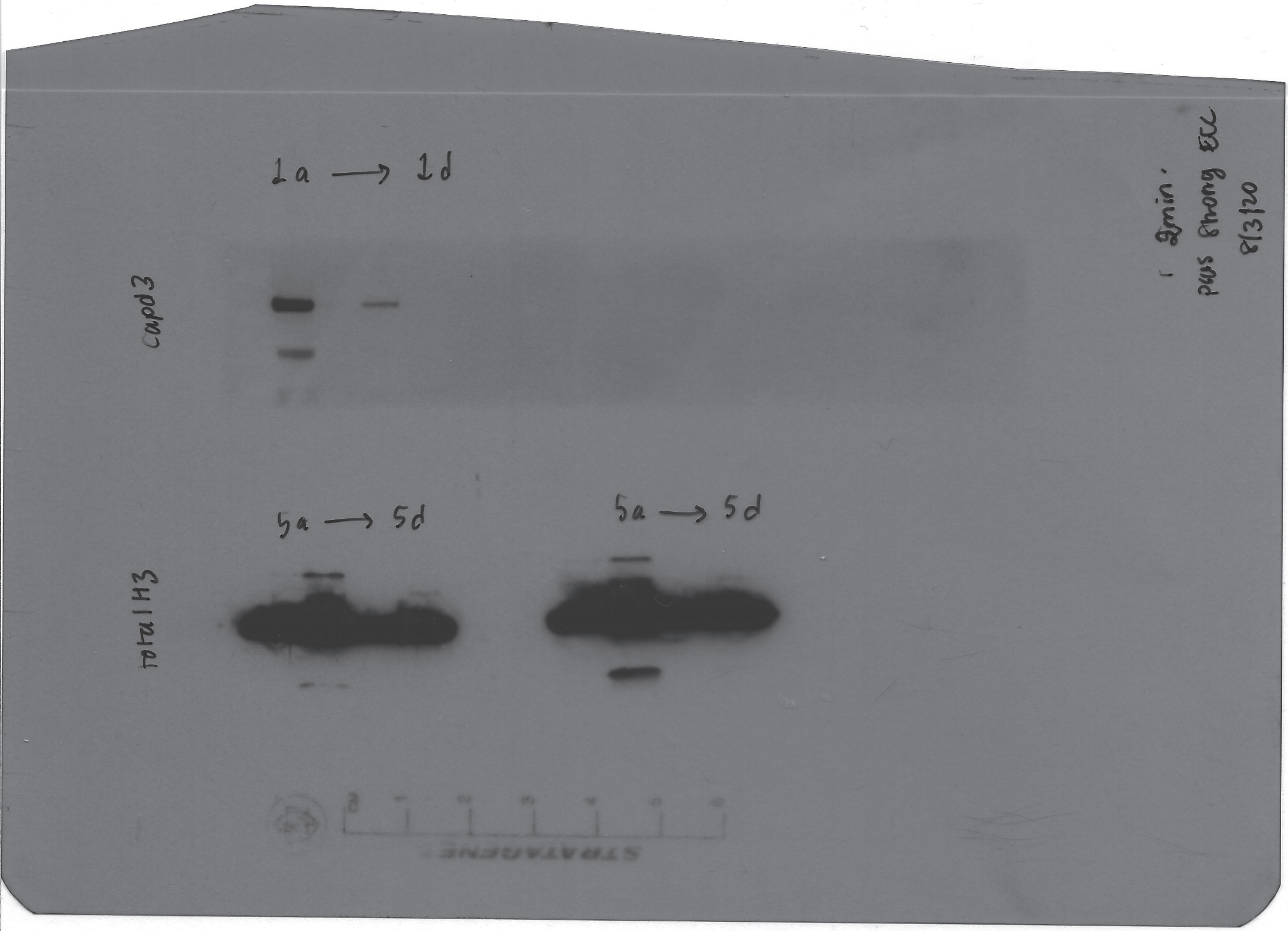
1a → 1d
capd3
5a → 5d
5a → 5d
total H3
plus strong ECL
8/3/20

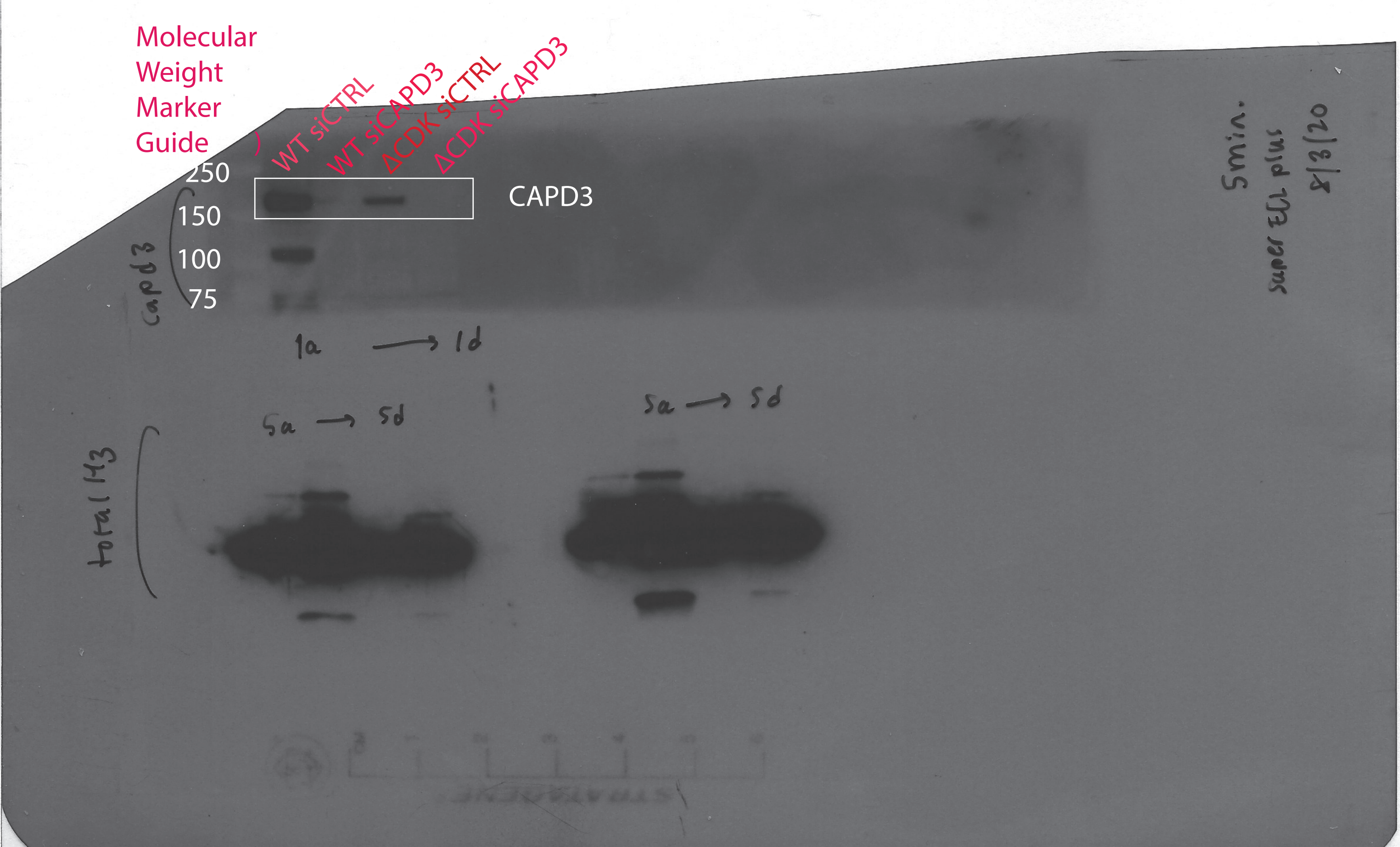
Molecular Weight Marker Guide
250
150
100
75
WT siCTRL
WT siCAPD3
ΔCDK siCTRL
ΔCDK siCAPD3
CAPD3
capd3
1a → 1d
5a → 5d
5a → 5d
total H3
5min.
super ECL plus
8/3/20

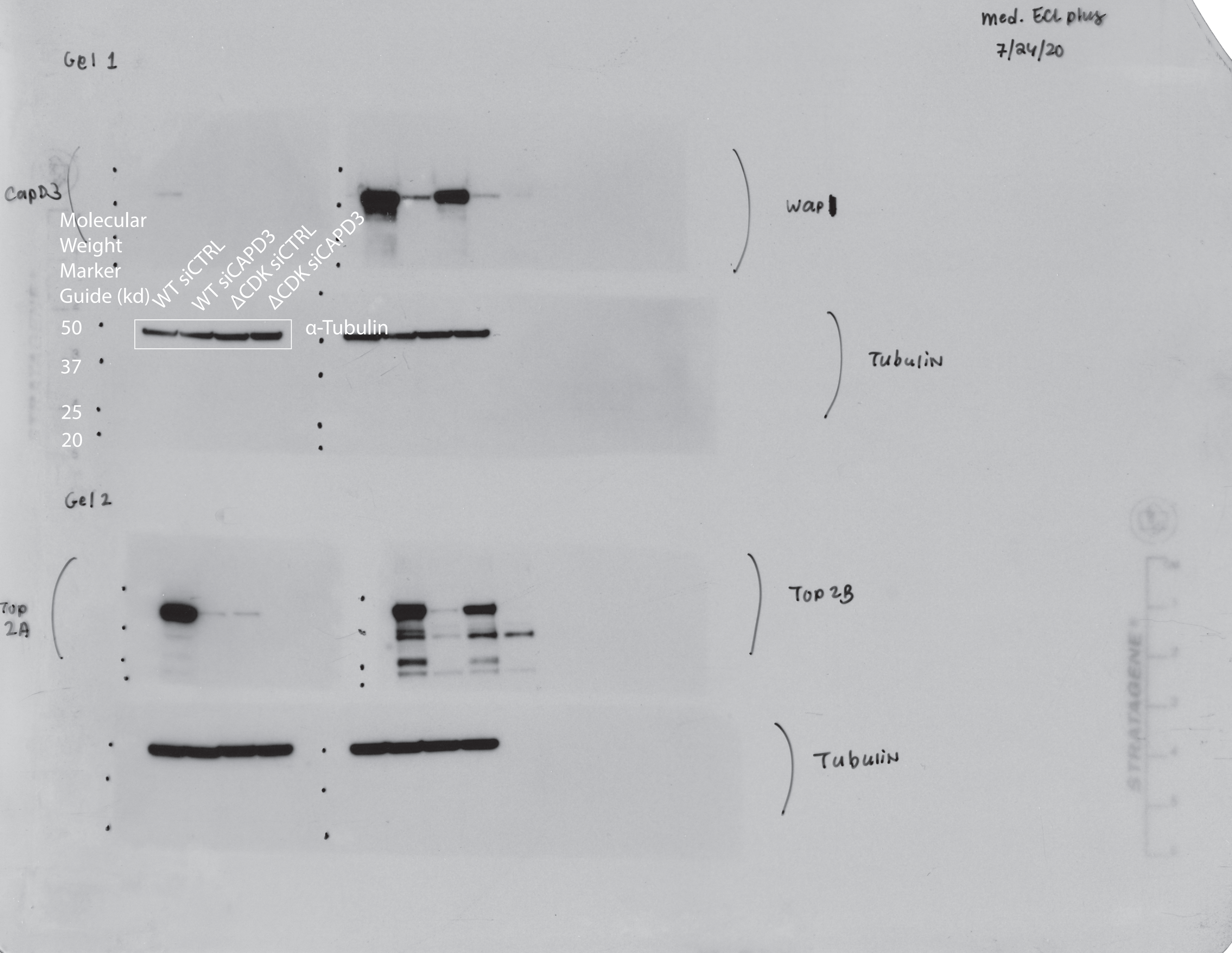

med. ECL plus
7/24/20
Gel 1
CapD3
Molecular
Weight
Marker
Guide (kd)
WT siCTRL
WT siCAPD3
ΔCDK siCTRL
ΔCDK siCAPD3
50
37
25
20
α-Tubulin
Wapl
Tubulin
Gel 2
Top
2A
Top 2B
Tubulin
